# Computational Insights on the Chemical Reactivity of Functionalized and Crosslinked Polyketones to Cu^2+^ Ion for Wastewater Treatment

**DOI:** 10.3390/polym15153157

**Published:** 2023-07-25

**Authors:** Daniela E. Ortega, Diego Cortés-Arriagada, Rodrigo Araya-Hermosilla

**Affiliations:** 1 Centro Integrativo de Biología y Química Aplicada (CIBQA), Facultad de Salud, Universidad Bernardo O’Higgins, General Gana 1702, Santiago 8370854, Chile; 2 Programa Institucional de Fomento a la Investigación, Desarrollo e Innovación (PIDi), Universidad Tecnológica Metropolitana, Ignacio Valdivieso 2409, San Joaquín, Santiago 8940577, Chile; dcortes@utem.cl (D.C.-A.); rodrigo.araya@utem.cl (R.A.-H.)

**Keywords:** polyketone, crosslinked resins, water treatment, Cu^2+^ uptake, DFT calculations

## Abstract

Today, the high concentrations of copper found in water resources result in an urgent problem to solve since human health and aquatic ecosystems have been affected. Functionalized crosslinked polyketone resins (XLPK) have demonstrated high performance for the uptake of heavy metals in water solutions. In addition, its green chemical synthesis makes these resins very attractive as sorbents for metal ions contained in wastewater. XLPK are not soluble in aqueous media and do not require any catalyst, solvent, or harsh conditions to carry out the uptake process. In this paper, a series of functionalized XLPK with pending amino-derivatives namely; butylamine (BA), amino 2-propanol (A2P), 4-(aminomethyl) benzoic acid (HAMC), 6-aminohexanoic acid (PAMBA), and 1,2 diamino propane (DAP) directly attached to the pyrrole backbone of the polymers and crosslinked by di-amine derivatives was investigated using Density Functional Theory (DFT) calculations. Our computational analysis revealed that dipole-dipole interactions played a crucial role in enhancing the adsorption of Cu^2+^ ions onto XLPKs. The negatively charged ketone moieties and functional groups within XLPKs were identified as key adsorption sites for the selective binding of Cu^2+^ ions. Additionally, we found that XLPKs exhibited strong electrostatic interactions primarily through the –NH_2_ and –C=O groups. Evaluation of the adsorption energies in XLPK-Cu(II) complexes showed that the DAP-Cu(II) complex exhibited the highest stability, attributed to strong Cu(II)-N binding facilitated by the amino moiety (–NH_2_). The remaining XLPKs displayed binding modes involving oxygen atoms (Cu(II)-O) within the ketone moieties in the polymer backbone. Furthermore, the complexation and thermochemical analysis emphasized the role of the coordinator atom (N or O) and the coordinating environment, in which higher entropic effects involved in the adsorption of Cu^2+^ ions onto XLPKs describes a lower spontaneity of the adsorption process. The adsorption reactions were favored at lower temperatures and higher pressures. These findings provide valuable insights into the reactivity and adsorption mechanisms of functionalized and crosslinked polyketones for Cu^2+^ uptake, facilitating the design of high-performance polymeric resins for water treatment applications.

## 1. Introduction

Toxic heavy metals, such as Pb, Cr, As, Cd, Cu, and Hg, are naturally present in the environment [1]. However, anthropogenic activities such as explosives, mining/metallurgic, textile, and pesticides have increased the occurrence of heavy metals in aquatic environments [2,3]. Some main sources are untreated wastewaters that detrimentally affect the aquatic biota through direct contamination or trophic chain biomagnification [4]. Particularly, copper has risen high concern due to its occurrence in water bodies at high concentrations (2.5–10,000 ppm) due to increased industrial activities [3]. Among the different species of copper (Cu^0^, Cu^+^, Cu^2+^), Cu^2+^ has been identified as the most toxic one with several effects on human health [5]. The increased toxicity of Cu^2+^ can be attributed to its higher reactivity and ability to participate in electron transfer reactions having a higher affinity for binding to biological molecules, including proteins and enzymes, than Cu^+^, altering enzyme activity, and generating reactive oxygen species. Regulating and monitoring copper levels in aquatic environments is important to prevent adverse ecological consequences. Different techniques have been proposed to remove Cu^2+^ from wastewater before it reaches rivers and marine systems [3,6,7,8]. In a general context, membrane technologies [9,10,11], electrochemistry [12,13], solar catalysis [14], MOFs [15,16], polymer coagulation [17], and heavy metal absorption with different chemicals [18] and nanomaterials [19,20,21] techniques has been used for the Cu^2+^ uptake and other heavy metal treatment in water environments.

In this context, highly crosslinked (thermosets) polymer systems represent a breakthrough for scalable and insoluble materials applied as Cu^2+^ adsorbent in water [22,23]. The so-called hyper-cross-linked polymers (HCP) allow obtaining microporous forms through their synthesis, thus presenting high surface area, simple operating conditions, and low cost of reagents. These crosslinked polymers offer many superior properties compared to membranes or hydrogels regarding mechanical strength, dimensional stability at elevated temperatures, and solvent resistance [24]. Moreover, these polymeric networks can be synthesized by several monomers, crosslinkers, and functionality goes according to the chemical groups contained in the original reagents [23]. Accordingly, the most effective chemical groups for metal ion remotion have been reported as carboxyl, phosphoric, and sulfuric groups [25]. In addition, chelating agents containing nitrogen atoms have also been reported as excellent chemical species for metal ion remotion [26,27,28,29].

**Scheme 1:**
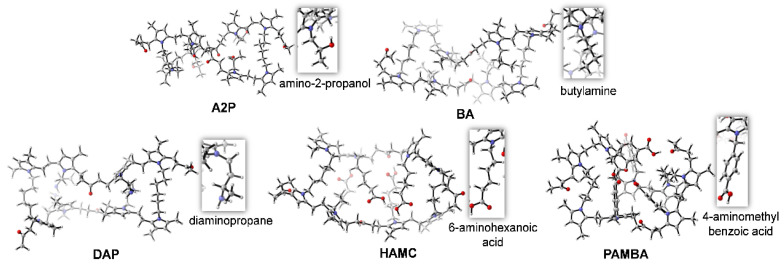
Schematic representation of the functionalized crosslinked polyketone resins (XLPK) with pending amino-derivatives amino 2-propanol (A2P), butylamine (BA), 1,2 diamino propane (DAP), 6-aminohexanoic acid (HAMC), and 4-(aminomethyl) benzoic acid (PAMBA). N in blue, O in red, C in grey and H in white.

In this regard, polyketone (PK) and its chemical modification, crosslinking, and potential use as resins for water treatment has been experimentally proposed [30,31,32]. Alternating aliphatic polyketones [33] are perfect starting polymers for highly crosslinked resins through their chemical modification via the Paal–Knorr reaction. This is related to the tolerance of this reaction towards many functional groups, particularly sterically hindered amino ones [31], as is shown in Scheme 1. The Paal–Knorr synthetic pathway offers several advantages, such as high yield under relatively mild conditions (100 °C) and fast reaction kinetics (4 h) even without any catalyst, with water as the only by-product. For instance, the easy chemical modification and low production costs allowed the preparation of polymeric membranes for emulsion separation [34]. Notably, the chemical modification of PK with nitrogen-containing compounds has been demonstrated to be a versatile way to obtain insoluble polymer networks bearing functional groups capable of chelating metal ions in aqueous solutions [30,31]. Particularly, diamine molecules directly grafted on the pyrrolic PK backbone chain allowed the formation of three-dimensional network structures after being crosslinked, and the properties of the resulting product were easily tuned by changing the crosslinking density.

Indeed, the crosslinking density in polyketones allows for tailoring many aspects of their thermo-mechanical properties, surface area, and swelling [35]. Particularly, highly crosslinked PK systems rendered networks with high mechanical properties such as hardness, modulus, and remarkable solvent resistance. The latter implies that there is low intermolecular space. On the contrary, polymer networks with low crosslinking densities demonstrate high toughness and significant intermolecular space. This characteristic can be advantageous, as low crosslinking imparts flexibility to the materials, along with an enhanced swelling behavior. Therefore, a trade-off between high and low crosslinking density values and functionality is necessary to achieve the required properties for an application such as crosslinking resins for wastewater treatment.

In this article, we present a computational study based on Density Functional Theory (DFT) calculations to gain insight into the adsorption phenomenon of crosslinked polyketone resins (XLPK) with pending amino-derivatives such as; butylamine (BA), amino 2-propanol (A2P), 4-(aminomethyl) benzoic acid (HAMC), 6-aminohexanoic acid (PAMBA), and 1,2 diamino propane (DAP), displayed in Scheme 1, for effective adsorption of Cu^2+^ ions in water treatment. Some DFT studies have been reported by exploring various new and efficient materials searching for adsorbing heavy metals in an aqueous environment by predicting the reactivity of the chelating binding sites and strengths [36,37,38], which are often difficult to be accessed experimentally. However, to our knowledge, no scientific article based on DFT calculations has been published on XLPK.

Reactivity studies utilizing DFT calculations have proven highly valuable in understanding and predicting pollutant adsorption processes, offering crucial insights for environmental remediation efforts. For instance, DFT calculations provide a detailed understanding of the electronic structure, energetics, and properties of chemical systems by identifying key molecular descriptors and factors that influence reactivity, offering a unique perspective on the intrinsic nature of molecular systems [39,40,41,42]. These studies elucidate the underlying mechanisms, provide quantitative estimates of adsorption energies, and guide the design of efficient adsorbent materials.

This research aims to comprehensively investigate the reactivity of polyketone resins with pending amino derivatives toward Cu^2+^ ions. The main objective of this study is to accurately determine the adsorption conformations and elucidate the binding mechanisms between XLPK and Cu^2+^ ions. Additionally, the research seeks to identify and analyze the factors that play a crucial role in influencing the reactivity of XLPK toward Cu^2+^ uptake. The outcomes of this study are expected to yield valuable insights that can be used in the design and development of advanced polymeric resins with enhanced performance for water treatment applications.

## 2. Computational Methods

The functionalized crosslinked polyketone resins (XLPKs) according Scheme 1 were designed as cluster models; A2P (C_92_N_8_H_136_O_8_), BA (C_96_N_8_H_144_O_4_), DAP (C_93_N_12_H_142_O_4_), HAMC (C_105_N_8_H_154_O_12_), and PAMBA (C_112_N_8_H_136_O_12_) determining their morphology by molecular dynamics simulations with the Universal Force Field (UFF) [43] and using an NVT ensemble in the Forcite module of Materials Studio [44]. XLPKs were initially minimized at 500 K for 5 ns, then the resulting structures were solvated in a cubic box and minimized for 5 ns with target temperatures of 500, 400, and 300 K. Then, all the systems were fully optimized at the DFT level using ORCA 5.0.1 program [45]. These calculations were performed using a dispersion-corrected Becke–Johnson damping function PBE [46]-D3BJ [47] functional with the all-electron def2-SVP basis set [48]. The nature of all computed systems was verified using numerical vibrational frequency calculations. The effect of the water environment was simulated using the implicit universal solvation model based on density (SMD) [49]. Subsequently, the complexes between the crosslinked polyketone resins and the copper ions [XLPK-Cu(II)] adsorption conformations were found by Metropolis Monte Carlo search in the Adsorption Locator module of Materials Studio with the UFF method [50], where simulated annealing between 100–1.0 × 10^5^ K locates the global minimum XLPK-Cu(II) conformations in a large conformational space.

In order to estimate the stability and the interaction strength between the hydrated Cu^2+^ ion with the XLPKs, the adsorption energies (*E*_ads_) were calculated using the following equation:

*E*_ads_ = *E*_XLPK_ + *E*_Cu(II)_ − *E*_XLPK-Cu(II)_ (1)

where *E*_XLPK_, *E*_Cu(II)_*,* and *E*_XLPK-Cu(II)_ are the total energies of the free polyketones, free hydrated copper ion, and the XLPK-Cu(II) complex, respectively; thus, the more positive the *E*_ads_ values, the more stable the XLPK-Cu(II) complex is. Basis set superposition errors were corrected by the counterpoise method. Adsorption energies were decomposed into physical contributions by the ALMO-EDA(solv) scheme [51] that allows the application of continuum solvent models within the framework of energy decomposition analysis (EDA) based on absolutely localized molecular orbitals (ALMOs) in Q-Chem 5.4 [52]. Accordingly, *E*_ads_ is composed as a sum of stabilizing (∆*E*_ELEC_ + ∆*E*_DISP_ + ∆*E*_POL_ + ∆*E*_CT_), and destabilizing terms (∆*E*_PAULI_ + ∆*E*_SOLV_ + ∆*E*_PREP_). Here, ∆*E*_ELEC_, ∆*E*_DISP_, ∆*E*_POL_, and ∆*E*_CT_ stand for the energy lowering due to Coulombic electrostatic interactions, dispersion forces, intrafragment polarization energies, and interfragment charge transfer, respectively. ∆*E*_PAULI_ is the energy destabilization due to Pauli repulsion when two fragments are close in contact, ∆*E*_SOLV_ is the solvation energy, and ∆*E*_PREP_ is the energy required for realignment of the input electronic configuration of isolated fragments.

The feasibility of the forming XLPK-Cu(II) complexes was characterized by the complexation energy (∆*E*_com_), and the adsorption Gibbs free energy (∆G_ads_), enthalpy (∆H_ads_), and entropy (T∆S_ads_) changes as a function of typical temperature (271–311 K), and pressure (1–1000 atm) ranges in the seawater [53], which indicates how these thermodynamic state variables change at a given depth in the seawater.

Furthermore, the global reactivity of the isolated XLPKs and the hydrated copper ions within the conceptual DFT framework, in which the energy (E) can be treated as a function of the total number of electrons of the system (N) and the external potential [ν(r)]; E[N,ν(r)]. In this context, chemical potential (μ) and molecular hardness (η) are key quantities for global reactivity [54]; they are defined from the first (μ) and second partial (η) derivatives of the energy with respect to the number of electrons at a constant external potential. In this paper, we have formulated μ and η using Koopman’s theorem [55] in terms of the frontier molecular orbitals (FMOs) HOMO and LUMO (ε_H_ and ε_L_): μ= ½(ε_H_ + ε_L_) and η = −½(ε_H_ − ε_L_). μ characterizes the tendency of electrons to escape from the equilibrium distribution and is related to Mulliken’s electronegativity (χ) [56]. In contrast, η can be understood as a resistance to any change in the equilibrium electronic distribution. In addition, from μ and η, the electrophilicity index can be formulated as: ω = μ^2^/2η, which measures the stabilization energy when the system acquires additional electronic charge from the surroundings [57].

On the other hand, the difference in chemical potential (Δμ) and the donor–acceptor hardness (η_DA_) [58] were also analyzed among the reactive species of XLPKs and the hydrated copper ions. Δμ indicates the ability of a species to initiate a reaction through the electron transfer process, while η_DA_ characterizes the HOMO–LUMO gap between the donor and acceptor species (see more details in Section 3.2).

Noncovalent interactions in the XLPK-Cu(II) systems were analyzed using the Independent Gradient Model with the Hirschfeld partition (IGM) [59] in Multiwfn 3.8. The IGM describe qualitatively the intermolecular interaction regions: *δg*^inter^ = |∇*ρ*^IGM,inter^| − |∇*ρ*|, where ∇*ρ* stands for the electron density gradient, and ∇*ρ*^IGM,inter^ is an upper limit to ∇*ρ* regions by color codes; red, strong repulsions; blue, strong attraction; and green, weak interactions. Wavefunction analyses and CM5 atomic charges were obtained in Multiwfn 3.8 [60].

## 3. Results and Discussions

### 3.1. Properties of XLPK Systems

**Figure 1:**
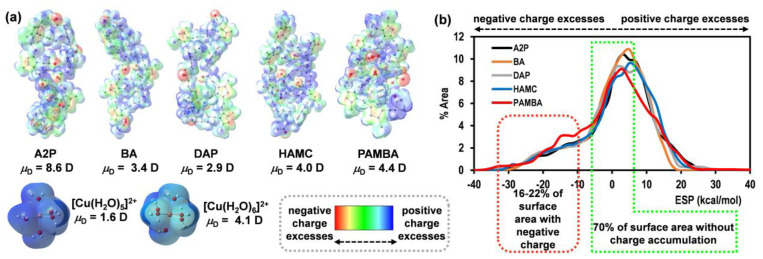
(**a**) Electrostatic surface potential (ESP) and dipole moment (*μ*_D_ in Debye, D) of free XLPKs and [Cu(H_2_O)_n_]^2+^ species. (**b**) Percentage of surface area in each ESP range (ESP in kcal/mol).

The sorption properties of the crosslinked polyketone resins and the copper ions [XLPK-Cu(II)] complexes will be based on their ability to interact with Cu^2+^ ions via strong driving forces, such as chemisorption or electrostatics. In this regard, the polarity of the crosslinked polyketone resins (XLPKs) plays a key role in the sorption behavior for the uptake of polar chemicals in solutions such as Cu^2+^. Previous DFT studies at the B3LYP level with CPCM solvation show that the most thermodynamically favored Cu(II) complexes are four (gas phase, [Cu(H_2_O)_4_]^2+^) or five-coordinate structure (aqueous phase, [Cu(H_2_O)_5_]^2+^) than the six-coordinate species with H_2_O molecules in square-pyramidal geometry [61]. This finding is also supported by neutron diffraction and molecular dynamic studies [62], revealing that [Cu(H_2_O)_5_]^2+^ species is favored instead of [Cu(H_2_O)_6_]^2+^ due to their 3d^9^ electronic structure, which causes a departure from octahedral coordination because of the Jahn–Teller effect. Our computations show that the [Cu(H_2_O)_5_]^2+^ and [Cu(H_2_O)_6_]^2+^ complexes display molecular dipole moments *µ*_D_ of 1.6 and 4.1 Debye, respectively (Figure 1a). Then, the Cu^2+^ uptake on adsorbents will be strengthened by dipole-dipole interactions with the adsorbent material. Considering the representative molecular fragments of the XLPK systems, their structures are associated with permanent dipoles in the range of *µ*_D_ = 2.9–8.6 Debye (Figure 1a), indicating that XLPKs could establish strong electrostatic interactions with [Cu(H_2_O)_5_]^2+^ in solution. The electrostatic surface potential displays the signature of the polar regions in the XLPK systems (ESP, Figure 1a), which show the charge distribution on the molecular surface area: blue and red colors stand for molecular regions with positive charges/ESP and negative charges/ESP. The ESP of XLPK systems displays regions accumulating negative charges, especially in the ketone-moieties and functional groups of the amino-derivatives because of the higher electronegativity of nitrogen and oxygen atoms compared to carbon and hydrogen (N (3.0), O (3.4) vs. H (2.2), C (2.6) in the Pauling scale). In fact, the negative charges in O and N atoms of polymers reach values of up to −0.4 and −0.6 *e*, respectively, which can interact with the positively charged sites of solvated Cu^2+^ ions.

In addition, Figure 1b shows the statistical ESP distribution on the whole surface area of the XLPK systems. First, all the polymers display similar ESP distributions, with a larger portion of the molecular surface with small ESP values (from (9 to +10 kcal/mol), which stand for ~70% of the surface area. In this case, the negative part of ESP is mainly associated with weakly negatively charged C atoms in the polymer chains (with atomic higher than −0.1 *e*), while the positive part emerges almost entirely from the low electronegative hydrogen atoms with average atomic charges of up ~0.1 *e*. Accordingly, the weak magnitude of positive/negative charges in ~70% of the molecular surface ensures that Cu^2+^ ions will be adsorbed on specific adsorption sites of XLPKs. Indeed, the surface sites with the lowest ESP values (from −30 to −10 kcal/mol) stand for ~16–22% of the whole surface area, i.e., the adsorption sites with the lowest negative charges are located in specific sites more than delocalized along the polymer surface. In this way, the negatively charged ketone moieties and functional groups of the amino derivatives turn into excellent adsorption sites for selective adsorption of Cu^2+^ ions.

### 3.2. Adsorption Energies

**Figure 2:**
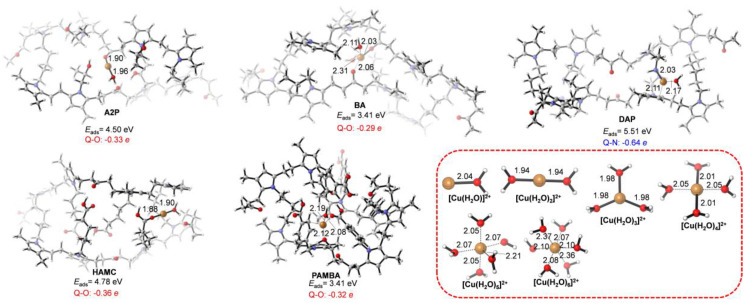
Adsorption conformers of XLPK−Cu(II) complexes. The adsorption energy (*E*_ads_) between XLPK and Cu^2+^ hydrated ions and the CM5 atomic charges of the binding site in the XLPKs is also represented. Cu in orange, N in blue, O in red, C in grey and H in white.

To explore the mode and the strength of the interaction between the crosslinked polyketone resins and the copper ions [XLPK-Cu(II)] complexes, we evaluated the adsorption energies (*E*_ads_) of the most stable conformers according to Figure 2. The copper ion was considered with surrounded water molecules reaching a hydration number of five, that is [Cu(H_2_O)_5_]^2+^, since it is the most stable species in the aqueous phase [61,62,63], as shown in Figure 2. Based on [Cu(H_2_O)_5_]^2+^, only two kinds of binding adsorption on the polyketones were found; they are monohydrated [Cu(H_2_O)]^2+^ and dihydrated [Cu(H_2_O)_2_]^2+^ copper ion. Figure 2 shows that the *E*_ads_ range of 3.41–5.51 eV corresponds to the adsorption of the [Cu(H_2_O)_2_]^2+^ with the BA and PAMBA polyketones in the lower limit, while the [Cu(H_2_O)]^2+^ uptake on the DAP polyketone is in the upper limit. Similarly to DAP, the A2P and HAMC polyketones also showed a monohydrated copper ion binding mode reaching adsorption energies of 4.50 and 4.78 eV, respectively. These results demonstrate that the less hydration number on Cu^2+^ involved in to uptake of the polyketones, the greater the adsorption energy. Therefore, the higher affinity for Cu^2+^ ion in the DAP, A2P, and HAMC polyketones is mainly associated with the increased electron density of the copper atom in its [Cu(H_2_O)]^2+^ form (1.36 *e*) vs. its [Cu(H_2_O)_2_]^2+^ form (1.08 *e*), which reduces its affinity with the BA and PAMBA polyketones. Based on our findings, it is evident that the A2P, DAP, and HAMC polyketones serve as exceptional adsorbents in an aqueous environment for binding Cu^2+^ ions. Among these, the DAP-Cu(II) complex exhibits the highest stability. These results highlight the promising potential of these polyketones for efficient Cu^2+^ ion adsorption. The maximum stability of the DAP-Cu(II) complex is probably due to the strongest binding mode of the nitrogen atom of the amino propane moiety (–NH_2_) in DAP (−0.64 *e*), which is in agreement with experimental high binding affinity towards Cu^2+^ ions, provided by the high reactivity and adsorption capacity (104.6 mg/g) of amine groups as binding site in poly(amidoamine) dendrimers [64]. On the contrary, when the Cu^2+^ ion was combined with oxygen atoms of the nearest ketone groups in the polymer main chain (A2P and BA) and the carbonyl group (–C=O) belonging to carboxylic acid functional groups (HAMC and PAMBA) as the binding mode (Figure 1), these systems reached atomic charges from −0.29 to −0.36 *e* with smaller *E*_ads_ than with DAP. Therefore, our results suggest that the XLPKs involve strong electrostatic interactions with the copper ion by the –NH_2_ and –C=O groups as the main adsorption mechanism. These results agree with experimental studies of the uptake of Cu^2+^ ions in poly arylidene ketone [65] and sulfonated polyketone [66], which resulted in a strong chemical bond by electrostatic contact between the copper ions and the chelating functionalities of the polymers.

**Figure 3:**
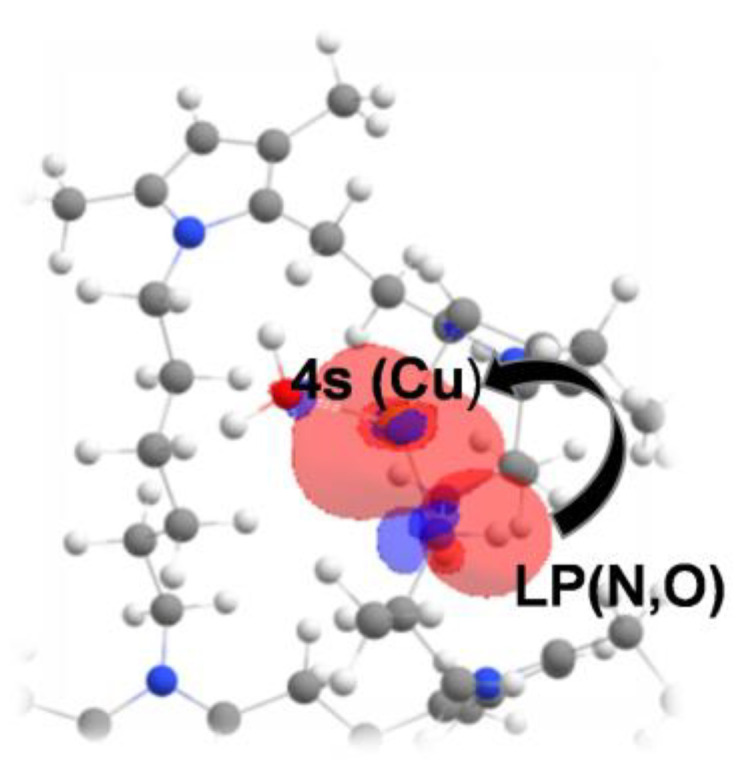
Natural bond orbitals associated with covalent bonding of XLPK-Cu(II) complexes. DAP-Cu(II) complex was taken as a representative case.

Furthermore, the short distances of the Cu^2+^ ion with the nitrogen or oxygen atoms in the XLPKs (Cu^2+^- X (N or O): 1.88–2.06 Å) suggests chemisorption in all the cases. This is supported by the Natural Bond Orbital (NBO) analysis (Figure 3), which indicates that in all the XLPK-Cu(II) complexes, the copper ion act as Lewis acid by the low-occupied 4s orbital, whereas the 2p lone pair electrons belonging to nitrogen and oxygen atoms from –NH_2_ (DAP) and –C=O (A2P, BA, HAMC, and, PAMBA) groups, respectively, act as the Lewis base. Thus the Cu(II)-N or Cu(II)-O interactions are coordinative covalent bonds in all the XLPK-Cu(II) complexes. Similar behavior has been found in related literature for the adsorption of Cu(II) on the Silica supported thiosemicarbazide (SG-GPTS-ATS) in an aqueous solution [63].

To further explore the reactivity of the chelating effect of each XLPK into Cu^2+^ ion, we also calculated the electronic chemical potential (μ) to measure the ability of charge transfer between the XLPKs and the hydrated Cu^2+^ ion (Table 1). Our results indicate that charge transfer will occur from the XLPKs to the copper ion since μ is greater for the polyketones than the hydrated copper ions (Table 1). In addition, a larger difference in chemical potential (Δμ) between each XLPK and the hydrated copper ions indicates a larger electron transfer between them and, thus, its chelating effect into Cu^2+^. In this sense, since μ is greater in [Cu(H_2_O)_5_]^2+^ than in [Cu(H_2_O)_6_]^2+^ species (Table 1), the charge transfer from XLPKs to the hydrated Cu^2+^ ion will be favored in the five-coordinate species. Furthermore, Table 1 shows that Δμ increase in the following order: PAMBA < BA < A2P < HAMC < DAP, in agreement with the adsorption energies, confirming that the monohydrated XLPK-Cu(II) complexes are strongly favored. This is also appreciated through the CM5 atomic charges on the copper ion, which decreases in these systems (Table 1). While in the case of the PAMBA- and BA-Cu(II) complexes show greater CM5 atomic charges (0.52 and 0.51 *e*, respectively) than the monohydrated HAMC- (0.50 *e*), A2P- (0.48 *e*) and DAP-Cu(II) (0.46 *e*) complexes. Interestingly, the donor–acceptor hardness (η_DA_) agrees with these results (Table 1), quantifying the affinity between the donor (XLPK) and the acceptor (Cu^2+^) species. Small values of Δη_DA_ indicate high reactivity to uptake Cu^2+^. It can be noted that Δη_DA_ values are considerably lower for A2P, DAP, and HAMC polyketones (Δη_DA_ ≈ 0.1 eV) than for BA and PAMBA (Δη_DA_ ≈ 0.9 eV).

To complement these results, we also analyzed the chemical hardness (η) to characterize the resistance of the XLPKs to change their electronic structure by uptaking the Cu^2+^ ion. The data in Table 1 indicate that the PAMBA polyketone is much more reactive than the other XLPKs since its hardness is considerably lower (η = 1.02 eV), suggesting it would be more prone to uptake the Cu^2+^. However, the electrophilicity (ω) values in Table 1 show that PAMBA is the more acidic polyketone with the highest value of ω (5.43 eV), making this polymer less adept at donating charge to the copper ion. While in the case of BA, this is the least electrophilic polyketone (ω = 3.33 eV), but at the same time, it is the least reactive (η = 1.32 eV), implying its lowest adsorption energy. In the case of the polyketones combined with the monohydrated copper ion, these present a moderate reactivity and electrophilicity, where DAP (η = 1.29 eV) is less reactive than A2P (η = 1.18 eV) and HAMC (η = 1.19 eV), but in turn is less electrophilic (ω = 3.59 eV). Our results suggest that a lower electrophilicity of the XLPK would be associated with higher adsorption energy.

### 3.3. Adsorption Mechanism

**Figure 4:**
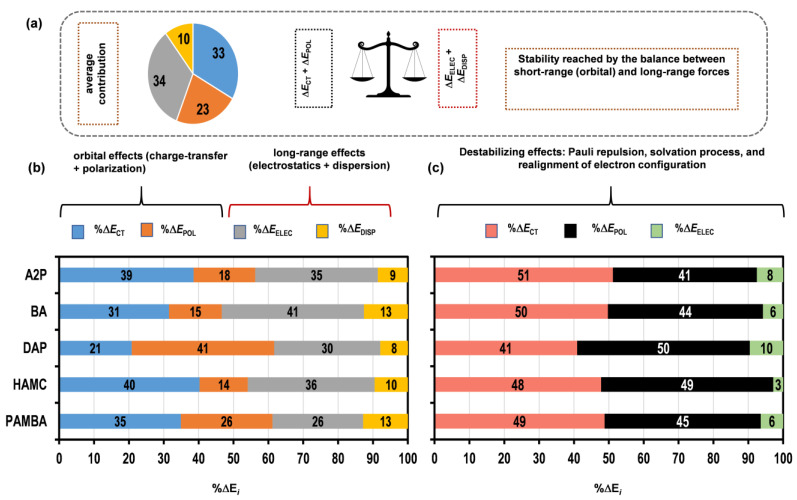
ALMO-EDA results: (**a**) Average contribution of stabilizing terms; the relative single percentage contributions (%∆*E*_i_, in %) of stabilizing (**b**) and destabilizing (**c**) EDA terms in the XLPK-Cu(II) complexes in water.

The *E*_ads_ values were quantitatively decomposed into specific physical stabilizing contributions (∆*E*_ELEC_, ∆*E*_DISP_, ∆*E*_POL_, ∆*E*_CT_) and destabilizing contributions (∆*E*_PAULI_ + ∆*E*_SOLV_ + ∆*E*_PREP_) by the ALMO-EDA(solv) method to understand the adsorption mechanism between the crosslinked polyketone resins and the copper ions in the formed XLPK-Cu(II) complexes. Figure 4 shows the relative single percentage contributions (%∆*E*_i_) terms for a normalized comparison among systems.

The strong stability of the XLPK-Cu(II) complexes emerges from a balance between short-range effects (responsible for chemisorption, ∆*E*_POL_, and ∆*E*_CT_) and long-range effects (responsible for pairwise interactions, ∆*E*_ELEC_ and ∆*E*_DISP_). In general, the combined contribution of charge-transfer and polarization effects (∆*E*_POL_ + ∆*E*_CT_) accounts for ~56% of the stabilizing energy, denoting the key role of orbital interactions by coordinative covalent bonding between Cu(II) and XLPKs (Figure 4a), and as indicated above from NBO analyses (Figure 3). In this regard, the charge transfer term ∆*E*_CT_ account for donor–acceptor inter-fragment orbital interactions, allowing the charge transfer between fragments. In addition, the polarization term (∆*E*_POL_) stands for the on-fragment relaxation of each species to the presence of the nuclei and electrons of all other fragments, then inducing density rearrangements that result in energy lowering. In this way, ∆*E*_CT_ and ∆*E*_POL_ become significant in the regime of the orbital interactions such as coordinative covalent bonding [67]. Otherwise, the long-range effects are dominated by Coulombic electrostatic attractions, which stand for ~34% of the stabilizing energy. Dispersion effects play a minor role in the stability (~10%) compared to the magnitude of charge transfer, polarization, and electrostatic driving forces. Then, the adsorption mechanism of the XLPK-Cu(II) complexes is explained by the interplay between charge transfer, polarization, and electrostatics driving forces. The same behavior has been found in related literature [65,66,68] for other polymers, in which the Cu^2+^ uptake in aqueous environment resulted in a strong chemical bond by electrostatic interactions with the chelating functionalities of the polymers with a contribution through van der Waals forces.

The analysis of the single percentage contributions explains the relative differences in stability among systems (Figure 4b). The DAP-Cu(II) complex reaches the highest stability due to the strong contribution from polarization effects (41%) in the bonding, i.e., the energy lowering gained by polarization relaxation is the highest among all the systems, reaching a strong coordinative covalent binding. For instance, the |∆*E*_POL_| term stands for 3.8 and 1.2 eV of the stabilizing energy in the DAP-Cu(II) and HAMC-Cu(II) complexes, respectively. At this point, it is important to note that polarization effects occur in the form of density rearrangements, which create induced multipole moments in the fragments; thus, polarizability determines the ability to develop these induced moments. With this in mind, nitrogen atoms display higher atomic polarizability than oxygen atoms [69]; therefore, the amino group (–NH_2_) in DAP induces higher fluctuations in the Cu^2+^ electron density, resulting in the on-fragment relaxation that reduces the kinetic energy pressure exerted by the electrons in the fragments as a result of a stronger covalent binding. Accordingly, Wang and coworkers [70] found similar adsorption properties predicted by DFT calculations, where poly(2-acrylamide-pentanedihydroxamic acid) (PAPDA) resin containing –CONHOH and –COOH groups showed that –CONHOH group has a greater affinity to uptake Cu^2+^ and Ni^2+^ ions by amine N atom in water increasing its adsorption capacity by a factor of 10 units.

On the other hand, the A2P-Cu(II) (*E*_ads_ = 4.50 eV) and HAMC-Cu(II) (*E*_ads_ = 4.78 eV) complexes show higher adsorption energies as a result, the higher contributions from charge-transfer effects in the bonding, with an ∆*E*_(CT)_ contribution of 39–40% of the whole stabilizing energy. Therefore, the Cu(II)-O binding mode does not increase the polarization effects like the Cu(II)-N binding in DAP-Cu(II) complex but favors the electron movement between fragments, which could be explained by the higher electronegativity of oxygen atoms compared to nitrogen [N (3.0) vs. O (3.4), in the Pauling scale]. As an illustration, the |∆*E*_CT_| term stands for 3.3 and 3.1 eV of the stabilizing energy in the A2P-Cu(II) and HAMC-Cu(II) complexes, but it corresponds to only 2.0 eV in the DAP-Cu(II) complex.

Additionally, the destabilizing effects emerge mainly from the Pauli repulsion (∆*E*_PAULI_) and solvation effects (∆*E*_SOLV_), which stand for at least 90% of the destabilizing energies (Figure 4c). Despite the latter, the attractive forces are high enough to overcompensate the destabilizing effects, in agreement with the positive adsorption energies for all the complexes (i.e., |∆*E*_ELEC_ + ∆*E*_DISP_ + ∆*E*_POL_ + ∆*E*_CT_| > ∆*E*_PAULI_ + ∆*E*_SOLV_ + ∆*E*_PREP_. Furthermore, the solvation energies (∆*E*_SOLV_) are positive in all the cases, indicating that energy is required to allow the solvation process. Because the electrostatic term ∆*E*_ELEC_ is described by a classical Coulombic potential, it must be inversely proportional to the solvent dielectric constant (*ε*) in aqueous environments. Then, the ∆*E*_ELEC_ term tracks the energy penalty due to the solvation process. The ∆*E*_ELEC_ terms are always below 0 (∆*E*_ELEC_ < 0)_,_ denoting that the strong magnitude of electrostatic attraction hinders the destabilization caused by the solvation process. Finally, a little portion of the destabilizing energy is associated with the prearrangement of the unpaired electrons in Cu^2+^ to form the bonded state with the polymers (∆*E*_PREP_, 3–10%).

**Figure 5:**
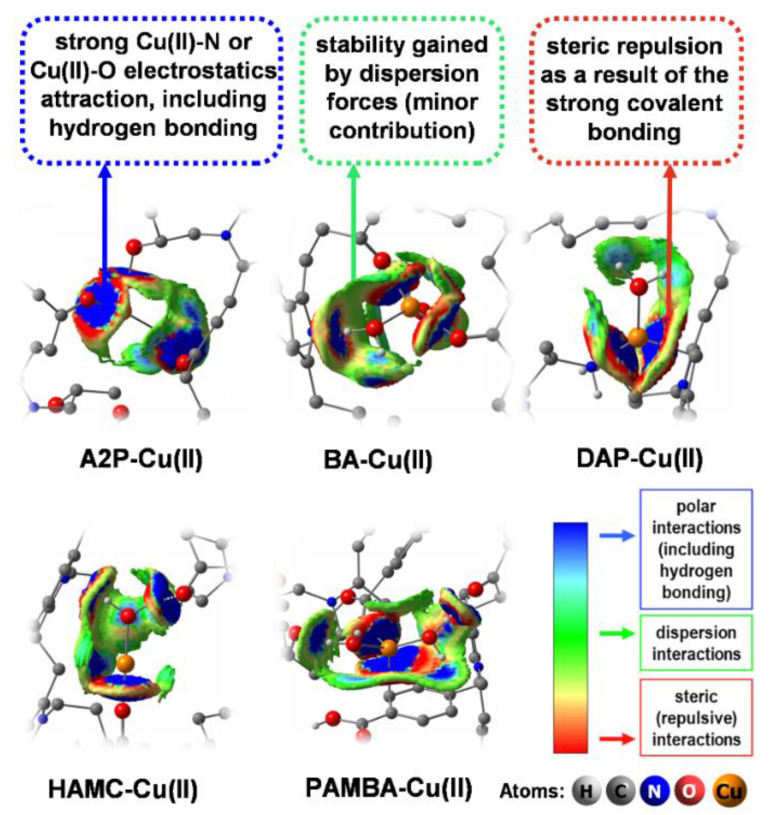
Independent gradient model (IGM) analysis of weak intermolecular interactions.

From the chemical viewpoint, the IGMH analysis reveals the signature of the intermolecular interactions in the XLPK-Cu(II) complexes (Figure 5). The blue regions can be associated with strong electrostatic attractions, but at shorter interatomic distances, these regions are related to the existence of chemical bonding in the Cu(II)-N or Cu(II)-O mode (Figure 3). The latter is also supported by red regions in the IGM pattern because of steric repulsion that is always associated with chemical bonding due to Pauli repulsion, in agreement with the key role of the ∆*E*_PAULI_ term in the destabilizing energies as noted above. The water molecules bonded to Cu^2+^ ion also display polar attractions via hydrogen bonding with the polymer, denoting that water molecules assist the bonding in the form of extra stabilization gained by electrostatic effects. These results demonstrate that the less hydration number on Cu^2+^ involved in the uptake of the polyketones, the greater the adsorption energy. Otherwise, dispersion forces envelop the Cu^2+^ species in the interacting regions (green surfaces), providing extra bonding stabilization. Although dispersion effects play a relatively minor role in the interaction mechanism, the ALMO-EDA analysis indicates that dispersion energies can reach magnitudes of up to ∆*E*_DISP_ = 1.0 eV, which are not negligible compared to non-covalent interactions.

### 3.4. Thermochemistry

To evaluate the feasibility of the adsorption reaction forming of the crosslinked polyketone resins and the copper ions XLPK-Cu(II) complexes, we have studied their complexation energy (∆*E*_com_) and thermochemistry in an aqueous environment by analyzing the changes in the Gibbs free energy (∆G_ads_), enthalpy (∆H_ads_), and entropy (T∆S_ads_) according to the following reaction:

[Cu(H_2_O)_5_]^2+^ + XLPK → [XLPK-Cu(H_2_O)_n_]^2+^ + nH_2_O

where “n” in the [XLPK-Cu(H_2_O)_n_]^2+^ complex is the number of water molecules that remain bound to the copper ion when it is adsorbed by the XLPKs, whereas “n” in nH_2_O corresponds to the water molecules released after the hydrated metal ion is adsorbed. The complexation energy and the adsorption Gibbs free energies at 298 K are summarized in Table S1; both parameters are representative of the complexing ability of XLPKs to Cu^2+^ uptake. Our results indicate a complexation ability in the following order: DAP-Cu(II) > BA-Cu(II) ≈ PAMBA-Cu(II) > HAMC-Cu(II) > A2P-Cu(II), which confirms that the amino nitrogen atom in DAP-Cu(II) is more stable coordination than ketone oxygen atoms in the rest of the polyketones. Regarding BA- and PAMBA-Cu(II) complexes, these have lower complexation energy than A2P- and HAMC-Cu(II) complexes, which is probably associated with the formation of bidentate vs. monodentate complexes, respectively, as is appreciated in Figure 2. While the adsorption energies are stronger in A2P- and HAMC-Cu(II) complexes than in A2P- and HAMC-Cu(II) complexes since the Cu-O bonds are too much shorter (Figure 2). The adsorption Gibbs free energies describe a spontaneous nature of the complexation reaction in all the formed XLPK-Cu(II) complexes, being BA polyketone which presents a better complexing ability for Cu(II) followed by DAP, PAMBA, HAMC, and A2P. Therefore, the different trends in ∆*E*_com_ and ∆G_ads_ indicate that the complexation stability depends on the coordinator atom (N or O) and the coordinating environment, in which this latter entropic contribution may also be involved in the XLPK-Cu(II) complex formation.

**Figure 6:**
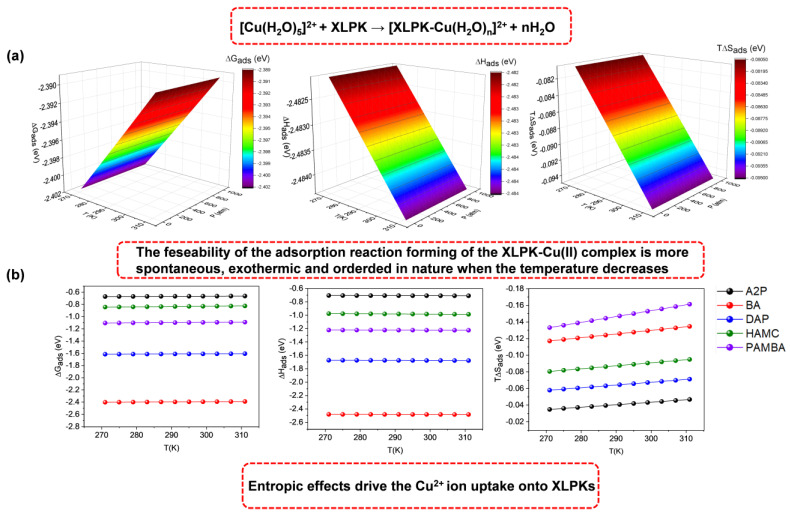
(**a**) Adsorption free energy (∆G_ads_), enthalpy (∆H_ads_), and entropy (T∆S_ads_) changes for the XLPK-Cu(II) forming at typical temperature (271–311 K), and pressure (1–1000 atm) ranges in the seawater. BA-Cu(II) system was taken as a representative case. (**b**) ∆G_ads_, ∆H_ads_, and T∆S_ads_ for XLPK-Cu(II) systems with respect to the temperature (271–311 K) at 1 atm.

On the other hand, to give more insights into the chelating interactions of the forming XLPK-Cu(II), the effect of temperature (271–311 K) and pressure (1–1000 atm) were also analyzed for thermochemical function states according to their typical range values in the seawater [53]. Figure 6a shows that the Cu^2+^ ion adsorption process on XLPKs is spontaneous (∆G_ads_ < 0), exothermic (∆H_ads_ < 0), and slightly ordered (T∆S_ads_ < 0) in nature for all the ranges of temperatures and pressures studied. Observe that for exothermic and ordered-in-nature reactions, ∆G_ads_ is more spontaneous at lower temperatures. This result suggests that the uptake of the hydrated Cu^2+^ ions by XLPKs would be favorable at a greater depth in the seawater. Specifically, the adsorption reaction forming of the XLPK-Cu(II) complexes is more feasible when the temperature decreases and the pressure increases, leading to higher adsorption of Cu^2+^ ions under these conditions. A similar trend is observed for a similar functionalized polymer with –NH and –OH groups, which complexed the hydrated Cu^2+^ ion by a spontaneous, exothermic, and ordered process where the removal percentage decreases as the temperature rises [71,72].

Figure 6b displays the behavior of ∆G_ads_, ∆H_ads,_ and T∆S_ads_ for the formation of each XLPK-Cu(II) complex with respect to the temperature at 1 atm. Although the thermodynamic adsorption trend is the same for each XLPK-Cu(II) forming system (Figure 6a), this is more exergonic in the following order: BA > DAP > PAMBA > HAMC > A2P, which differs from *E*_ads_ stability. Our calculated entropy values (T∆S_ads_) reveal that the adsorption of the monohydrated Cu^2+^ ion is more disordered in the order of A2P-Cu(II) > DAP-Cu(II) > HAMC-Cu(II) systems than in the dihydrated Cu^2+^ ion to form BA-Cu(II) and PAMBA-Cu(II) systems as it contains less negative entropy values. Hence, the higher entropic effects in the A2P, DAP, and HAMC polyketones explain the lower exergonicity of the adsorption reaction forming for the XLPK-Cu(II) system instead of the BA and PAMBA polyketone. This is probably due to releasing four water molecules from the hydration shell forming A2P-, DAP-, and HAMC-Cu(II) complexes instead of three for BA- and PAMBA-Cu(II) complexes. Most interestingly, our results agree with the experimental study of the thermodynamic adsorption process of hydrated metal ions such as Cu^2+^, Cd^2+^, Ni^2+^, and Co^2+^ onto porous resin of poly(methyl vinyl ether-*alt*-maleic anhydride), in which the authors found that whereas a greater amount of water molecules were released, the greater the entropy reached [72]. Thus, we can conclude that entropic effects drive the Cu^2+^ ion uptake onto XLPKs.

## 4. Conclusions

We investigated the reactivity of functionalized and crosslinked polyketones (XLPKs) for Cu^2+^ uptake in water treatment applications using DFT calculations. The objective was to gain valuable insights into the adsorption mechanisms and factors influencing the interaction between XLPKs and Cu^2+^ ions. Our computational analysis revealed that dipole-dipole interactions played a crucial role in enhancing the adsorption of Cu^2+^ ions onto XLPK adsorbents. The statistical distribution of electrostatic potential highlighted the negatively charged ketone moieties and functional groups in XLPKs as prominent adsorption sites for the selective binding of Cu^2+^ ions.

Furthermore, we evaluated the adsorption energies of the XLPK-Cu(II) complexes and found that the DAP-Cu(II) system exhibited the highest stability. This was attributed to the strong interaction energy between the amino moiety (–NH_2_) in DAP and the Cu^2+^ ion (Cu(II)-N). In contrast, other XLPKs showed binding modes through oxygen atoms (Cu(II)-O) of the ketone moieties in the main chain. The polarizability of XLPKs played a crucial role in electron density fluctuations, with nitrogen atoms inducing stronger covalent binding and higher stability than oxygen atoms. Our results indicated that XLPKs engage in strong electrostatic interactions with Cu^2+^ ions primarily through the –NH_2_ and –C=O groups, serving as the main adsorption mechanism. Additionally, a lower hydration number of the Cu^2+^ ion led to stronger adsorption and the formation of more stable complexes. Among the tested polyketones, A2P-, DAP-, and HAMC-Cu(II) complexes exhibited great potential as adsorbents, forming monohydrated Cu^2+^ complexes [Cu(H_2_O)]^2+^. However, BA- and PAMBA-Cu(II) complexes displayed lower adsorption stability due to higher Pauli repulsion destabilization.

The complexation and thermochemical analysis emphasized the role of the coordinator atom (N or O) and the coordinating environment, in which higher entropic effects involved in the adsorption of Cu^2+^ ions onto XLPKs describes a lower spontaneity of the adsorption process. The adsorption reactions were favored at lower temperatures and higher pressures.

These findings provide crucial insights for designing high-performance polymeric materials for water treatment. Understanding the adsorption mechanisms and factors influencing the reactivity of functionalized and crosslinked polyketones toward Cu^2+^ ions can inform the development of efficient and sustainable water treatment processes. Experimental studies can further validate and leverage these computational insights to enhance the performance of water treatment systems and address the urgent challenge of copper contamination in water resources.

## Figures and Tables

**Table 1:** Reactivity indexes [chemical potential (μ), difference in chemical potential (Δμ), chemical hardness (η), donor–acceptor hardness (η_DA_), and electrophilicity (ω)] for the isolated XLPKs, the monohydrated (*n* = 1), dihydrated (*n* = 2) and tetrahydrate (*n* = 4) copper ions [Cu(H_2_O)_n_]^2+^. The CM5 atomic charge on the Cu^2+^ ion is also represented for the isolated hydrated copper ions and the XLPK-Cu(II) complexes.

System	μ (eV)/|Δμ| ^a^ (eV)	η(eV)/η_DA_^b^ (eV)	Ω (eV)	QCu^2+^|*e*|
**A2P**	−3.16/3.90 ^a^	1.18/0.08 ^b^	4.23	0.48 ^c^
**BA**	−2.96/2.81 ^a^	1.32/0.84 ^b^	3.33	0.51 ^c^
**DAP**	−3.04/4.01 ^a^	1.29/0.08 ^b^	3.59	0.46 ^c^
**HAMC**	−3.12/3.94 ^a^	1.19/0.07 ^b^	4.08	0.50 ^c^
**PAMBA**	−3.33/2.44 ^a^	1.02/0.88 ^b^	5.43	0.52 ^c^
**[Cu(H_2_O)]^2+^**	−7.05	2.88	5.23	1.36
**[Cu(H_2_O)_2_]^2+^**	−5.77	3.18	8.63	1.08
**[Cu(H_2_O)_5_]^2+^**	−4.20	3.09	2.85	0.88
**[Cu(H_2_O)_6_]^2+^**	−3.72	2.57	2.68	0.82

^a^ Difference in chemical potential, Δμ = (μXLPK − μ[Cu(H_2_O)_n_]^2+^). ^b^ Donor–Acceptor hardness (η_DA_) between the molecular orbital values of the [Cu(H_2_O)_n_]^2+^ (acceptor) and XLPK (donor), η_DA_ = ½(ɛ_LUMO_[Cu(H_2_O)_n_]^2+^− ɛ_HOMO_ XLPK). ^c^ CM5 atomic charge on Cu^2+^ ion in the XLPK-Cu(II) systems.

## Data Availability

Cartesian coordinates of the optimized structures can be found in the Supplementary Material.

## Supplementary Materials

The following supporting information can be downloaded at: https://www.mdpi.com/article/10.3390/polym15153157/s1, Table S1: Calculated complexation energies and adsorption Gibbs free energies at 298K (in eV); Cartesian coordinates of the optimized structures.
