# Supplementary material for: Computational Insights on the Chemical Reactivity of Functionalized and Crosslinked Polyketones to Cu2+ Ion for Wastewater Treatment
Source: Polymers (Basel). 2023 Jul 25;15(15):3157. doi: 10.3390/polym15153157 (PMC10420987; doi:10.3390/polym15153157)
Supplement: Supplementary file 1 [file polymers-15-03157-s001.zip › polymers-2521544-supplementary.pdf]

Supporting Information for

Computational Insights on the Chemical Reactivity of Functionalized and Crosslinked Polyketones to Cu^{2+} Ion for Wastewater Treatment

Daniela E. Ortega [1,*], Diego Cortés-Arriagada [2] and Rodrigo Araya-Hermosilla [2]

[1] Centro Integrativo de Biología y Química Aplicada (CIBQA), Facultad de Salud, Universidad Bernardo O'Higgins, General Gana 1702, Santiago 8370854, Chile
[2] Programa Institucional de Fomento a la Investigación, Desarrollo e Innovación (PIDi), Universidad
Tecnológica Metropolitana, Ignacio Valdivieso 2409, San Joaquín, Santiago 8940577, Chile;
dcortes@utem.cl (D.C.-A.); rodrigo.araya@utem.cl (R.A.-H.)
* Correspondence: daniela.ortega@ubo.cl

Table S1. Calculated complexation energies and adsorption Gibbs free energies at 298K (in eV)

Complex	ΔE_{com}	ΔG_{ads}
	**$[Cu(H_2O)_5]^{2+}$**	
**A2P-Cu(II)**	-0.69	-0.67
**BA-Cu(II)**	-1.18	-2.39
**DAP-Cu(II)**	-1.70	-1.61
**HAMC-Cu(II)**	-0.97	-0.83
**PAMBA-Cu(II)**	-1.19	-1.09

Cartesian coordinates of the optimized structures

A2P

C	8.062926838881	3.486328781777	1.230438434728
C	9.285023691926	4.161992837444	1.111074422960
C	9.972396047149	4.005518602416	2.358938113840
C	9.172322457761	3.246104992982	3.212435649398
N	8.001902900150	2.935194745003	2.517435259652
H	10.957019762163	4.423580645538	2.620548180852
C	9.430340668478	2.834276306658	4.632895740022
H	9.510891713380	1.730319820453	4.771950635933
H	10.391854144208	3.274974412862	4.971537195540
H	8.641474481461	3.194746293450	5.335831787902
C	7.006497265783	1.953174879131	2.951803737287
H	7.057968422969	1.878534909217	4.060844092650
H	5.985053030905	2.330597661536	2.714422807009
C	7.192526216856	0.559350859459	2.308180533047
H	6.350192392631	-0.092019696051	2.645635917904
H	7.061950712610	0.667644837255	1.206939532110

C	8.542459147367	-0.111119523084	2.619281700771
H	9.367205702816	0.611522947019	2.409301574167
H	8.596716813792	-0.330391633913	3.712898285048
C	8.821374735565	-1.409530180516	1.835189318412
H	9.740411648368	-1.884070485269	2.255895429544
H	7.998268471458	-2.146297999092	2.011537273457
C	9.019655608450	-1.211045622286	0.320264992587
H	8.065869725738	-0.894317753490	-0.165332042951
H	9.736612180333	-0.373422857526	0.142036150273
C	9.538970500285	-2.484461039286	-0.376910509727
H	8.865715685874	-3.342781318685	-0.153077219281
H	10.533623366857	-2.779310610855	0.024784779471
C	6.904817374430	3.464694750970	0.269291665945
H	6.172477161098	2.670006364539	0.532869837911
H	7.261543533351	3.205744064343	-0.756141602314
C	6.157143166962	4.836449719019	0.192159311172
H	5.936143536910	5.194475017041	1.222576421828
H	6.851125770856	5.592631779020	-0.238715388834
C	1.287231173217	4.059141179118	-1.019655691728
H	0.895510460076	4.402515063502	-0.035770772555
H	0.674356396875	4.586932303244	-1.786457908385
C	1.008589709632	2.530300745754	-1.133838399304
H	1.721099969324	1.980506443510	-0.469768938179
H	1.206540942605	2.167160288078	-2.167504834186
C	-0.391046472296	2.150186325522	-0.672268694055
C	-1.230206105753	1.249214283291	-1.565854501253
H	-2.013659337320	0.755032160049	-0.952898804113
H	-0.601318740487	0.460117402989	-2.037785184749
C	-1.922720686593	2.085228384547	-2.702204923626
H	-2.403337900649	2.973718866795	-2.237179015903
H	-1.142786766916	2.483320872992	-3.390647040210
O	-0.840704211690	2.621170652372	0.381006540063
C	-6.087443495207	-0.657965574232	-3.701216372623
H	-5.910680623483	-1.759622797992	-3.752094880109
H	-6.421075585381	-0.472097237090	-2.658721087410
C	-7.259730109066	-0.333600290333	-4.675897305178
H	-6.931092728585	-0.560559388149	-5.714697389259
H	-7.464183603109	0.760597086962	-4.652422690430
C	-8.525024880863	-1.112780110636	-4.432267146887
C	-9.017570831851	-2.199878480028	-5.155978907045
N	-9.457557544910	-0.822071569836	-3.430632194473
C	-10.282417988284	-2.578602789374	-4.605317257198
H	-8.515301716611	-2.661096415461	-6.020812296594
C	-10.541028660129	-1.703672917682	-3.540501313574
C	-11.743369772299	-1.674544200120	-2.633685631877
H	-12.664962976081	-1.787332574543	-3.252408519059
H	-11.853248993039	-0.686401970927	-2.134932803751
C	-11.749974921072	-2.776113673091	-1.543268285173
H	-12.134372122296	-3.746174771212	-1.937386035442
H	-10.700143226297	-3.009781027930	-1.230921861799
C	-12.478339520103	-2.410724426292	-0.243165734505
C	-12.867348430778	-3.577832341652	0.658284626915
H	-13.177593872447	-3.210025337971	1.658290393716
H	-13.716407970456	-4.136079500818	0.198522574483
H	-12.031266408263	-4.306927221661	0.760019703752
O	-12.698100164398	-1.247897248608	0.077489046208

N	9.652960803903	-2.355785048157	-1.831095138545
C	10.760893677803	-1.835024988133	-2.507021557068
C	8.632702155745	-2.620764444401	-2.744677796780
C	10.444203224240	-1.781048587178	-3.873547093442
C	9.110288181182	-2.278425483684	-4.012123322665
H	8.550847346273	-2.376509810213	-4.955555421180
C	12.051553898093	-1.424903440246	-1.841598224013
H	12.340551363770	-0.414317612397	-2.216817052289
H	11.924737577121	-1.307917702698	-0.743013165193
C	13.238385359585	-2.379269381294	-2.108184442960
H	13.368546570949	-2.560390916328	-3.200575522745
H	14.196628814370	-1.897839901788	-1.781647792104
C	13.186017033281	-3.720423069569	-1.368210109615
C	14.009278760721	-4.850037801455	-1.976662643262
H	14.100032014742	-5.694655391477	-1.263105195043
H	13.497223617640	-5.211072092980	-2.899818180383
H	15.018593227209	-4.500747725331	-2.291963222362
O	12.540982843306	-3.879440490451	-0.338445361704
C	7.280492985718	-3.164282224709	-2.361829779701
H	7.170975145919	-3.201990719550	-1.255137405765
H	7.167594609761	-4.223879163420	-2.698560534093
C	6.092406922804	-2.339315920291	-2.943985034828
H	6.257701268506	-1.264254144275	-2.709547921176
H	6.118424972411	-2.401080904587	-4.055661237473
C	4.749288420213	-2.819192674140	-2.466740742118
C	3.947123638265	-3.842847787310	-2.994786616286
N	4.180479167493	-2.410640584707	-1.255371375747
C	2.870172075218	-4.048816000060	-2.074285463808
C	3.028048380705	-3.164931499709	-1.006057339149
H	2.059114959634	-4.787490829741	-2.171645169741
C	4.635347789629	-1.302275022031	-0.420927480559
H	4.427129971806	-1.554503798031	0.642646560020
H	5.741840187260	-1.210621785362	-0.496495056503
C	2.178332611653	-2.987561531904	0.219938359221
H	1.744026779713	-3.975445385152	0.491704037684
H	2.819052924674	-2.696287858091	1.084634625278
C	1.003981871511	-1.946846310482	0.087650750820
H	1.282262243103	-1.176670963899	-0.665953773698
H	0.090237490010	-2.455458618272	-0.291148767504
C	0.735805329158	-1.214823817364	1.401978463325
C	-0.494334843155	-1.627091467763	2.193602211990
C	-0.775636408861	-0.808877024224	3.473806191789
H	-0.697486958035	0.274770951619	3.229725902259
H	0.050394300412	-0.994265722845	4.199123533221
C	-2.095544226312	-1.137197728234	4.121959751876
C	-2.423850028476	-2.243679426268	4.915581086515
N	-3.253167265709	-0.361207896118	3.968537124913
C	-3.814343103749	-2.127030094389	5.241589179195
C	-4.312445866302	-0.963687310503	4.651534847697
H	-4.388755733962	-2.826495002640	5.866716297683
C	-3.346517642624	0.873402144588	3.195073095418
H	-2.477018640319	1.529417163567	3.433485906086
H	-4.251470713204	1.435599267024	3.513568327327
H	-1.367399966603	-1.602771246124	1.495707896055
H	-0.385706735794	-2.716779570788	2.427941688514
O	1.510016594082	-0.345117130044	1.814432880152

C	-5.696224800882	-0.366413199499	4.714051978057
H	-5.686279544492	0.608358489201	5.263378946437
H	-6.061771041714	-0.115030166699	3.690657446858
C	-6.737807853696	-1.294118671536	5.390851505330
H	-6.422087323867	-1.472026922186	6.445325209066
H	-6.696903476392	-2.295853918009	4.902063102203
C	-8.145633430348	-0.762555067945	5.405942646586
C	-8.751569615006	0.079040635033	6.349580913829
N	-9.105087076191	-1.065240505073	4.428994327627
C	-10.105344221210	0.279213432036	5.924473416780
C	-10.308161300601	-0.433116949917	4.743000903234
H	-10.872635450160	0.873510007885	6.444597988845
C	-11.565256900874	-0.615927114773	3.942556141941
H	-11.937264098020	-1.669966283517	3.963556368811
H	-12.370550050331	0.016540884421	4.371947475545
H	-11.470427053434	-0.336305269016	2.866991739169
C	-8.891036688679	-1.852473152989	3.219905968363
H	-8.069623676420	-2.580962635291	3.406949230080
H	-9.803204703989	-2.467128550446	3.032570903922
C	-8.575407392293	-1.014576117609	1.962294364721
H	-9.387334480497	-0.264401608477	1.820537742322
H	-7.642477383965	-0.427353895985	2.133123509225
C	-8.428762224513	-1.887278312885	0.704834453689
H	-9.354077826186	-2.505591635858	0.589948787937
H	-7.601222411132	-2.621912026833	0.858971310349
C	-8.178004408226	-1.124267127494	-0.613225779877
H	-8.077212708468	-1.875310547209	-1.431943448690
H	-7.190703245968	-0.605412582584	-0.554118463908
C	-9.282484837621	-0.113362201195	-0.984015140595
H	-10.271082427366	-0.509297833810	-0.659504176899
H	-9.155554015848	0.831868747873	-0.405026613080
C	-9.360747318737	0.290743051968	-2.477013382245
H	-10.240395410715	0.964731476985	-2.613663438307
H	-8.475802689659	0.901304745271	-2.759642878316
C	-3.406730086743	0.682785876238	1.650466771909
H	-2.457798094275	0.175440089569	1.335598760662
C	3.956804268351	0.079628341199	-0.725142691548
H	2.934936436069	-0.148236076991	-1.130780584899
C	4.695810110324	0.904354908117	-1.780854822457
H	4.202700097472	1.890305091032	-1.929212611713
H	4.707678633000	0.373228058477	-2.757401834440
C	4.908914009918	4.815578079881	-0.649025498736
C	2.725466571575	4.443221241623	-1.209908510560
C	3.398736207219	4.817491584005	-2.382752496737
H	5.576631395547	5.362555526426	-2.694096242224
C	4.764598639059	5.051235342202	-2.018732335709
N	3.655604758617	4.428745310449	-0.164691579250
C	3.328397396477	4.199890374146	1.241544561189
H	4.229290046694	3.797937159787	1.754864604125
H	2.553464433287	3.405561413213	1.314760780038
C	2.843944768044	5.478097212744	1.977940612511
H	1.881037005996	5.805309197337	1.496035633348
C	2.570370055029	5.187239926824	3.455139571976
H	2.232456686976	6.110291837025	3.973543687974
H	1.780589545020	4.412513889908	3.570901956874
C	-2.900243370159	1.274755467226	-3.506895328593

C	-4.771789524768	0.037917702359	-3.951378617885
C	-3.813938344185	-0.274417792079	-4.931125755466
H	-1.695915450828	0.477219314521	-5.194423730241
C	-2.649530131635	0.503089903764	-4.643761341877
N	-4.206432104704	0.996125293265	-3.090764434443
C	-4.930616602135	1.718462680736	-2.040756433845
H	-5.704436774385	1.035574066286	-1.628545876118
H	-4.253744423804	1.945630787859	-1.186808935201
C	-5.652825185972	3.014916288827	-2.510660037717
H	-6.260695545206	3.345757697648	-1.633213044786
C	-4.723656588997	4.170948472097	-2.911767586644
H	-4.065643910969	3.889823203029	-3.766581052928
H	-5.332472810850	5.047064929297	-3.223976926627
H	-4.075530862219	4.489199881226	-2.064353905983
H	3.493887129634	4.828954278896	3.964185153996
H	5.749516481323	1.097455936927	-1.477665230654
C	-4.581944657821	-0.187735492882	1.195630653358
H	-5.540289625966	0.360270031752	1.339385174474
H	-4.494334498251	-0.441000089419	0.116326984540
H	-4.628699252809	-1.139677184301	1.768667152309
O	-6.607188154919	2.748176888999	-3.544156263539
H	-6.102339545947	2.432357881050	-4.326241454158
O	3.807275994211	6.529793210877	1.912528429054
H	3.979696256733	6.711019628229	0.963009171725
O	3.854792416352	0.854683978043	0.472339161828
H	3.148114654542	0.439310564362	1.030047996061
O	-3.503969035614	1.964429637963	1.031233817796
H	-2.578948396839	2.308324628081	0.904334961438
C	9.764952981323	4.944624508254	-0.082947213675
H	9.301112114726	4.589376411170	-1.030741899964
H	9.537740531231	6.034920072533	0.000264277471
H	10.868396132989	4.859183226219	-0.204572836328
C	2.811224852043	4.934958143572	-3.764299759304
H	3.032592121167	4.041352232576	-4.395783911181
H	1.704096444810	5.049647009242	-3.743848038310
H	3.222893310979	5.815943078181	-4.306390420541
C	-3.971867415096	-1.276340291913	-6.044536474110
H	-4.568655795864	-2.162594212351	-5.728343768940
H	-4.487001530555	-0.847100894599	-6.936740816250
H	-2.983847319114	-1.649885806097	-6.392787493225
C	-11.167943228704	-3.701999285119	-5.076862930017
H	-10.923808677348	-4.675131126348	-4.585975059541
H	-12.244968992887	-3.501643352417	-4.874676864909
H	-11.065300438737	-3.864526778848	-6.173080071640
C	11.336917120841	-1.296721451443	-4.986125213671
H	11.956628537573	-2.113303529423	-5.430561445220
H	10.740025158452	-0.864500944267	-5.820041727280
H	12.044630405244	-0.506073539755	-4.646546629769
C	4.201610551015	-4.621272962795	-4.258875179653
H	4.796664242572	-4.038910207355	-4.997304135123
H	4.762079253316	-5.567751357427	-4.068537073521
H	3.248057194330	-4.908246716665	-4.755988481706
C	-1.498024504325	-3.355344294839	5.334886365277
H	-1.550697523145	-4.237570784588	4.651573954151
H	-0.433500787818	-3.029264588081	5.360581925447
H	-1.749425862176	-3.729023638140	6.352745296273

C	-8.102097525830	0.668328895911	7.574156647609
H	-7.238407384544	0.059814750515	7.925489652428
H	-7.715484636889	1.700484686624	7.395470424240
H	-8.821256618867	0.739517799825	8.420966949415

BA

C	6.953705908784	4.137828299301	-0.248894028497
C	7.066014114067	4.968372185013	-1.373725436848
C	8.460868092047	5.055187229387	-1.690899378979
C	9.172386767857	4.292503798549	-0.765514027387
N	8.244781320485	3.740945467671	0.117974497680
H	8.908161527370	5.635514490574	-2.512815701438
C	10.651470706076	4.070910228682	-0.645666789008
H	11.070539301462	4.440839257412	0.321395049465
H	10.946858413492	2.998105148847	-0.737028942293
H	11.173170409456	4.621527832287	-1.456615482046
C	8.584450105299	2.786572700817	1.169935631362
H	9.589089495723	3.055668612327	1.570229171787
H	7.878171955879	2.917829575263	2.020338778844
C	8.594062211846	1.313530094188	0.711649959705
H	7.556458926577	1.000751312578	0.443753695293
H	9.185235874377	1.238733369711	-0.230525540970
C	9.169451342920	0.369718001653	1.781497449534
H	10.230368032949	0.658503345892	1.986379700846
H	8.626153830911	0.526420794703	2.745024284503
C	9.117623295257	-1.128270603337	1.421112123033
H	9.578457412271	-1.708644915649	2.256296671851
H	8.052000814485	-1.464723530852	1.376404990243
C	9.818214001506	-1.498129485239	0.099678629742
H	9.233077894100	-1.119883264598	-0.771820491007
H	10.809715990664	-0.986612152928	0.039468077347
C	10.017183818385	-3.020814719363	-0.051125737535
H	9.064486190479	-3.545585391318	0.183244064593
H	10.758431360209	-3.395767356384	0.689879278647
C	5.703147998837	3.711550370076	0.471688116092
H	5.664694078491	2.598248551929	0.539718288653
H	4.845400550653	3.999350578308	-0.176104337975
C	5.481573495877	4.326611528066	1.890075572892
H	6.077903220975	3.745888268833	2.639944853761
H	5.928953230752	5.345401230219	1.896522597029
C	0.612459875877	3.325856658386	3.198762084409
H	0.724701526897	2.242588964125	2.976640176126
H	0.322376900742	3.372620177758	4.276776048354
C	-0.574853252198	3.882911197903	2.382050810225
H	-0.727976643995	4.970911218528	2.582793050332
H	-1.526352363120	3.404290942238	2.724612504309
C	-0.483898976374	3.692657440859	0.868299325015
C	-1.642572562480	4.234506986154	0.022374231738
H	-1.438380008255	3.928631330854	-1.026612815313
H	-1.615397172014	5.351058707854	0.045577899265
C	-3.038426846408	3.730553957019	0.474460904594
H	-2.941791659396	2.683805692664	0.850969856876
H	-3.379352408338	4.324140306225	1.359650764098
O	0.457654772829	3.109761879974	0.338910576337

C	-7.299974177064	2.548291012756	-2.035594883005
H	-7.389959088904	2.424867119278	-3.139238584501
H	-7.346958071513	1.515280064143	-1.623568150075
C	-8.540536208362	3.368095873904	-1.552669950590
H	-8.387542461416	4.422937058261	-1.875468938627
H	-8.547002263629	3.407572029508	-0.438478599312
C	-9.872519858884	2.924233609692	-2.099327843819
C	-10.453763650154	3.321790885927	-3.305165187441
N	-10.774515430679	2.059062972246	-1.470156975848
C	-11.741107203559	2.712537774129	-3.416137945569
H	-9.996002802125	4.016335988572	-4.026943370194
C	-11.923899911062	1.931710715131	-2.262571884793
C	-13.147527749087	1.143084578205	-1.865280881842
H	-14.024052381520	1.834511034255	-1.806540445369
H	-13.039093188702	0.722575713774	-0.841905802280
C	-13.563917853434	-0.013287967633	-2.832181773048
H	-14.479172259761	-0.496214267872	-2.421379242019
H	-13.818248711668	0.413261352665	-3.828010084342
C	-12.492296412595	-1.100865340968	-2.976185492170
C	-11.529651472669	-0.961341257420	-4.145031346814
H	-10.772505345505	-1.772214256912	-4.136167728890
H	-12.086820755592	-0.980081243848	-5.110511119395
H	-11.029662464128	0.035414649321	-4.095779979813
O	-12.409731729096	-2.013781302686	-2.159109421739
N	10.445186691578	-3.435318607496	-1.387098652206
C	11.758232144799	-3.371556425448	-1.864293751175
C	9.597413347533	-3.860796441260	-2.411822152756
C	11.745370268835	-3.762585999069	-3.212806342622
C	10.388838630707	-4.071290263131	-3.543504889127
H	10.019064859567	-4.421714232555	-4.520005241734
C	12.952361993993	-2.988715320196	-1.024440130782
H	13.550989599810	-2.224643564443	-1.575598380096
H	12.642478602079	-2.499210633180	-0.075203316695
C	13.893390090643	-4.167852885528	-0.686363259836
H	14.180771889204	-4.726977892887	-1.606840259737
H	14.860440021540	-3.779662133552	-0.272978347447
C	13.365767169823	-5.148107931701	0.367579847406
C	13.977258201928	-6.543998975070	0.344503215411
H	13.717825744003	-7.099526016215	1.269086750011
H	13.572457297430	-7.098746286768	-0.534831227892
H	15.082422329273	-6.507268165912	0.212798230758
O	12.519165227845	-4.831216268504	1.195045501722
C	8.106628875225	-4.016946240579	-2.276465521016
H	7.799421602031	-4.055641612844	-1.207246237528
H	7.807374122505	-5.004566906938	-2.697665645177
C	7.264647927401	-2.913839362368	-2.996975754402
H	7.623851980460	-1.910081380837	-2.678039512868
H	7.489258850499	-2.960718120595	-4.088156419707
C	5.784391675130	-3.075910828066	-2.783639685095
C	4.934062133986	-4.023450919080	-3.373437609809
N	5.053686195892	-2.402896196069	-1.793563560848
C	3.666036469354	-3.911707515105	-2.720664850890
C	3.752834878958	-2.910800886917	-1.751878216247
H	2.769788778335	-4.516528934615	-2.931478382810
C	5.524272157053	-1.256374053971	-1.013178852724
H	5.203957949275	-1.390724707609	0.046669590261

H	6.635474793254	-1.282283778950	-0.992842050308
C	2.675286650569	-2.406358854317	-0.826193123310
H	2.228711247174	-3.264629343819	-0.272327484542
H	3.092110466531	-1.748721191314	-0.031306604816
C	1.543877524028	-1.651339134036	-1.555641155868
H	1.957772206962	-0.905489059696	-2.278040599756
H	0.957955652925	-2.349411246110	-2.202983644414
C	0.559004879990	-0.926519246859	-0.639267599038
C	-0.444024509010	-0.013683506003	-1.343533691179
C	-1.618975491169	0.515510321156	-0.491203740153
H	-1.960696025571	1.455881566655	-0.979852536385
H	-1.212880377827	0.813500212681	0.499724125620
C	-2.830964012551	-0.367825731810	-0.352085973261
C	-3.869196437367	-0.535052507310	-1.283278283023
N	-3.144507269517	-1.128105475512	0.781290632064
C	-4.826669554695	-1.411286995877	-0.683364955094
C	-4.372793057115	-1.760166389777	0.594076455370
H	-5.763916333634	-1.756178665487	-1.148080107715
C	-2.373004775113	-1.132433155141	2.025840246404
H	-1.297041353352	-1.017603440348	1.769669355673
H	-2.467224224708	-2.136803775166	2.498410784863
H	0.167223168506	0.860271505364	-1.685091829592
H	-0.803010504082	-0.501990223782	-2.279666786337
O	0.622453141354	-1.030069751204	0.583894051436
C	-5.061347048666	-2.625388980678	1.618291501009
H	-4.473687210504	-2.690934678680	2.560550218189
H	-6.021590794633	-2.143344765073	1.920724060104
C	-5.378581378454	-4.073399073142	1.120227254358
H	-4.435626444095	-4.668439877259	1.137337780650
H	-5.663064852581	-4.024462329266	0.045254891739
C	-6.439728317066	-4.779880934004	1.917410753513
C	-6.332627988302	-5.430063567485	3.154419912009
N	-7.788232855756	-4.811626791753	1.534542310724
C	-7.651629662083	-5.858524677299	3.517401751768
C	-8.534614765051	-5.468107895587	2.510635505489
H	-7.933460615515	-6.412142038806	4.426606234367
C	-10.013179284354	-5.694836344289	2.393307587961
H	-10.289028262854	-6.338026136478	1.522790669938
H	-10.382042017746	-6.209439518345	3.305591289258
H	-10.594995890735	-4.747766225045	2.291539059048
C	-8.337534144532	-4.233786589820	0.309968676533
H	-7.603599342981	-4.386259454829	-0.515236719006
H	-9.240219401723	-4.816414371954	0.018073783030
C	-8.688152607886	-2.735141977116	0.415455503254
H	-9.509506106799	-2.605739756276	1.159492564780
H	-7.809987601753	-2.190686697305	0.834671353230
C	-9.068383862796	-2.113161708614	-0.940257447671
H	-10.016611421800	-2.562277896316	-1.322023486807
H	-8.281525427052	-2.380639448973	-1.687878627845
C	-9.206959169313	-0.577441055989	-0.918962816325
H	-9.322460393176	-0.213118609879	-1.966574243749
H	-8.250751828373	-0.131921306516	-0.548423921980
C	-10.380598572954	-0.052044758204	-0.076297064247
H	-11.311548733832	-0.581480059030	-0.380872211270
H	-10.229288686934	-0.308060513771	0.999625705768
C	-10.595805188435	1.476994475975	-0.134051338863

H	-11.475699291262	1.747616078274	0.497004582197
H	-9.725301858580	1.989686436174	0.330430878381
C	-2.808654212318	-0.040330041714	3.022734206267
H	-2.727688248006	0.956783838708	2.523520689002
H	-3.891063901891	-0.170166904087	3.265977366958
C	-1.978960640248	-0.034730986884	4.317759383825
H	-2.066697494680	-1.030702159962	4.816307022615
H	-0.897584698378	0.074718995231	4.061921423825
C	-2.393214805960	1.070688144777	5.300131458960
H	-3.459326471665	0.967142024513	5.607379035487
H	-1.775939410826	1.052344750119	6.225601753565
H	-2.279529865470	2.082715368820	4.846394336147
C	5.026771368883	0.116921384730	-1.523859332352
H	3.912883031835	0.110015770828	-1.523228378232
H	5.315567219413	0.884772919055	-0.765673014655
C	5.538523219599	0.551978841204	-2.913684529010
H	4.865765026489	1.354642144152	-3.297562789014
H	5.422703952541	-0.298154256909	-3.628550545401
C	6.984556632378	1.075349500159	-2.929195005702
H	7.307775451002	1.336543362166	-3.961144815819
H	7.091843402450	1.992941138684	-2.306368211711
H	7.713683943571	0.324499282405	-2.546181600474
C	4.062743891686	4.499529644779	2.377730021003
C	1.916830108872	4.059396814414	3.013324638486
C	2.199982033702	5.381228971728	3.389091750629
H	4.094911821154	6.589216459925	3.120190249677
C	3.541337370499	5.647751010335	2.977657682975
N	3.059619122449	3.526168591496	2.402413435226
C	3.182106254083	2.166595990250	1.861763293629
H	3.594298373280	2.232828574765	0.832334887330
H	2.159766165497	1.760120313923	1.719802949621
C	4.045501660623	1.210970093584	2.713691041639
H	4.255287016620	0.304781430084	2.092900007321
H	5.038473926811	1.682587620907	2.900823162072
C	3.417016536225	0.775749808944	4.054322545117
H	4.217055646175	0.353519478258	4.707074169801
H	3.044664657965	1.680711826503	4.592830543029
C	2.290374516240	-0.260765993290	3.908004290266
H	1.829490059902	-0.505196297142	4.891155072096
H	1.473927008094	0.087488031404	3.235741454180
H	2.668046969594	-1.212422112917	3.469058402214
C	-4.066670335695	3.755616233184	-0.625885956078
C	-5.974903431630	3.196693058345	-1.746145606248
C	-5.264507178424	4.101030359917	-2.550688987081
H	-3.281243326534	5.125988070435	-2.194926892002
C	-4.068733244017	4.443289423064	-1.841655052177
N	-5.237516007663	2.997636287122	-0.573169325389
C	-5.609609464797	2.119336016654	0.535553350508
H	-6.279398678931	1.328790196464	0.135806435628
H	-4.701600911018	1.570435424448	0.877278177695
C	-6.265120600935	2.851961972184	1.726395483792
H	-5.612216435301	3.703086924696	2.031092499017
H	-7.223886028436	3.316939537978	1.391136288435
C	-6.518167257236	1.950844797228	2.952289362267
H	-5.544374836508	1.530483963472	3.302753455272
H	-6.882512823479	2.590869361051	3.789979829509

C	-7.514783258435	0.803387652159	2.721739962716
H	-7.162284255370	0.088414797455	1.944619879743
H	-7.679910809659	0.215443069714	3.651687941274
H	-8.508151114305	1.188204040905	2.393822697462
C	5.936807579953	5.625027879427	-2.127037125293
H	5.427320710648	4.920807689685	-2.828247043367
H	5.151093209403	6.032715813829	-1.451712116718
H	6.310328155996	6.473363985040	-2.741455073025
C	1.270748787477	6.340492314607	4.086973179116
H	0.653390029151	6.942691472983	3.376298160612
H	0.560687673735	5.819176238363	4.769273194688
H	1.839258744714	7.070352640180	4.705473294663
C	-5.680534071519	4.624634219661	-3.900329632589
H	-6.289326877968	3.885734900692	-4.469178138051
H	-6.292954199644	5.555530663166	-3.827476884727
H	-4.794721507219	4.872414882577	-4.526466603972
C	-12.722596214723	2.907848046838	-4.542947282930
H	-12.521484611741	3.858852315244	-5.083376850891
H	-12.674774861648	2.092986857680	-5.305548331616
H	-13.776592296156	2.951785404103	-4.184168147260
C	12.933406210064	-3.857709864849	-4.134517451665
H	13.405752697258	-4.870269581306	-4.128753111492
H	12.640485988438	-3.653014657968	-5.188487500027
H	13.733146191661	-3.128763092153	-3.869885857943
C	5.299429219713	-5.007390163363	-4.453618149299
H	6.115435590969	-4.628776109773	-5.109772090518
H	5.645365732894	-5.985964155685	-4.041469891424
H	4.427128950954	-5.229011474319	-5.108557288949
C	-3.962445593925	0.106586256029	-2.643767469190
H	-4.996558228473	0.035629945774	-3.046456927647
H	-3.699669063272	1.189977167143	-2.618465543002
H	-3.290504147862	-0.373684125011	-3.394912810109
C	-5.073361102560	-5.625914799733	3.957195106568
H	-4.158564016957	-5.537935540929	3.328846928435
H	-4.972765373418	-4.879359481126	4.781787878432
H	-5.046696012641	-6.631333122287	4.435488349947

DAP

C	8.573478826910	5.135513248026	-0.083414627699
C	9.128067758132	6.281626613347	-0.671519963855
C	10.516628236763	6.299396734333	-0.319212500129
C	10.786755643002	5.183780713195	0.472182383243
N	9.593883341080	4.473043676217	0.612618771182
H	11.254394356201	7.065314005499	-0.605059610745
C	12.079602502133	4.792505106409	1.126852222193
H	11.990861746177	4.694195558970	2.235620896510
H	12.503264482667	3.832180039912	0.749337032859
H	12.837846005406	5.580296021120	0.932151967026
C	9.455458332677	3.185294569466	1.297912780396
H	10.282905592107	3.107495910731	2.039101906870
H	8.514707579908	3.178085426633	1.893558258370
C	9.457110067226	1.936310220383	0.390094802041
H	9.262250624095	1.064244836192	1.061251999165
H	8.572263484649	1.983378814404	-0.287057017006
C	10.739404716891	1.706156772251	-0.432349136692
H	10.818514688695	2.482549238949	-1.230854542006

H	11.628491698412	1.857994261591	0.225528166239
C	10.851482422950	0.297411177382	-1.052191624007
H	11.828412623213	0.209309162445	-1.585854989809
H	10.898219894970	-0.451556127626	-0.221280964017
C	9.709228295879	-0.102825751343	-2.002489617551
H	8.730620333225	0.005050552508	-1.484446672885
H	9.664470127474	0.599387917419	-2.869265630511
C	9.838736528375	-1.563298649558	-2.497300330795
H	10.239152633312	-2.193489778685	-1.671801503456
H	10.575727773128	-1.647171733003	-3.326996650869
C	7.132400850919	4.707853617646	-0.049744032512
H	7.029844517172	3.613834841255	-0.243592229489
H	6.601754667028	5.203151139956	-0.893393331106
C	6.388695799836	5.086944958211	1.274015317006
H	6.853236078479	4.536115691425	2.126860041331
H	6.587111329088	6.162801143940	1.476915842266
C	1.870922354401	2.734256026080	1.224272872368
H	2.226626789759	1.839043381397	1.779802159120
H	0.955432289532	3.075439207972	1.762921714971
C	1.462415838919	2.289457314668	-0.222665967922
H	2.338522548890	1.839294059478	-0.740207767636
H	1.140287610961	3.189089887948	-0.795213166874
C	0.336185306727	1.264061134632	-0.152772613940
C	-1.077436196929	1.837288493696	-0.037691743625
H	-1.353268941457	2.224498025661	-1.051312619863
H	-1.031568980865	2.754906645686	0.597386551589
C	-2.145362284145	0.863653138967	0.517611953805
H	-2.436530942529	0.109219480629	-0.248596731051
H	-1.675089361136	0.274423163055	1.334376705861
O	0.564399328794	0.055228443557	-0.114893669603
C	-6.450817087783	3.585294315589	0.788791977791
H	-6.440850783911	4.590402666586	1.274044662444
H	-6.398982559904	3.788010935134	-0.304667743342
C	-7.799118852861	2.892970792140	1.109652743208
H	-7.903793813170	2.824777122617	2.222252033023
H	-7.736672918347	1.835275347335	0.760687073702
C	-8.989636237315	3.585380139959	0.498258070543
C	-9.082699006635	4.844034520634	-0.099364130324
N	-10.267881530261	3.025797763366	0.485632892219
C	-10.444830173215	5.061051828058	-0.487977805654
H	-8.253779879190	5.555122294869	-0.232029018686
C	-11.163418077907	3.916741958460	-0.116883954555
C	-12.619736853226	3.607418240023	-0.336127709189
H	-13.230463171215	4.498715531406	-0.056734846669
H	-12.976096558149	2.798639356109	0.339890705134
C	-12.969585233588	3.214847845931	-1.789124719631
H	-12.848116992615	4.078946026823	-2.484888015710
H	-12.244561658775	2.452629679388	-2.172922561804
C	-14.365463623084	2.601967115795	-1.960805684385
C	-14.896562307143	2.535397377545	-3.390648900431
H	-15.798704436595	1.891452345898	-3.439380094934
H	-15.168958897256	3.561796848244	-3.732019853679
H	-14.120909963079	2.164311132666	-4.098406601189
O	-15.009523963598	2.177586639826	-1.010195062877
N	8.576727525696	-2.168469441913	-2.931088004776
C	7.967523041868	-1.973258500646	-4.172850214555

C	7.752706636155	-2.962145875765	-2.128806981963
C	6.738490238837	-2.655456957353	-4.162305746841
C	6.618322893965	-3.276430530096	-2.879194102256
H	5.771352946110	-3.880398701520	-2.519216552331
C	8.589284896387	-1.196307399256	-5.307201869179
H	7.841139766549	-0.466108675106	-5.698194379778
H	9.448314501562	-0.584874669536	-4.953867759647
C	9.056516045789	-2.071403233958	-6.492721273586
H	8.235671124990	-2.737174956027	-6.847120813183
H	9.300858637546	-1.423658968399	-7.374250328421
C	10.317567672019	-2.904790305855	-6.237133486371
C	10.507383317244	-4.119027018513	-7.139233239271
H	11.538033640843	-4.518365932286	-7.044018074607
H	9.783887602030	-4.911159952314	-6.832864704559
H	10.282366368112	-3.880631855808	-8.203483931948
O	11.138141884429	-2.610306518969	-5.376171152921
C	8.065082915416	-3.317349466567	-0.702815386759
H	9.138940580163	-3.606623804496	-0.594344717017
H	7.483384324976	-4.232364691642	-0.448483519048
C	7.749169825089	-2.211927815853	0.363381237052
H	8.272555065541	-2.492145619316	1.305832862635
H	8.222701260594	-1.255074466572	0.051825251148
C	6.288569300705	-1.952333916812	0.609730770901
C	5.410617441566	-1.106734772834	-0.089415703733
N	5.529232969364	-2.665200194035	1.552486871280
C	4.101571029066	-1.321142441294	0.451974170492
C	4.190305530715	-2.278217345008	1.466378018675
H	3.173637973781	-0.833676192040	0.112861906102
C	6.060730878566	-3.549603686427	2.591306970575
H	5.343411832932	-4.388500949635	2.730797146336
H	6.996077796995	-4.020472391710	2.215296705774
C	3.100353180408	-2.826756220008	2.352754268712
H	3.399344116317	-3.800528750279	2.803763764872
H	2.917529646011	-2.160781692298	3.232501461478
C	1.776502135660	-3.027963030077	1.598360503103
H	1.412392415683	-2.063607498676	1.167713732938
H	1.929486251318	-3.678815646135	0.703157220220
C	0.648210923105	-3.622043163643	2.444323408851
C	-0.659332436366	-3.918493720641	1.701181361001
C	-1.453273779374	-2.618371443756	1.353104284137
H	-0.775268903862	-1.906835086763	0.831324733400
H	-1.739009738474	-2.115599830916	2.305925940102
C	-2.637509370510	-2.851760729820	0.458013225101
C	-2.645666472203	-2.952168607752	-0.940762974856
N	-3.919409284858	-3.174733490468	0.919438979689
C	-3.969846285736	-3.350239463615	-1.319926377566
C	-4.742849975295	-3.481092577847	-0.162680386480
H	-4.315064487871	-3.539778431740	-2.347165259366
C	-4.343673165862	-3.067610321055	2.311522631853
H	-3.580561999326	-3.556376965555	2.960857313932
H	-5.276935017572	-3.661373845874	2.443181433010
H	-0.435795909434	-4.456073066362	0.750493492460
H	-1.274477668195	-4.590976113344	2.339643782738
O	0.770184370025	-3.829263666976	3.647216821186
C	-6.183432809516	-3.892463648132	-0.014268583152
H	-6.283793578784	-4.646518354309	0.803786711828

H	-6.809957576492	-3.022817004471	0.300842850248
C	-6.774262789885	-4.507167615626	-1.310326166168
H	-6.067505694518	-5.291639221960	-1.672534611264
H	-6.784452728569	-3.726318392413	-2.104402540666
C	-8.135766645001	-5.127029736874	-1.138404479385
C	-8.464857491093	-6.334332610494	-0.504314046732
N	-9.324682937826	-4.553252871969	-1.601598272936
C	-9.888481384260	-6.473647248118	-0.582307488782
C	-10.402851787091	-5.367227979860	-1.257845408424
H	-10.486297262221	-7.312199102164	-0.192297250054
C	-11.824038103548	-5.041759297836	-1.612838026082
H	-11.998901772730	-4.985446201771	-2.714697604151
H	-12.492443221481	-5.836933230517	-1.220150580966
H	-12.176360666980	-4.074947073601	-1.179248586442
C	-9.444987483283	-3.265623325091	-2.279129952403
H	-8.620045723168	-3.163103873869	-3.022764126270
H	-10.386452030327	-3.275625555755	-2.873513455502
C	-9.440692345041	-2.063095037213	-1.318259893868
H	-10.200316391769	-2.247077604988	-0.521693978782
H	-8.449754483804	-2.009199660146	-0.808983624853
C	-9.697664952270	-0.718470860618	-2.017495291348
H	-10.693153369131	-0.731709326195	-2.528469211037
H	-8.939733130376	-0.585231406665	-2.826031622560
C	-9.611305442608	0.486209473368	-1.060518181922
H	-9.548208768058	1.430609239963	-1.650582661538
H	-8.644194545384	0.407835400144	-0.508773577437
C	-10.777841934714	0.596638578165	-0.062137680735
H	-11.732387913382	0.751831900794	-0.619341238048
H	-10.913379497159	-0.364312043293	0.489049087284
C	-10.607021911428	1.700555835178	1.006339211371
H	-11.537867965261	1.778121903936	1.614517426192
H	-9.804970112878	1.406030527755	1.717970418303
C	-4.572365943066	-1.620251374647	2.828156027482
H	-3.646341626950	-1.037725416127	2.611803245664
C	-4.778814593317	-1.633157158167	4.357872969308
H	-5.688362407471	-2.215296219645	4.639633135095
H	-3.912750101682	-2.083170331996	4.895625924853
C	6.297624041090	-2.852291461734	3.952868776438
H	5.433869540796	-2.179866996432	4.159711364806
H	6.288280359499	-3.629359261884	4.756774025209
C	7.600793033061	-2.025810417000	4.069199544925
H	7.734898716821	-1.451281805432	3.119979734988
C	8.840051704377	-2.927481584334	4.259253349352
H	8.774973650061	-3.497359545792	5.215796192808
H	8.954840597195	-3.670767030073	3.436155057109
C	4.895282482326	4.902150209401	1.260434560057
C	2.872054625235	3.851908481361	1.263454688703
C	2.625502797408	5.230853495710	1.172463327021
H	4.084243219106	6.963397108834	1.119337429092
C	3.900942925798	5.878889604108	1.172350067097
N	4.257431661157	3.662618907156	1.309180427272
C	4.948700884493	2.386933291303	1.475839376497
H	5.872598302141	2.398961999681	0.858405683778
H	4.317906698175	1.570689596425	1.056323335246
C	5.365946061141	2.019815064554	2.925996806486
H	5.831849098715	2.931893887083	3.377528604179

C	4.167872662388	1.620082502285	3.813565844656
H	4.512673734635	1.372529217524	4.842958176510
H	3.417165246210	2.438713035957	3.901406602019
C	-3.340018980833	1.557873651741	1.112688477317
C	-5.241392372974	2.812516654818	1.244139654710
C	-4.748612106198	2.664004593959	2.550353463521
H	-2.889795403321	1.594040520042	3.287742597603
C	-3.554163904238	1.878156841635	2.456251158908
N	-4.377537020484	2.128657984857	0.376967815823
C	-4.543475119132	2.011723112954	-1.072937420039
H	-4.682478989844	3.032524417250	-1.504785528960
H	-3.589601213808	1.624263809760	-1.495075886056
C	-5.714899644703	1.087267590868	-1.547861831216
H	-6.661657749860	1.565236640048	-1.195379347394
C	-5.753495236816	1.089067772425	-3.086587143236
H	-4.826988192512	0.633237567212	-3.510058622001
H	-6.616142389987	0.488579204490	-3.447880268599
H	3.658770471424	0.715336929272	3.404967895449
H	-5.843804254874	2.117188519885	-3.502776413133
H	-4.909678700107	-0.596347223218	4.740765443778
H	9.776837193736	-2.325325381105	4.283364694271
N	6.415252862223	0.991577268199	2.824139603347
H	6.599831367424	0.576209333476	3.757465378993
H	6.067065682624	0.206657681300	2.243782493113
N	7.432446621451	-1.009276695111	5.129355199369
H	7.200483883928	-1.466521285695	6.027525230031
H	8.327995014603	-0.520406719982	5.291319408300
N	-5.649142762789	-0.942749395142	2.086753517567
H	-6.571624247204	-1.306363271543	2.379954553477
H	-5.639555145661	0.063916822967	2.326937726416
N	-5.709144812668	-0.282880193630	-1.052617663802
H	-4.922267516964	-0.833463724475	-1.434756030034
H	-5.641969027523	-0.362657071880	-0.018737258979
C	8.408053602823	7.326510036680	-1.482799324151
H	7.454962453230	6.945996630686	-1.913122314833
H	8.150786766995	8.229666327526	-0.878846914454
H	9.034078596394	7.680596420665	-2.333091919439
C	1.279760631295	5.898332023599	1.064463500067
H	0.956749852434	6.043769387805	0.005012638761
H	0.475647867020	5.313127149711	1.566970145835
H	1.291555815204	6.907214978902	1.533846936193
C	-5.347456583421	3.236598297458	3.809061600353
H	-5.952041723246	4.149636623485	3.609095446892
H	-6.020694751805	2.514920254514	4.331921957427
H	-4.556577845342	3.518842510189	4.539622933923
C	-10.995159940087	6.282326649323	-1.176773832554
H	-10.874637461369	6.238810442544	-2.286378466705
H	-12.081247142322	6.423316306658	-0.975736192662
H	-10.476822849466	7.207288038806	-0.837710862968
C	5.740758275857	-2.733778139065	-5.288421647100
H	5.767130338470	-1.831094788317	-5.940560940268
H	5.906964253433	-3.616749053581	-5.952617572552
H	4.702679906625	-2.824191148209	-4.897627184257
C	5.752680886484	-0.151634234107	-1.204337647504
H	6.583708107884	0.536907663185	-0.924025024036
H	6.057523011751	-0.675370384219	-2.138734652960

H	4.876800538465	0.486436203406	-1.455018611183
C	-1.471961454789	-2.752227383712	-1.864514212214
H	-0.775282658929	-1.962744742736	-1.502615842597
H	-0.867198089858	-3.682950867297	-1.991278221759
H	-1.810478318580	-2.456236825757	-2.882639945272
C	-7.504946191962	-7.299377979180	0.141527489616
H	-6.484916833179	-7.238697049153	-0.300904969245
H	-7.390259118718	-7.121199313188	1.238353220082
H	-7.850899464973	-8.351287411738	0.026771428153

HAMC

C	-6.905641908434	6.083582168529	0.450906815881
C	-8.034246624710	6.909531851564	0.340461401908
C	-8.913674066565	6.558402293156	1.414853217721
C	-8.323773544819	5.534970001475	2.155381518831
N	-7.098691526007	5.241072298173	1.556997717030
H	-9.887099893966	7.021043837589	1.640580026729
C	-8.841133560260	4.847916282559	3.386181721834
H	-9.229901613301	3.819496850484	3.191341540495
H	-8.071276530059	4.757050048310	4.187948028681
H	-9.683709960830	5.435976409017	3.807970977156
C	-6.185586766258	4.181966260538	1.991322383486
H	-5.257807272619	4.279048029531	1.388835945125
H	-5.877688276284	4.367246238654	3.049221806179
C	-6.740105737453	2.736994541762	1.873157687676
H	-5.874506396255	2.038949481057	1.986221969669
H	-7.396851355544	2.529989906778	2.748206248418
C	-7.506929611291	2.425946470830	0.575095583955
H	-8.347058299400	3.153445970242	0.466843606993
H	-6.852983740489	2.587072489417	-0.312634888891
C	-8.071256199064	0.994268857351	0.489104522397
H	-8.616522549346	0.901177440425	-0.480650950130
H	-7.227826937850	0.264914928736	0.423439529812
C	-9.003941572062	0.575767655752	1.641412982201
H	-8.416701626388	0.352078053424	2.562851306900
H	-9.680224652855	1.423578429267	1.909748516539
C	-9.856501130931	-0.660679882533	1.278293753393
H	-9.216170288735	-1.424478843278	0.782785752569
H	-10.640011330709	-0.388150781573	0.535075601176
C	-5.646606816566	6.112775979782	-0.373619234269
H	-4.751256309725	6.192227951394	0.291511160670
H	-5.648342228179	7.061323152902	-0.955492829233
C	-5.456157641630	4.937876349213	-1.382688215563
H	-5.407886775745	3.969803097927	-0.835855657608
H	-6.372459065374	4.863251693577	-2.008262982754
C	-0.639245329875	4.818081945838	-3.053891213196
H	-0.383395413035	3.891800893323	-2.493083554980
H	-0.330846761639	4.608281497255	-4.101846543231
C	0.272694468207	5.974973276483	-2.545366205664
H	0.055699361745	6.245945454360	-1.487180142828
H	0.083932033509	6.888989980202	-3.157340282578
C	1.739246611432	5.578524274616	-2.720013002318
C	2.642572668302	5.633547934392	-1.499717590579
H	2.176217490652	4.963849347848	-0.733285069067
H	2.560195668824	6.654327778947	-1.050095922997

C	4.119185078083	5.265076640168	-1.759668269860
H	4.170498996904	4.239208622160	-2.188911540893
H	4.508704190121	5.931943323107	-2.561376878565
O	2.134674032025	5.184268684670	-3.821229735030
C	6.543270884004	4.079103539669	2.567989013010
H	6.666335816800	4.760467356951	3.441945874664
H	5.776946914759	3.332869719963	2.881550264210
C	7.890114480146	3.352991392365	2.318164703408
H	8.635665016705	4.119207827576	1.988089318509
H	7.769502773165	2.674039869052	1.439139601828
C	8.409681835249	2.595788217391	3.512337083333
C	7.929504318953	2.549884387360	4.822456523999
N	9.583689274613	1.840954166042	3.473450402102
C	8.827773538822	1.753778174992	5.605490726972
H	7.027981854833	3.059299359616	5.194162572459
C	9.848494322355	1.323781930606	4.747278684527
C	11.033669281324	0.450241773449	5.060425225082
H	11.512581525060	0.809943210521	6.002385896708
H	11.827292553588	0.544104431940	4.286631517532
C	10.695138353810	-1.048726147770	5.231670351251
H	10.136547146707	-1.235976655148	6.179137382390
H	9.989685611019	-1.383511242070	4.429157697230
C	11.905786121623	-1.987106444532	5.141889317893
C	11.706337051078	-3.393091849604	5.700982045338
H	12.550008948947	-4.051049217045	5.407519219244
H	11.655350395635	-3.345932804736	6.814198681919
H	10.740812403489	-3.832027260717	5.360756903115
O	12.961761141606	-1.636953637245	4.630443692567
N	-10.496078235685	-1.300668016853	2.428429015679
C	-11.585283889792	-0.783450455539	3.134902950038
C	-10.025012404064	-2.446145079213	3.070929490109
C	-11.807225694587	-1.608728831989	4.249199246324
C	-10.825974253769	-2.648120567447	4.197525494549
H	-10.718545521815	-3.483122177555	4.907547751667
C	-12.389987615551	0.416602836211	2.698284170034
H	-12.515661814243	1.105502500059	3.567621070861
H	-11.854835095926	1.004785832012	1.921495805404
C	-13.799227513010	0.069180822396	2.166211013337
H	-14.359156309501	-0.562889012992	2.894003471035
H	-14.412107454642	1.002501428720	2.066189657842
C	-13.831977671098	-0.590626402649	0.782747483294
C	-15.086646251936	-1.393034456482	0.456715664125
H	-15.127055245158	-1.630431238748	-0.626232493045
H	-15.062950660004	-2.347322064077	1.034297014242
H	-16.011456726569	-0.857452413388	0.769733823637
O	-12.918958207981	-0.470270471664	-0.025154563334
C	-8.882637381744	-3.293539753936	2.579503083990
H	-8.971174103711	-3.484826599562	1.481767288285
H	-8.991245885406	-4.295248444895	3.052535390165
C	-7.439799506200	-2.781126407295	2.897433025739
H	-7.253215007676	-1.809180457579	2.381833173945
H	-7.394820395764	-2.546137633701	3.985312699513
C	-6.363271256356	-3.783738590844	2.582227802354
C	-5.837396229439	-4.786827495131	3.410995000849
N	-5.795417020157	-3.939257484410	1.313184020122
C	-4.924375564159	-5.549757196792	2.614450314153

C	-4.905251901759	-5.013356272146	1.325449362872
H	-4.330624160942	-6.413268859257	2.953898752452
C	-6.048325142929	-3.081199903923	0.157966907911
H	-6.086822667428	-3.726549785554	-0.748073731009
H	-7.069753103826	-2.649307942541	0.254526785556
C	-4.049362075661	-5.407869866461	0.148878091005
H	-4.152301150876	-6.504427578317	-0.031260011132
H	-4.390692815985	-4.921547144140	-0.791291337920
C	-2.552633768566	-5.076451833114	0.350313595048
H	-2.429903492300	-4.015751652893	0.682602819377
H	-2.120056310225	-5.683706316455	1.178536256434
C	-1.686978614885	-5.192795585097	-0.906292053985
C	-0.227192627953	-5.572376497707	-0.648063756368
C	0.679442593159	-5.590950964523	-1.891550600622
H	0.416717661813	-4.701768858881	-2.508896485100
H	0.399339848355	-6.464298665434	-2.532566574078
C	2.158096837724	-5.602340990983	-1.574016271684
C	2.858292574279	-6.209483976636	-0.517634912367
N	3.103794972605	-4.958746509214	-2.388343865761
C	4.245799062685	-5.872819211835	-0.689473295406
C	4.380079233497	-5.103438387954	-1.852710958092
H	5.072678338403	-6.228724807103	-0.056484181932
C	2.803349246960	-4.199924642345	-3.606591526703
H	1.892947719124	-4.633729925354	-4.076771079549
H	3.629964681136	-4.369229662853	-4.334251430265
H	0.156135562038	-4.860371085440	0.123138569770
H	-0.241045799319	-6.559904117855	-0.130339283467
O	-2.119205468739	-4.958194107895	-2.030666391118
C	5.619366249931	-4.495788524419	-2.457457692957
H	5.559771863144	-4.531485666065	-3.570162780677
H	5.695228568634	-3.411743104337	-2.194448693283
C	6.922902034728	-5.202655777469	-2.004522858008
H	6.809911798440	-6.296958145568	-2.201292890949
H	7.006783329370	-5.105949875537	-0.899674497726
C	8.173805969033	-4.703940597469	-2.680567665438
C	8.545264304985	-4.865098030432	-4.024279545412
N	9.217074781847	-4.030537824661	-2.029323536983
C	9.837311591475	-4.269260468740	-4.176734969356
C	10.238985458746	-3.764968122110	-2.940115963074
H	10.432409898242	-4.226149603354	-5.102254652658
C	11.527150097712	-3.094497394606	-2.562909820229
H	12.064197460913	-3.619141125507	-1.736414789548
H	12.208780632357	-3.087473766081	-3.439495502378
H	11.399655401971	-2.033569639700	-2.239865798949
C	9.267341949655	-3.665353754722	-0.615022911310
H	8.539621468228	-4.301029693428	-0.064061214514
H	10.271372885253	-3.939485015912	-0.211429315970
C	8.977073421728	-2.181505801312	-0.308380842034
H	9.782917776556	-1.547564811690	-0.748197032453
H	8.027937021802	-1.882605775685	-0.810997207384
C	8.850014647833	-1.928132467637	1.206117009543
H	9.666719680525	-2.468281686129	1.749108473963
H	7.895025830485	-2.386790734890	1.549138882553
C	8.882241604519	-0.441187030071	1.621367008435
H	8.381762021579	-0.330334149592	2.612030075691
H	8.265546361986	0.158628720845	0.907663527590

C	10.302191165836	0.146962769621	1.721212263559
H	10.925402511681	-0.522220211061	2.359677158330
H	10.802509716853	0.141969822352	0.723182832117
C	10.377980024412	1.592913628019	2.267211239996
H	11.443788340570	1.855543629542	2.466462564184
H	10.030864976873	2.308017497140	1.490967139127
C	2.617631893138	-2.693088111918	-3.356249700528
H	1.781911290352	-2.545303351617	-2.634707128476
H	3.528139596149	-2.308882500015	-2.840818298445
C	2.352117257231	-1.882610811624	-4.636678724907
H	3.240513548111	-1.942984874920	-5.312766605598
H	1.510049407543	-2.348934664060	-5.203064695828
C	1.999686631579	-0.402225977307	-4.380271908233
H	1.797022026238	0.100372351097	-5.354500369911
H	1.051136979616	-0.343484393732	-3.795684297910
C	-5.020385042888	-1.946487973841	-0.034810481868
H	-5.072103721359	-1.265583823419	0.848255028676
H	-3.988982087755	-2.371351730776	-0.023194947555
C	-5.237010118946	-1.139120388409	-1.330725203669
H	-4.591067002024	-0.231578508305	-1.292960296392
H	-6.278961420436	-0.740408085234	-1.363096226277
C	-4.954882748086	-1.925072845847	-2.625544090186
H	-3.900111777358	-2.297925060626	-2.615772933054
H	-5.581692614697	-2.848133317911	-2.643343055888
C	-4.281547572140	5.066395599905	-2.313191966183
C	-2.119409077082	5.063855344236	-3.052828577715
C	-2.926393103422	5.446366851975	-4.134611687339
H	-5.181042896155	5.692748514573	-4.245557221005
C	-4.281868911976	5.434795195068	-3.664269788515
N	-2.953660634984	4.849733258989	-1.945833359121
C	-2.519987367858	4.442112384307	-0.604036995270
H	-3.102854064290	5.036405899266	0.134300603957
H	-1.461872639988	4.761754350933	-0.473737388608
C	-2.647269088094	2.943048013779	-0.252385466465
H	-2.494130097895	2.867824884304	0.852280270325
H	-3.691390590974	2.595574258095	-0.436159261856
C	-1.644515909592	2.016623879497	-0.958710979816
H	-1.898542111861	1.935687229402	-2.042780849990
H	-0.628486070478	2.478854598684	-0.919742042784
C	-1.545521565593	0.617539268270	-0.326325665325
H	-1.287318060335	0.726846004547	0.754471361054
H	-2.531450042672	0.098069180607	-0.346585096449
C	5.028129089423	5.404196618930	-0.569072551584
C	6.074676893919	4.894863898587	1.392885806495
C	6.497325752852	6.177310325454	1.013183006275
H	5.939724504354	7.414361373058	-0.801572902338
C	5.835739437027	6.486240465184	-0.217778719769
N	5.172812881670	4.434866474730	0.423978193275
C	4.487801379932	3.142213090199	0.451806238769
H	4.252893117638	2.901504893703	1.513479067661
H	3.503810718814	3.244387025130	-0.058586733315
C	5.260973380743	1.971791857608	-0.196268873469
H	5.336968441964	2.153495957399	-1.293414215044
H	6.310083998755	1.962482671084	0.181597479888
C	4.601327771246	0.601340804430	0.065764424413
H	3.507808614532	0.670066596940	-0.152692406644

H	5.010724350071	-0.139062627283	-0.659813261426
C	4.817030326003	0.059923628668	1.495231214042
H	4.532363714015	0.830350027679	2.249594955529
H	5.901571457765	-0.136154311336	1.658093336767
C	-0.472492332443	-0.241210078028	-1.024309683788
H	-0.863758175493	-0.621426287258	-2.001107012805
H	0.420584109218	0.374142665869	-1.275746498294
C	-0.032745356483	-1.456189426127	-0.220932802484
C	4.013528392860	-1.212778413893	1.812477229462
H	4.156732800171	-1.495728831810	2.884426194833
H	2.921141309948	-1.036629888531	1.693686486541
C	4.419088725282	-2.438287691072	0.996325310526
C	-5.225326995178	-1.166947523134	-3.942015834397
H	-5.145574645445	-1.901050764819	-4.776286391422
H	-6.278820579991	-0.800257802339	-3.954585127398
C	-4.281854086797	0.008095723069	-4.262031652708
H	-3.246235373316	-0.191319745720	-3.889973717139
H	-4.155087103232	0.122033959052	-5.364501046646
C	-4.704053504992	1.384660319301	-3.744199258175
C	3.103548471120	0.379820001932	-3.625942497116
H	3.288316070101	-0.087961141655	-2.634154188569
H	4.051873620949	0.349946877735	-4.209190130435
C	2.709854852277	1.829315384708	-3.367505454603
O	1.307161898753	-1.662604314152	-0.344210480363
H	1.591101939431	-2.466091709944	0.167391238838
O	3.399298959329	-3.330712867175	0.870885946145
H	3.729785876495	-4.141987221161	0.366360226544
O	3.186756288322	2.663892175820	-4.309397488023
H	2.872431645961	3.601738468566	-4.124905549629
O	-3.842513143804	2.333629211697	-4.184063237297
H	-4.150380152275	3.234540878545	-3.875975539699
O	-0.760721722551	-2.170838764765	0.451040375627
O	5.532317607254	-2.633006522034	0.530520124100
O	2.020011632782	2.191200650798	-2.413649511982
O	-5.680752941017	1.624241362165	-3.048138382447
C	7.746105719511	-5.551857033045	-5.101624593475
H	7.120722651232	-6.381577173078	-4.699526704902
H	7.054717460927	-4.856459764939	-5.637165371828
H	8.412897386182	-5.991776868626	-5.876412278814
C	2.332527872658	-7.081180909231	0.597628364769
H	1.775191083255	-6.509406551346	1.375466759259
H	1.650325051179	-7.879741974541	0.227951127199
H	3.174243692018	-7.591220675809	1.114893637508
C	-6.194322310850	-5.043107153752	4.851738792823
H	-6.532838539595	-4.119059940535	5.372586310993
H	-7.016432022151	-5.791062582372	4.960573082750
H	-5.323380897037	-5.441122434746	5.419359746246
C	-12.885361080364	-1.445757054415	5.288922370097
H	-13.830420990908	-1.979149180082	5.022314009767
H	-12.559833570762	-1.854792651163	6.271380344994
H	-13.155831903352	-0.377407042135	5.453000813299
C	8.697596168077	1.439150372280	7.072765091190
H	8.045069026237	0.553182755058	7.264745592842
H	9.683072505311	1.221152581408	7.543281087781
H	8.249082747925	2.290763419864	7.632018856485
C	7.483869642158	7.051038876934	1.742541982325

H	7.528610134361	6.818086553101	2.830136556745
H	8.522659328213	6.941835332204	1.347904382281
H	7.218901103683	8.127908573060	1.646610845528
C	-2.473932014238	5.785830637020	-5.529469444026
H	-2.367733021070	6.886378691159	-5.680678716165
H	-1.489508257649	5.329665726691	-5.775807357043
H	-3.203818158175	5.426549247950	-6.288661219371
C	-8.293557609701	7.967486930269	-0.699494945032
H	-7.361437775822	8.305483572989	-1.204794443492
H	-8.981508601337	7.608160119720	-1.501664112556
H	-8.769393041083	8.868899558232	-0.251206112801

PAMBA

C	6.196883121766	-0.526197894792	-3.310670556269
C	7.082514178555	-1.331880713210	-4.041806592414
C	8.366214982791	-1.213610128975	-3.416604501701
C	8.248688045863	-0.347863900999	-2.329083234170
N	6.922936643276	0.078212699519	-2.277518546229
H	9.296177373323	-1.711282772109	-3.732673710791
C	9.291152534513	0.100020517859	-1.347382016895
H	9.432737679265	1.207565221240	-1.340587476256
H	9.067017457576	-0.203823603780	-0.296826947440
H	10.269607733570	-0.351339431405	-1.615303323330
C	6.345944160252	0.847961917665	-1.177293492947
H	7.097850620419	1.596504864554	-0.836301980923
H	5.488415036952	1.443300265345	-1.568547600782
C	5.881553314953	-0.020621156851	0.010574292741
H	5.195630486925	-0.814151266022	-0.368405134744
H	6.765580623730	-0.557500531612	0.428278900376
C	5.174437873872	0.794963249145	1.106190046665
H	5.842134282590	1.625772893384	1.446667293004
H	4.277955054705	1.293008808306	0.662628783027
C	4.720635169176	-0.017545422425	2.336153402999
H	4.120329997435	0.654474143084	2.994316217322
H	4.020006538465	-0.824780547080	2.011050558128
C	5.863213003772	-0.637414788533	3.165134458701
H	6.387894782340	-1.438045698697	2.593033810885
H	6.633811517109	0.141586021618	3.378570897996
C	5.343011892413	-1.221747694555	4.495939249963
H	4.541008908033	-1.964711474179	4.286789354278
H	4.860072256617	-0.411690080733	5.083596259164
C	4.746823233635	-0.247760980633	-3.587557781496
H	4.207967577064	0.068998454105	-2.665875301356
H	4.250361736396	-1.195376390270	-3.903265188579
C	4.475741425284	0.802062402967	-4.714209078841
H	4.970751414507	1.767839539915	-4.455562944837
H	4.979933885751	0.458413437597	-5.645031998133
C	-0.323414915609	2.502174980576	-4.323944322288
H	-0.034962185346	3.562976602299	-4.138898448011
H	-1.032591142948	2.546316077139	-5.179627519793
C	-1.133875441157	1.944585138046	-3.083964401639
H	-0.934058052978	0.856981139321	-2.965925410333
H	-2.221200976326	2.076335416948	-3.284053060557
C	-0.786999287921	2.671396220000	-1.795410245248

C	-1.510040699543	3.988038296240	-1.537883791905
H	-2.597586463289	3.727224814826	-1.485801400462
H	-1.447953604464	4.608808793460	-2.465099357236
C	-1.073293955215	4.788236273244	-0.279741444202
H	-0.454228417415	4.123375397190	0.366944934949
H	-0.403135913155	5.621799480531	-0.584228078749
O	0.047701313721	2.231832862079	-0.993646252544
C	-5.360880966265	4.780414997848	2.401189083112
H	-5.129132654551	3.843985902683	2.960170975976
H	-5.758252411110	5.479480144611	3.171735639597
C	-6.482707876682	4.500992489961	1.340391988481
H	-5.997360862507	4.070528797698	0.434422983273
H	-6.888501095837	5.482924859251	1.007354692639
C	-7.633465440310	3.639175759328	1.783234814368
C	-8.853782595685	4.005169101298	2.352107056913
N	-7.623953033297	2.246832758730	1.686097196168
C	-9.610692486268	2.817551370158	2.605290928059
H	-9.169694761301	5.037719623631	2.568500426038
C	-8.832985240131	1.732006826146	2.165590400576
C	-9.162734149531	0.258157150606	2.222823556496
H	-9.274287511008	-0.055561574691	3.289833409393
H	-8.327900494526	-0.353250752709	1.815884506367
C	-10.458266546114	-0.146310609129	1.482521384437
H	-10.759644881254	-1.179302491384	1.796322918212
H	-11.316176302621	0.497687081859	1.779803313376
C	-10.376171191207	-0.209789815791	-0.046617627335
C	-11.697279683769	-0.056635655247	-0.789333087855
H	-11.594910908084	-0.373324051894	-1.847890964449
H	-12.516845786066	-0.625559782392	-0.294969104863
H	-11.998470576132	1.017550626730	-0.762774596941
O	-9.320755701774	-0.399017704175	-0.641429558440
N	6.353750061006	-1.880272919224	5.327945703988
C	7.325178347383	-1.236120905504	6.105028300298
C	6.522410733542	-3.262106161199	5.431844180120
C	8.120566107183	-2.224644818466	6.707167963304
C	7.609520280923	-3.488328755055	6.278434021962
H	7.991651970419	-4.480664019899	6.564463821345
C	7.465582226123	0.256378554305	6.275203433284
H	8.555489485044	0.485392493783	6.305077307234
H	7.064649278524	0.815694458435	5.401257505013
C	6.826352649142	0.849436849695	7.560729298826
H	6.869341814781	0.130931032792	8.412949731034
H	7.412440026967	1.737965329165	7.911252543603
C	5.401452071423	1.367884913767	7.402616393744
C	4.583900287030	1.607660726782	8.655949914833
H	3.712282893124	2.260564949604	8.437355632983
H	4.210072571838	0.623334676816	9.026714896615
H	5.201686128938	2.037949007670	9.475211149151
O	4.942563335438	1.594358978438	6.280154421693
C	5.671609961478	-4.293997433133	4.742344662555
H	4.584116690737	-4.110913697362	4.928777571058
H	5.890440897837	-5.266605749868	5.236369974120
C	5.918889154220	-4.473543438244	3.207714431485
H	5.477042743438	-3.617700211277	2.644305712288
H	7.016112553099	-4.405894373370	3.032989492854
C	5.446391285831	-5.786679060101	2.647487691304

C	6.184969936922	-6.951948260633	2.394981053073
N	4.105706816478	-6.055455741475	2.341340379164
C	5.259949969443	-7.932096466092	1.907773710322
C	3.984835231425	-7.366734951712	1.873854546686
H	5.498074163879	-8.968307511365	1.620772489015
C	3.052852126435	-5.050532125783	2.324907270357
H	2.065392420402	-5.565319657332	2.361045244885
H	3.106012006405	-4.441355333518	3.257137258164
C	2.697731820695	-8.011077015199	1.427450604693
H	2.865146702579	-9.108330794098	1.486917987296
H	1.865643264803	-7.795301603974	2.139472835383
C	2.207223281590	-7.655630258871	-0.002808578422
H	3.013137650233	-7.177005462008	-0.609435990062
H	1.954109520205	-8.580346621430	-0.580416862674
C	0.961170772713	-6.772949948408	-0.081285340206
C	0.510421892437	-6.320353604101	-1.475883944022
C	0.222698219865	-4.790002341791	-1.517778494638
H	1.192654533478	-4.254233390303	-1.584183181268
H	-0.211138798570	-4.498171837579	-0.533564281423
C	-0.660154650255	-4.319717832996	-2.635917221740
C	-0.348880661140	-3.698063829287	-3.850960143317
N	-2.052944068892	-4.464299010597	-2.576355648680
C	-1.594860392466	-3.456938330749	-4.527646014735
C	-2.635606391086	-3.929353512832	-3.725272509014
H	-1.709235350391	-3.003874988880	-5.523924951335
C	-2.773612827012	-4.947275414459	-1.406872538514
H	-2.329093084762	-5.907134272089	-1.056148601124
H	-3.813271783233	-5.200239580777	-1.717778668804
H	1.243663801160	-6.610803562856	-2.261680571241
H	-0.423106197884	-6.892249101476	-1.700241565028
O	0.318466227624	-6.447089087614	0.913751104632
C	-4.126764168288	-3.922523283454	-3.935786647742
H	-4.534631078528	-4.964284488432	-3.924132628280
H	-4.633005753883	-3.408076997281	-3.082463466673
C	-4.585865524118	-3.245904230174	-5.253674833001
H	-4.214595174849	-3.848085443090	-6.115488186872
H	-4.080571314511	-2.256672620061	-5.348073962584
C	-6.080216974796	-3.100697338052	-5.357157860896
C	-7.029479952619	-4.047650334496	-5.765942002646
N	-6.773276085131	-1.955772297861	-4.940565683563
C	-8.319926109781	-3.450558833258	-5.584826112837
C	-8.144795484116	-2.164963608350	-5.073004870533
H	-9.292595986371	-3.913979284633	-5.812142382807
C	-9.170187809103	-1.130069355376	-4.710796775929
H	-9.042580393017	-0.174116438046	-5.273897990805
H	-10.182602553314	-1.515516032872	-4.956195328485
H	-9.168948425276	-0.872532705052	-3.624200740121
C	-6.164889620364	-0.723271442248	-4.450706934328
H	-5.161788454527	-0.615143542214	-4.923977196308
H	-6.764724205806	0.140403581125	-4.821396226545
C	-6.028342575321	-0.638386139027	-2.917941405391
H	-7.042231372247	-0.607780481199	-2.457002131889
H	-5.551244125037	-1.572214326952	-2.537989422786
C	-5.189610385762	0.571962289549	-2.470507253762
H	-5.560657288663	1.499761193408	-2.974330813039
H	-4.141120365331	0.431893036675	-2.831616439150

C	-5.183146726984	0.792809703089	-0.945350466633
H	-4.337554266584	1.471006810553	-0.672723959814
H	-4.961193108289	-0.176917284182	-0.438140499339
C	-6.495578281728	1.380065515462	-0.399403432976
H	-6.657604262579	2.393318493039	-0.838844351223
H	-7.364530361020	0.761950172670	-0.712966337409
C	-6.501834535434	1.484913628891	1.137094546399
H	-6.496121072591	0.469105848125	1.591186121840
H	-5.558585280711	1.958791938066	1.490058797626
C	3.011715417400	0.976368012135	-5.010980281809
C	0.853248914824	1.687991498389	-4.778311112811
C	0.859985586929	0.679234264293	-5.755140123262
H	2.582664921228	-0.537031877043	-6.578368839719
C	2.215152654812	0.243212473061	-5.894069413655
N	2.167176357757	1.847713099496	-4.321186443455
C	2.591565512606	2.815489527770	-3.316150963881
H	3.650104712773	2.590459261074	-3.052220385853
H	2.018325736723	2.664668581954	-2.375016690124
C	-2.231891127551	5.370407128304	0.483518039447
C	-4.131512197304	5.364299335230	1.759267467227
C	-3.897798716085	6.686080787320	1.353804678959
H	-2.240574753803	7.549162827957	0.066727958398
C	-2.703647405987	6.678607563000	0.557049835712
N	-3.112499020991	4.567547200680	1.212725608014
C	-2.802083283476	3.194947474670	1.606506213593
H	-2.568539852606	2.578926465697	0.708843964298
C	2.493313109369	4.273157903000	-3.749942287239
C	2.254483191794	5.274528757867	-2.780666515188
C	2.654347181470	4.653598005709	-5.100089541869
C	2.178296026689	6.624441823861	-3.149825165375
H	2.119783747708	4.987998803230	-1.720737407962
C	2.576774800534	6.003909752541	-5.474986425265
H	2.829431745959	3.878539838238	-5.867995482952
C	2.336542013047	7.002068681946	-4.502575245260
H	1.989069471281	7.410636539776	-2.398037912207
H	2.702523629433	6.290572313881	-6.532560939719
C	3.082049875041	-4.121002054909	1.111695358090
C	2.217317159858	-3.007590725927	1.073112363174
C	3.950193535525	-4.350070746978	0.020937276903
C	2.209830024197	-2.135269440860	-0.035042735204
H	1.526904210168	-2.795583707864	1.910434646719
C	3.937281201311	-3.490638593681	-1.094362003071
H	4.653405877604	-5.201587329220	0.049416028237
C	3.066749241008	-2.390243813214	-1.129510576849
H	4.623020636534	-3.674837167207	-1.940877986982
H	3.039693061986	-1.725677550862	-2.005431472471
C	-2.826077569878	-3.972295574033	-0.233362684763
C	-2.997686510525	-4.476505375342	1.075667654165
C	-2.716987219163	-2.577347310777	-0.418332745127
C	-3.087139450038	-3.608875731716	2.170547444155
H	-3.041804722364	-5.568662338772	1.239682259818
C	-2.804132291291	-1.703289873114	0.677586042139
H	-2.538128757268	-2.172511817809	-1.431079665143
C	-3.004957584121	-2.210573385137	1.981088450435
H	-3.212640984752	-3.999071777684	3.195463371226
H	-2.696157193858	-0.617407334414	0.524982934613

H	-3.723616471674	2.739704978173	2.032243589547
C	-1.660639204506	3.073436118508	2.610967627833
C	-1.528848998888	3.984371904465	3.684434926405
C	-0.722371752277	2.027093764796	2.480009672096
C	-0.478542467928	3.848476525446	4.604776108105
H	-2.247237503719	4.818506040487	3.788656741513
C	0.336523303352	1.898767721063	3.390563137193
H	-0.791954629084	1.302913322566	1.650263255872
C	0.471802996858	2.812234915190	4.459491390492
H	-0.365079671166	4.556436605942	5.444560532532
H	1.066110326427	1.083116849019	3.257820461376
C	2.232336043784	8.457976854897	-4.844627475840
C	1.291141416085	-0.943253854278	-0.007637065482
C	-3.098385718846	-1.332359819289	3.190136533297
C	1.616810682821	2.728618033887	5.431083246338
O	2.406953438089	8.700871886723	-6.176981694939
H	2.319570973929	9.676642658992	-6.284292981967
O	1.451753748338	-0.136321028655	-1.077896734595
H	0.851854260739	0.669818695446	-0.992376798137
O	2.551352452824	1.837586057828	5.038727831140
H	3.335359754246	1.868764891111	5.666746021171
O	-3.231584456466	-0.005720034807	2.876658912883
H	-3.183918946531	0.481898513284	3.732683152827
O	2.013202225451	9.348626021556	-4.037122287074
O	1.704567831155	3.402222900477	6.453523496446
O	-3.074399377810	-1.729316878833	4.344824357758
O	0.494752361318	-0.726642800172	0.901025461916
C	-6.749266737168	-5.437876071586	-6.273388607146
H	-5.723409079287	-5.531531517979	-6.695573463834
H	-6.839952409636	-6.210666998227	-5.472251597886
H	-7.463123167191	-5.728589351054	-7.076912314193
C	1.020502665753	-3.324324914390	-4.355742808051
H	1.178789522817	-2.219406489437	-4.357870734683
H	1.829874523204	-3.768975603008	-3.736611942379
H	1.179529089964	-3.670684637774	-5.402951725941
C	7.658583053851	-7.159513250070	2.624460666580
H	7.865626909841	-7.683540456620	3.588000832490
H	8.109844609706	-7.781893147051	1.819159337607
H	8.219908306135	-6.199363543305	2.654328300876
C	9.278663962953	-1.999913260437	7.643870506852
H	8.957117368335	-1.872839595735	8.706013632182
H	9.980723007402	-2.862574102120	7.625643228295
H	9.868782609680	-1.091827295915	7.379951262530
C	-10.973592063731	2.761605618441	3.248096956096
H	-11.808172257686	2.757047045939	2.505108858424
H	-11.104515449695	1.859175211547	3.888141999896
H	-11.139720104131	3.647616343324	3.900136612931
C	-4.767974726707	7.881010611707	1.641371041136
H	-5.453246189629	8.121933325534	0.793461191884
H	-5.405812866414	7.727723130848	2.540137003653
H	-4.154944550994	8.792257628665	1.823727748397
C	-0.335627658115	0.139904425085	-6.494755061741
H	-0.897099915829	-0.613390323689	-5.891336311382
H	-1.063126200870	0.938635152639	-6.768837354769
H	-0.030366487462	-0.362985951290	-7.438151194731
C	6.752895703008	-2.140109955068	-5.269391786609

H	6.930526803409	-1.574749930970	-6.216056929907
H	7.377589924492	-3.059484339831	-5.326686044264
H	5.687080985883	-2.464209283588	-5.285910285435

$[Cu(H_2O)^5]^{2+}$

Cu	0.062351457602	-0.044061602990	0.068621530372
O	1.550658763260	0.776269316751	-1.120923676694
H	2.486927982390	0.528974794413	-0.924777227407
H	1.500637778238	0.917989600679	-2.096803135172
O	-0.154539243163	1.847634352773	0.829496376553
H	0.366886719758	2.563644501494	0.392219807813
H	-1.009201861148	2.257229124433	1.102998650918
O	1.410428208584	-0.670950350150	1.700261374537
H	1.822637545075	-1.554413852959	1.827496172707
H	1.716126297099	-0.133149956274	2.464432194785
O	0.152717957215	-1.878618210568	-0.847627502975
H	0.492402581975	-2.045355147829	-1.758550230738
H	-0.626286083994	-2.474402602116	-0.730123014346
O	-1.688828584661	-0.736097992615	0.938766204272
H	-1.680933235647	-0.932661090511	1.906814446316
H	-2.603157766584	-0.430441300531	0.725947711059

$[Cu(H_2O)_6]^{2+}$

Cu	-0.004419254942	-0.087305738948	-0.007205583753
O	-0.234492572380	-0.369019441966	-2.073502817194
H	-1.128594728865	-0.026131723099	-2.315504404877
H	0.381662301241	0.223824649450	-2.566474540137
O	0.448033000614	1.916539081236	-0.290148352074
H	-0.125363568632	2.277303236337	-1.008934768631
H	0.180445875122	2.435758639872	0.506442262359
O	2.297109069385	-0.604068053105	-0.121684567149
H	2.776859747395	0.001317222618	0.487382855693
H	2.400606986739	-1.478781901304	0.316246888750
O	-0.440851618248	-2.099514586486	0.257303475386
H	-0.074115456395	-2.620117383288	-0.497202308331
H	0.054657322388	-2.444445968599	1.038512118337
O	0.179608284259	0.092896996099	2.072957796938
H	-0.263317104046	0.916673130089	2.388238832333
H	1.132818666628	0.243784412350	2.281633884710
O	-2.303658061328	0.470347090625	0.126076024955
H	-2.402335173726	1.355507295890	-0.291304692578
H	-2.806082086210	-0.114182326770	-0.484931720737

3-H_2O

O	-0.364120290237	3.112901996930	1.372424705754
H	0.491435983906	2.680305464626	1.170560254402
H	-0.937417610391	2.830683071413	0.601323083671
O	-0.630108490041	5.455714600477	-0.142324294796
H	-0.353768064902	4.785690384454	0.548754685990
H	-1.286224246934	6.000250971992	0.339865740207
O	-1.797847589720	3.065389043615	-1.032870354689
H	-2.758902864181	3.164960239583	-0.872566479957

H	-1.461927177502	4.007392316909	-0.986812730581

4-H_2O

O	0.898046302826	3.652364809909	1.303762346649
H	0.829791349310	3.752673108264	2.274931105432
H	0.386854511134	2.800427878531	1.122055576013
O	-0.754933465720	5.350755453764	-0.091430523344
H	-0.066646893411	4.824720325577	0.427853701659
H	-1.209827569289	5.874380842254	0.599402748547
O	-2.289985835519	3.201296678277	-0.846572518549
H	-2.082160295913	3.112979877502	-1.799488997528
H	-1.810199904651	4.052951999434	-0.593069048369
O	-0.743421150121	1.532536996297	0.685835081345
H	-1.359084838064	1.289962655234	1.407325961549
H	-1.338744660582	2.047296464956	0.052152426595

A2P-Cu(II)

C	7.728473099704	0.821319208015	-2.292070595695
C	8.740389986630	1.483041801821	-3.023277315743
C	9.739401523789	1.867590163729	-2.084933752407
C	9.337447045476	1.452667819925	-0.807582342796
N	8.104680175831	0.812332967319	-0.946485488257
H	10.667232730845	2.416936827871	-2.305222538815
C	10.021829647828	1.669289913245	0.507223066647
H	10.337722937772	0.723222036488	1.004371204278
H	10.935782110524	2.278690247392	0.350281245300
H	9.379215704548	2.221517304195	1.233133553587
C	7.406196393637	0.071629710407	0.106829138777
H	7.729958695105	0.481712099867	1.089088327384
H	6.315051875317	0.287766643807	0.034360292658
C	7.645906231340	-1.455892505001	0.062074086648
H	6.977818641764	-1.929678516452	0.822382913664
H	7.306472889795	-1.841826353017	-0.928923843720
C	9.105176801811	-1.870207690238	0.321081651745
H	9.774853057771	-1.316067899754	-0.379649165744
H	9.393763358110	-1.541160792786	1.347785422941
C	9.402404555473	-3.376440404359	0.186450360478
H	10.443128094133	-3.553959642566	0.547698807847
H	8.745966179748	-3.960811012359	0.877482831671
C	9.281927846377	-3.935438301038	-1.245382115071
H	8.213244718308	-3.991533541634	-1.566452637967
H	9.775713519219	-3.236178601871	-1.962847069444
C	9.930627819107	-5.328103306341	-1.370471917128
H	9.462784932037	-6.037216990118	-0.651587396209
H	11.006701818567	-5.293546122165	-1.090779319990
C	6.372482123135	0.396882455039	-2.772146417114
H	5.990804888163	-0.489552196526	-2.214439739629
H	6.442815071887	0.076888117438	-3.836131234964
C	5.330457913553	1.565476306822	-2.674734486357
H	5.252525816129	1.905241486397	-1.617167707453
H	5.743341854423	2.432412306306	-3.236298374496
C	0.536853749140	-0.125571610464	-2.968714352492
H	0.301166985527	0.019438995221	-1.888373161521

H	-0.279377129249	0.380865013845	-3.530889638689
C	0.464145762205	-1.684982978794	-3.278372553486
H	1.121388623310	-2.221434824902	-2.551003451343
H	0.854083018303	-1.865756643505	-4.302534863096
C	-0.941618761785	-2.184340888697	-3.154259993737
C	-1.790862346619	-2.459852869797	-4.361592632513
H	-2.553013660362	-3.219808592400	-4.082337115962
H	-1.189277992114	-2.864277888693	-5.203668848780
C	-2.548135630807	-1.145629205089	-4.854302978263
H	-2.790191021822	-0.524769648861	-3.964106500820
H	-1.850932825187	-0.541608547721	-5.475230625456
O	-1.518600408698	-2.242863381979	-2.022507977074
C	-7.185797207156	-2.955257831189	-5.877575326397
H	-7.203206678921	-3.939702284956	-6.403270271937
H	-7.337419033536	-3.193640263618	-4.803101812804
C	-8.411619300549	-2.132727672458	-6.393293946596
H	-8.261135362970	-1.916863996135	-7.473790550266
H	-8.429310443520	-1.144576257325	-5.883120229372
C	-9.736122938727	-2.827275345878	-6.261145859812
C	-10.412405788838	-3.563371614908	-7.245221187783
N	-10.539685573872	-2.814447236980	-5.115421433002
C	-11.658877274490	-3.989130031810	-6.714590909286
H	-10.038566014729	-3.745559830609	-8.264578963221
C	-11.725710013254	-3.499617620252	-5.388666084955
C	-12.862388204827	-3.646428573592	-4.416701034779
H	-13.812846495889	-3.387993401590	-4.940810263285
H	-12.774810843345	-2.915321354371	-3.584387435800
C	-13.011303661510	-5.072683271503	-3.815685090516
H	-13.575036276298	-5.746370137764	-4.499391179673
H	-12.002707727381	-5.546635745744	-3.708944825762
C	-13.612191982732	-5.103668216643	-2.399776444481
C	-14.488869551849	-6.294569754412	-2.056759218110
H	-14.729416207529	-6.311360227452	-0.973940989235
H	-15.438501087852	-6.234646900638	-2.639243225421
H	-14.003210031544	-7.250790521455	-2.359032833261
O	-13.355282555668	-4.222654501010	-1.584590129567
N	9.840288024678	-5.909300416555	-2.714252282003
C	10.746583763665	-5.683102018595	-3.746955622653
C	8.813472227118	-6.733765415161	-3.174524303243
C	10.285369143279	-6.381087809682	-4.894396372388
C	9.082409372965	-7.032687165032	-4.521822251798
H	8.464494858477	-7.673925262901	-5.169109960715
C	11.987774468049	-4.837795643684	-3.637308298352
H	12.003639004639	-4.142065863073	-4.509716771890
H	11.961638660009	-4.191207713207	-2.733519518043
C	13.318490744603	-5.627271816163	-3.649824346769
H	13.358111917038	-6.346809342152	-4.501073158753
H	14.168903339825	-4.924921413732	-3.838730957537
C	13.654137623894	-6.367227390941	-2.348644044490
C	14.799555742271	-7.364720249471	-2.425672831772
H	15.122127608670	-7.664021056094	-1.407666519520
H	14.456475605314	-8.274106691659	-2.973425613500
H	15.663246460205	-6.959361218831	-2.999102005974
O	13.049515007772	-6.155765105171	-1.303270609616
C	7.651011192996	-7.192027159493	-2.342541090157
H	7.726598721116	-6.816561574097	-1.298619910571

H	7.671429505587	-8.304506807934	-2.259186112813
C	6.262242511952	-6.778820020334	-2.932684851027
H	6.240182024370	-5.673390211244	-3.058326669817
H	6.180799783170	-7.190560505215	-3.965012122677
C	5.102111943317	-7.281563516892	-2.120515570428
C	4.615508222385	-8.598919660295	-2.055386765171
N	4.415784751753	-6.519092254578	-1.167444846413
C	3.611473960100	-8.623138387661	-1.040858981364
C	3.497326642479	-7.340323557663	-0.501718314373
H	3.029554704572	-9.501959907666	-0.722056498262
C	4.544966905817	-5.078979156974	-0.971875483246
H	4.389977464168	-4.842096910595	0.104856550972
H	5.595983433538	-4.781623538793	-1.185055914417
C	2.537300499970	-6.843187271091	0.533845683352
H	2.375733939212	-7.643856729034	1.290020205222
H	2.960374537333	-5.976481244898	1.091516573245
C	1.133522464484	-6.428229257373	-0.052366348748
H	1.292241508551	-5.848770772053	-0.990652878258
H	0.556306608935	-7.338533950521	-0.323682129533
C	0.366950031317	-5.531357299623	0.895868542047
C	-0.910939040577	-6.061151633990	1.507808416367
C	-1.580284533726	-5.217204548679	2.615549155440
H	-1.526488281830	-4.143644450285	2.331966649066
H	-0.969123356852	-5.306206011575	3.546030351578
C	-3.005632753465	-5.615847732723	2.902478288589
C	-3.478048557905	-6.792884374003	3.492657035684
N	-4.116114364974	-4.809074000088	2.600973528861
C	-4.906496678392	-6.689579763929	3.543449702530
C	-5.286840305879	-5.463632434351	2.998562654104
H	-5.584987599929	-7.444533930094	3.966174088263
C	-4.056500834404	-3.497977411163	1.977121785621
H	-3.240666031426	-2.892861814907	2.437766769952
H	-4.996649756375	-2.942950533577	2.188758440393
H	-1.613205303226	-6.216743039913	0.647123659944
H	-0.716201203256	-7.104678225326	1.856533187189
O	0.819460283810	-4.392676432302	1.134470725992
C	-6.653606570165	-4.832174849889	2.901873332093
H	-6.702743986371	-3.912314591784	3.537139438986
H	-6.864181358094	-4.481733045086	1.863411080555
C	-7.792381316425	-5.788552754691	3.343982114359
H	-7.565757226045	-6.147129555127	4.373911847191
H	-7.773806012465	-6.699394368374	2.700069368029
C	-9.162652174504	-5.177468164940	3.377570200974
C	-9.797456647945	-4.548317368430	4.476405510701
N	-10.063705211949	-5.189217572189	2.311819064949
C	-11.101887744626	-4.186873458620	4.045883823818
C	-11.260930118011	-4.591960246868	2.712054508891
H	-11.878901587173	-3.697675100347	4.652451821567
C	-12.476465240210	-4.520695682848	1.842476186431
H	-12.882547474950	-5.535117029036	1.609553702060
H	-13.278797120197	-3.964555869843	2.369722185272
H	-12.313074275050	-4.018159992769	0.862317683940
C	-9.802867585397	-5.670489481129	0.955291533718
H	-9.003314649902	-6.443909227916	0.999169372908
H	-10.715436864701	-6.195414457181	0.589243418435
C	-9.422509955961	-4.554595753873	-0.043950094715

H	-10.205617788354	-3.764491932814	0.001236009085
H	-8.473725158748	-4.065577129102	0.285548555371
C	-9.286536227535	-5.077597282327	-1.485623526329
H	-10.215498344876	-5.642539173532	-1.746078901085
H	-8.460522727182	-5.827928035780	-1.536938982058
C	-9.059141830018	-3.995074572581	-2.564528672666
H	-9.020541336195	-4.503683276769	-3.557090840977
H	-8.053485039717	-3.525186981296	-2.423064608951
C	-10.146907017172	-2.902282354117	-2.587823336741
H	-11.135240209381	-3.358302910827	-2.352714702666
H	-9.972952086657	-2.170292284680	-1.764275507811
C	-10.249551903093	-2.064587068242	-3.881470239472
H	-11.039380616418	-1.288709710009	-3.741506598456
H	-9.302051103367	-1.506611599146	-4.040490201020
C	-3.820203712881	-3.538466017256	0.446648986947
H	-2.949573212537	-4.218635889332	0.246681929521
C	3.546077497345	-4.203634740106	-1.819261365819
H	2.842344425089	-4.920681936381	-2.322745830334
C	4.251733001396	-3.382792846813	-2.901097691678
H	3.525453100883	-2.776242707863	-3.484539890181
H	4.791664769479	-4.042836628132	-3.613765820455
C	3.970108887053	1.267308456496	-3.240098186460
C	1.822416331616	0.517680329088	-3.367021536010
C	2.133630431420	1.104367745203	-4.608973454567
H	4.042788858574	2.092974609530	-5.302159401631
C	3.478487347328	1.567866537214	-4.516373828409
N	2.956725774580	0.598990432539	-2.549896110940
C	3.000421439744	0.230092250464	-1.134240989799
H	4.056457762475	0.011073545680	-0.861274380726
H	2.455367700264	-0.728539698173	-0.984002939892
C	2.449423525199	1.327176199281	-0.181514078536
H	1.359866439741	1.478458086112	-0.422208194461
C	2.564670756302	0.883000067048	1.279167716234
H	2.188843904441	1.682903732303	1.952545781627
H	1.975346791313	-0.041977631604	1.462762673167
C	-3.766023852100	-1.463305634511	-5.661540545362
C	-5.836314627889	-2.326300879894	-6.108735081954
C	-5.160700145455	-2.180873786556	-7.335104064841
H	-3.072065113617	-1.439468954661	-7.772659685189
C	-3.870099039538	-1.647105419751	-7.042898890509
N	-4.978405799753	-1.876097452224	-5.091993585602
C	-5.320871183892	-1.773224436620	-3.676803371635
H	-6.030196583138	-2.593426776886	-3.429534884308
H	-4.405964525629	-1.960194286834	-3.067884141111
C	-5.967442281308	-0.424124031827	-3.228369081954
H	-6.304542611976	-0.598652193700	-2.175879960193
C	-5.020047999243	0.784736310137	-3.233132460458
H	-4.633751798903	1.000341616117	-4.256435385288
H	-5.562671917485	1.686814993407	-2.877150728344
H	-4.146950833672	0.629747185792	-2.558874899239
H	3.626864817287	0.685733849249	1.549762483755
H	4.989116051011	-2.682704567362	-2.446485555169
C	-5.017219868325	-4.058293010284	-0.352333138736
H	-5.909768247044	-3.406417367335	-0.215629960893
H	-4.782539665387	-4.109556254375	-1.440157614078
H	-5.283134558324	-5.089150526181	-0.029533885238

O	-7.159774677161	-0.146969087361	-3.958962985753
H	-6.897264954923	0.150424691970	-4.857308190301
O	3.155316369514	2.557800458445	-0.315936188887
H	2.985975752628	2.912713924927	-1.214525859607
O	2.816818284596	-3.273579470849	-1.000626706591
H	2.324869684626	-3.760598287440	-0.298028330301
O	-3.466977237486	-2.183277237439	0.073418881904
H	-3.532375606225	-2.125522912425	-0.905289017049
C	8.729104484744	1.777825353339	-4.498163993554
H	8.264325759533	0.957427657230	-5.090263752718
H	8.163555810530	2.710202724748	-4.737163251760
H	9.760801830538	1.924212096935	-4.884730262844
C	1.226318611064	1.210634303730	-5.805626052532
H	1.204529045527	0.271786999255	-6.409562253483
H	0.173181248110	1.439543248774	-5.520824837801
H	1.559939461968	2.020324730622	-6.489516482818
C	-5.687945576251	-2.546468238174	-8.697322331983
H	-6.357704472981	-3.434970979777	-8.662851222125
H	-6.272603654058	-1.717587541373	-9.161415999428
H	-4.858574882256	-2.784037399458	-9.398142924862
C	-12.716708790548	-4.791564029847	-7.420442652835
H	-12.652303054553	-5.879299261506	-7.179226064838
H	-13.745398609438	-4.461783526476	-7.149847086130
H	-12.619111554993	-4.700994893387	-8.523663096807
C	10.953765249461	-6.424607737324	-6.240510156489
H	11.774880754463	-7.179484656892	-6.281338734431
H	10.229860093143	-6.698636207830	-7.037846716043
H	11.404797036878	-5.445531094183	-6.520127604527
C	5.097835397039	-9.772914280569	-2.864908058916
H	5.448252973948	-9.471300638749	-3.877322536723
H	5.946944260210	-10.304516805222	-2.373328507349
H	4.290587176891	-10.524526070786	-3.005079520729
C	-2.651080884655	-7.949124506920	3.990550729632
H	-2.531041831338	-8.749865736833	3.222097948010
H	-1.629060383587	-7.635904560833	4.302672779075
H	-3.124498444254	-8.430472944136	4.874157263272
C	-9.201284574913	-4.332458365814	5.840419234759
H	-8.755930730218	-5.265141070378	6.258196026846
H	-8.391166318930	-3.565328609632	5.834683710726
H	-9.971842200372	-3.985345803813	6.560916693598
Cu	-0.975027737711	-1.949516196502	-0.221277735140
O	-0.090106922399	-1.937962789592	1.526182103706
H	0.283757286500	-2.867355317750	1.672742091045
H	-0.547750710157	-1.663234920236	2.349188903942

BA-Cu(II)

C	5.411757292665	2.169560314471	-1.968578252392
C	5.032226020208	2.560506756553	-3.262348697554
C	6.238531891026	2.777757721168	-4.004563693763
C	7.324197257197	2.518493326531	-3.167172210691
N	6.810546628213	2.157105551362	-1.923025589139
H	6.309353907429	3.104287275548	-5.053422150303
C	8.793306529802	2.588742678835	-3.461668737589
H	9.333316455142	3.300107865009	-2.793281745495
H	9.307831234054	1.602313561561	-3.374021074434

H	8.945315153689	2.943540811836	-4.502410415540
C	7.619419607471	1.666894908347	-0.807534188444
H	8.602136495324	2.189609972991	-0.832027570621
H	7.142018398235	1.973090050592	0.151291375900
C	7.836008669906	0.140549001961	-0.831894603569
H	6.844510491995	-0.370639948558	-0.761873141455
H	8.249891988742	-0.137619683518	-1.829401962254
C	8.762984004999	-0.359467956764	0.290138513469
H	9.779115322421	0.085710128369	0.156133957731
H	8.395683984436	0.026396438102	1.271728487891
C	8.881470250626	-1.894385716070	0.380004751940
H	9.505130473866	-2.155801440008	1.267420199897
H	7.871312124299	-2.328199726003	0.587070423472
C	9.473752434326	-2.574190763223	-0.867954852271
H	8.844137369579	-2.359701977180	-1.764716793328
H	10.480143748114	-2.146594179610	-1.094671500859
C	9.589702403068	-4.100478617401	-0.692438828827
H	8.600832750606	-4.527406981931	-0.410414656907
H	10.272469729068	-4.367509521110	0.143525832708
C	4.544825907929	1.984224454999	-0.753898478415
H	4.802296827275	1.046837049247	-0.201265421700
H	3.499581150096	1.841712529699	-1.107463676629
C	4.571654621617	3.213035611649	0.236201374370
H	5.286045812305	3.012781772287	1.068962625531
H	4.997312008798	4.082244555463	-0.311048882709
C	0.224033702874	3.232482260659	2.928052114402
H	0.420009490326	2.181248369541	3.231472476832
H	0.254799807996	3.818379249970	3.878666010915
C	-1.210548247888	3.323920084398	2.366166077843
H	-1.534892331806	4.379136251684	2.234119310088
H	-1.937612710246	2.910983808667	3.113477980888
C	-1.445316182191	2.574003865826	1.068933223402
C	-2.587229051514	3.004721485747	0.167864851394
H	-2.156415928136	3.190428796653	-0.845721005464
H	-3.023446672801	3.966215009741	0.516345723704
C	-3.696639093401	1.929339879196	0.065367606125
H	-3.234050953311	0.967429480668	-0.264059789648
H	-4.099907397113	1.731795802370	1.088243530959
O	-0.722945565071	1.595920038544	0.768471684581
C	-8.127863208614	1.654291383785	-2.395537178142
H	-8.278662739870	1.908320773197	-3.468439133714
H	-8.176211539877	0.543341118430	-2.344740414592
C	-9.336153666095	2.263774965610	-1.592881139359
H	-9.169960035517	3.362909279838	-1.529489231548
H	-9.308139001322	1.896452220597	-0.542579083728
C	-10.683116524200	2.049846886988	-2.220024061234
C	-11.238198512643	2.798817453998	-3.270759165680
N	-11.627199589415	1.085688202294	-1.853393013040
C	-12.539579830725	2.309674334330	-3.542845252022
H	-10.745418120162	3.647256731185	-3.770369993810
C	-12.768153446470	1.233027508353	-2.644908474874
C	-14.041636173678	0.457310040085	-2.415641956057
H	-14.782540002210	1.130954708980	-1.915813975065
H	-13.869462622439	-0.373933590326	-1.699807081544
C	-14.724217586361	-0.112245910796	-3.677887740935
H	-15.697948931982	-0.579391117152	-3.385505263618

H	-15.015286606922	0.689806925729	-4.392792714169
C	-13.940649795753	-1.191844034019	-4.432120697547
C	-14.440520401820	-1.519834671504	-5.831392517261
H	-13.965028707140	-2.448837718112	-6.206678850044
H	-15.549138987478	-1.617098141236	-5.861077373044
H	-14.180569473970	-0.681243620615	-6.519909627145
O	-12.976134937828	-1.763797332689	-3.936914966090
N	10.058311956375	-4.798841797408	-1.893954938697
C	11.386950879676	-5.072202595741	-2.202807798733
C	9.238945490144	-5.259288214099	-2.926669125728
C	11.416722949447	-5.727342518693	-3.463894105462
C	10.072746239008	-5.838264347225	-3.900211398885
H	9.732423373193	-6.296214582465	-4.841392950599
C	12.564009055489	-4.702210901788	-1.340531997010
H	13.311928291422	-4.187427052204	-1.989479364840
H	12.276309288414	-3.962753842613	-0.562202499071
C	13.281443623980	-5.893933825519	-0.663602392075
H	13.532802356117	-6.690940033499	-1.402056598969
H	14.272789879673	-5.557973211249	-0.268057571737
C	12.536271082665	-6.520059726401	0.521549255909
C	13.071373243261	-7.855421262608	1.015163881961
H	12.628722534983	-8.110264391058	1.999551573295
H	12.799162916634	-8.652122657607	0.283153201144
H	14.182573774500	-7.856253302430	1.081512268879
O	11.575343030179	-5.972411401336	1.050885050609
C	7.746537110455	-5.104104035372	-2.952532019192
H	7.390457192003	-4.507919354868	-2.083977034146
H	7.254913381670	-6.101314530247	-2.851051048172
C	7.223555351573	-4.435600826501	-4.271310652578
H	7.821852693538	-3.520434739845	-4.466804319380
H	7.442179891168	-5.117181975976	-5.124796792536
C	5.752525688684	-4.142989192961	-4.248623978381
C	4.691618752226	-5.049651174687	-4.433768328363
N	5.203757174085	-2.899703943966	-3.918033014023
C	3.482183120696	-4.323564113765	-4.217546586642
C	3.814110404484	-2.999992317845	-3.900686980090
H	2.467521697603	-4.741972989361	-4.292810776194
C	5.930395875441	-1.632494665665	-3.768750318328
H	5.423498253136	-1.038785411966	-2.975000609097
H	6.944862882625	-1.856017897983	-3.370943533770
C	2.923407129047	-1.833711016555	-3.555382188619
H	3.225078953208	-1.384985944484	-2.576714684484
H	3.033479037899	-1.000915202048	-4.293234657133
C	1.449791463064	-2.247103150969	-3.477020932750
H	1.074208289771	-2.611575151130	-4.464075071354
H	1.339642740775	-3.139907114716	-2.808023242998
C	0.478705820051	-1.214874531022	-2.940013603949
C	-0.995256736555	-1.567401830471	-3.053146408421
C	-1.970393790836	-0.773175314333	-2.150283967398
H	-2.070960955494	0.265150094860	-2.540865009950
H	-1.516423390766	-0.675201823003	-1.134383731666
C	-3.344723414982	-1.375692616681	-2.062053943495
C	-4.476827667684	-1.104201461702	-2.847203625334
N	-3.671936656422	-2.401061175472	-1.165172464362
C	-5.509193299534	-1.980910585125	-2.390519266762
C	-5.005559878851	-2.763162258712	-1.344284440045

H	-6.533883011648	-2.036615563483	-2.790996753107
C	-2.789902057720	-2.889675884213	-0.105045027164
H	-1.746070377616	-2.909293633032	-0.494583144596
H	-3.044288988242	-3.951858358751	0.110951606886
H	-1.274336694140	-1.431566736712	-4.128576069715
H	-1.103453192304	-2.666518784052	-2.891517697106
O	0.883251817453	-0.157464427103	-2.420565869148
C	-5.718265568658	-3.824863939904	-0.553863572159
H	-5.193996184502	-4.057636926778	0.399834827768
H	-6.708911442787	-3.422406205264	-0.243788361255
C	-5.955258958036	-5.157853411166	-1.347431153092
H	-4.973215826268	-5.668740166225	-1.480607294291
H	-6.292068417901	-4.895286741746	-2.375079919481
C	-6.916660220427	-6.099653773107	-0.687409815755
C	-6.670449338339	-7.010301422260	0.366399653532
N	-8.275724685913	-6.168955921760	-1.004577126000
C	-7.912910429400	-7.629852400397	0.674584940794
C	-8.895396909453	-7.101774712669	-0.176553447607
H	-8.089300642347	-8.404637388763	1.435934907801
C	-10.349562332166	-7.442733844041	-0.273718707757
H	-10.617777794441	-7.894560330321	-1.258539302121
H	-10.613703858925	-8.183648635541	0.508813952383
H	-11.011947194763	-6.556629574530	-0.131471206517
C	-8.959584643663	-5.358015589396	-2.015019361932
H	-8.271056942960	-5.220507894561	-2.880003582550
H	-9.821047053368	-5.941486061997	-2.409006959057
C	-9.429538984475	-3.982851652782	-1.496661716304
H	-10.234602492956	-4.131581989965	-0.738918592064
H	-8.583999423065	-3.507242526339	-0.945724339844
C	-9.889258925161	-3.031985233646	-2.616884515827
H	-10.830823922982	-3.397976504280	-3.090284251421
H	-9.122563542948	-3.041829951883	-3.429931678178
C	-10.084624333738	-1.572488405517	-2.159836538533
H	-10.247141359400	-0.938444117343	-3.061200877194
H	-9.134028085417	-1.213264865614	-1.690722772460
C	-11.252944509851	-1.352641666163	-1.186569232199
H	-12.177182021948	-1.747619614391	-1.662973790353
H	-11.101160349091	-1.934163125090	-0.246006292509
C	-11.469298384961	0.115219689015	-0.759090261588
H	-12.358210807393	0.183850779593	-0.090079252262
H	-10.607686400703	0.456140411833	-0.144370285473
C	-2.843535287188	-2.051364340888	1.188680094282
H	-2.695889127469	-0.976384050613	0.927382688698
H	-3.867688809715	-2.117715671711	1.629955897070
C	-1.790486717916	-2.472225267761	2.228241726565
H	-1.946338999934	-3.540546230035	2.512766922312
H	-0.780349011322	-2.432610955318	1.750981343269
C	-1.801506031894	-1.594667480653	3.489797718382
H	-2.787674087974	-1.632230943764	4.005465287571
H	-1.034090308603	-1.919981307875	4.226550085713
H	-1.604036519412	-0.524403362659	3.243116167482
C	6.018795000640	-0.781331765741	-5.056309847790
H	4.999875735825	-0.720610972136	-5.505687575425
H	6.271650839252	0.260716604920	-4.748121367851
C	7.026203108127	-1.256175044701	-6.123167982848
H	6.816943565841	-0.700755306756	-7.066907648359

H	6.843719876839	-2.328552616661	-6.374848123822
C	8.499616888567	-1.035980896754	-5.740366567504
H	9.185063727305	-1.387836682004	-6.542361367725
H	8.712614561609	0.043764242899	-5.568088917445
H	8.792755485701	-1.577714130937	-4.810311372566
C	3.239484136946	3.667227841657	0.766281286222
C	1.290510262579	3.704248251718	1.965089804387
C	1.289620967058	4.839149013229	1.136482901654
H	2.857766465807	5.561383954331	-0.334710023181
C	2.506186015695	4.793693360043	0.373294174638
N	2.489229074130	3.007020718220	1.735952449609
C	2.981525891825	1.839205755281	2.477660833520
H	3.488476177329	1.165093568078	1.750834525386
H	2.105771049525	1.268872943659	2.860165403023
C	3.948042690543	2.174147928156	3.637394093019
H	4.416201964072	1.214900549981	3.967309947940
H	4.781423056786	2.793399417935	3.232619327839
C	3.326778357592	2.884451188078	4.857568622590
H	4.159134682121	3.276701296838	5.486292872974
H	2.770875334957	3.791629998194	4.519386446476
C	2.428207347691	1.991718975182	5.729015880658
H	2.020281562678	2.555249052075	6.596571036297
H	1.553952897201	1.581461732005	5.173106824343
H	2.994877023777	1.123805860481	6.136650882039
C	-4.798254844181	2.329308586671	-0.878791798057
C	-6.777193582779	2.164599189883	-1.992982718586
C	-6.066275095416	3.243051842083	-2.554631961895
H	-4.035068790676	4.073921871710	-2.048386795043
C	-4.827338419051	3.336381541014	-1.850935357007
N	-5.997987877595	1.627191276762	-0.964685117270
C	-6.367217527958	0.508792069399	-0.095140333097
H	-7.108433299231	-0.112945486688	-0.640030696398
H	-5.477237013242	-0.149600605437	0.029115532886
C	-6.916487484383	0.928033594571	1.285569908897
H	-6.189349172201	1.617143423387	1.775569955201
H	-7.846071952839	1.530913429984	1.149169430561
C	-7.192935293210	-0.265297269242	2.223843250897
H	-6.257069248717	-0.867758858326	2.334427964897
H	-7.409931502392	0.131413116477	3.242667504166
C	-8.347116414755	-1.183341755113	1.789649624531
H	-8.165580086839	-1.663624251298	0.801319610608
H	-8.508790762940	-2.003713562074	2.522886107716
H	-9.304573376805	-0.618452914950	1.713056240294
C	3.632017692137	2.756676042316	-3.786624166185
H	2.948038287838	1.927487560988	-3.484086258437
H	3.186126649517	3.729842419235	-3.459813169879
H	3.623680940135	2.778465529606	-4.897995013958
C	0.269452521506	5.948948076225	1.059478650196
H	-0.606023985024	5.704293918387	0.411603398929
H	-0.126832824392	6.229516820162	2.060704694153
H	0.728979214883	6.865395785763	0.630382835881
C	-6.529005676543	4.130506041653	-3.679300553850
H	-6.961616018396	3.548400926504	-4.525194344573
H	-7.313418145963	4.854541560434	-3.354687626084
H	-5.687760786909	4.729554338304	-4.089386963801
C	-13.495055858861	2.886209266611	-4.553462274580

H	-13.147119100143	3.884969492776	-4.893561911405
H	-13.588635582836	2.252621691632	-5.466888430316
H	-14.520886196610	3.019989495223	-4.140808363824
C	12.642520187395	-6.205430318140	-4.191005727980
H	12.987254168495	-7.203493290967	-3.829142498746
H	12.445564516804	-6.312154628891	-5.279339595732
H	13.502025677050	-5.507364746483	-4.073249033369
C	4.814603639510	-6.511643680141	-4.768087289991
H	5.670837684181	-6.716380586404	-5.449591060814
H	4.967798765205	-7.144217813872	-3.860983316326
H	3.897234399293	-6.888721519137	-5.270250909700
C	-4.579477995153	-0.085551863987	-3.952379052698
H	-5.614873494507	-0.037659666178	-4.351617827219
H	-4.321777666384	0.941411487927	-3.603564855570
H	-3.912244330885	-0.321354359255	-4.814317873842
C	-5.345739183190	-7.257036737421	1.035363146334
H	-4.491753578624	-7.156926473484	0.327354681406
H	-5.158888093972	-6.545641721849	1.875729930605
H	-5.297823686235	-8.279956700483	1.467832416409
Cu	0.376478457567	1.076317948379	-0.857078871873
O	1.075428432656	-0.616648256911	0.551956749879
H	1.966740291725	-1.005416493803	0.678581086290
H	0.755810019653	-0.421316364227	1.459197012631
O	0.943016254926	2.975486497452	-1.570734680907
H	1.545794735678	2.996837571495	-2.347461636050
H	1.377350214722	3.593465912794	-0.910620406445

DAP-Cu(II)

C	15.934222965111	6.939302918442	1.656388335113
C	16.578511261080	8.072377164784	1.089119928375
C	17.942691196869	7.992928196044	1.459886026987
C	18.126114389705	6.840072908794	2.245527639301
N	16.890772335436	6.202742972358	2.355004098300
H	18.736483055403	8.708815387054	1.198651734449
C	19.378303863036	6.361693645157	2.908573003289
H	19.259245701349	6.253890596926	4.012652407619
H	19.729516938653	5.377274924591	2.522064466838
H	20.192437742926	7.095043872381	2.734619183903
C	16.647445496315	4.930374939149	3.051828461115
H	17.433255892922	4.819093981006	3.831878377013
H	15.682996981544	4.998062666561	3.601543141609
C	16.608773278350	3.670584148568	2.162451365130
H	16.271377154907	2.841966680594	2.831134357310
H	15.791504631257	3.784625515919	1.411621755363
C	17.929049053622	3.291590493294	1.464005864379
H	18.173608438612	4.046383337781	0.677978882240
H	18.760748857880	3.342979701445	2.206438305308
C	17.937074400930	1.874312937021	0.852815857915
H	18.952092339056	1.661402894505	0.441532063316
H	17.787672464870	1.132232157344	1.676880213697
C	16.879968056090	1.620027347044	-0.236397294622
H	15.863826834104	1.851106631619	0.156049784572
H	17.034076515732	2.319184927040	-1.092701324841
C	16.893139815826	0.157081167950	-0.734217795733
H	17.004104588700	-0.530308360603	0.133584565262

H	17.764117022964	-0.044093365207	-1.396598858573
C	14.473178426226	6.626126838747	1.644226568934
H	14.293703494852	5.544557812356	1.444733859365
H	14.004367210963	7.175892894248	0.799291611044
C	13.730539792412	7.049414125855	2.972675180062
H	14.134569424121	6.444168353875	3.817037809167
H	14.008242954383	8.102428368942	3.196088800443
C	9.046478127600	5.062366862967	2.880461416946
H	9.383147227199	4.096150531973	3.314345889603
H	8.224587902613	5.421726355910	3.544364808651
C	8.462502177771	4.800160775209	1.450849962348
H	9.261991392999	4.402671206887	0.787451930669
H	8.109777818585	5.767238207702	1.024724838028
C	7.314920821136	3.792488963863	1.493354974036
C	5.936635482249	4.349413601824	1.866514706403
H	5.624166511319	5.049968007125	1.051997984536
H	6.046439414412	5.009841306100	2.760776092833
C	4.872344863796	3.263443549221	2.132944271310
H	4.731614354927	2.665412581083	1.201960093666
H	5.296101395384	2.541619932190	2.866279650093
O	7.492961032434	2.594014598519	1.283355308603
C	0.287680824077	5.512083095097	2.343037017080
H	0.181618184097	6.476248817299	2.894765761674
H	0.376710741232	5.801080941641	1.271773483695
C	-1.006445873395	4.686612281688	2.537154173201
H	-1.144085522042	4.501588087086	3.631711795029
H	-0.848816508124	3.672040681334	2.092951961516
C	-2.229049963563	5.340040397475	1.951315117494
C	-2.359119173701	6.562075907483	1.281589944522
N	-3.502339066962	4.772633314726	2.034845376285
C	-3.735485957940	6.750063972821	0.949550374204
H	-1.546392776976	7.272315746063	1.069067209496
C	-4.429482968516	5.622098815669	1.425761556269
C	-5.893409055928	5.301413202168	1.305489720188
H	-6.490241184985	6.160245528165	1.694142601864
H	-6.162017073184	4.434526627969	1.948497073813
C	-6.368665433049	4.985068683871	-0.140061845148
H	-6.679703759868	5.905420638701	-0.683874918817
H	-5.519125503166	4.568543507200	-0.738271025529
C	-7.467296874535	3.907209617361	-0.213798970014
C	-8.481149922363	4.044840784383	-1.336486789324
H	-9.114404421897	3.137261092106	-1.409499413646
H	-9.132409191807	4.928595506205	-1.137863640634
H	-7.979052062400	4.244370369804	-2.310919416448
O	-7.491224716305	2.973484175119	0.580077053285
N	15.673651234864	-0.239585530134	-1.450919144028
C	15.383273489727	0.062312235688	-2.777153686616
C	14.590408604260	-0.914967236609	-0.881869533377
C	14.081878720682	-0.425606708948	-3.062707877651
C	13.602116785582	-1.034483441141	-1.872802067833
H	12.626641351656	-1.524362616889	-1.732651404242
C	16.325807690046	0.766958667659	-3.717240666308
H	15.770130308582	1.600913907618	-4.208529404205
H	17.163132118281	1.241823215756	-3.162237576506
C	16.912759678666	-0.125691302144	-4.836041098450
H	16.111042055937	-0.667555864765	-5.390583503527

H	17.402619474281	0.517122270646	-5.610139062729
C	17.977553404780	-1.131627128789	-4.378768873780
C	18.348177456036	-2.212346563656	-5.383449248904
H	19.277228633956	-2.730641761348	-5.070037856301
H	17.522121094349	-2.960466740681	-5.435426236155
H	18.465527033956	-1.800102084417	-6.410765607286
O	18.509093577476	-1.063418794921	-3.276747333424
C	14.565373875862	-1.416745167896	0.529193434274
H	15.521314762695	-1.946476224977	0.758977508498
H	13.767879419596	-2.189082910430	0.599088414804
C	14.334241510816	-0.354237485965	1.669198383690
H	14.683383373012	-0.818990791459	2.616861266575
H	15.013506867366	0.511188445104	1.506594316019
C	12.932735075435	0.162050389650	1.817420462978
C	12.288291891392	1.185968974173	1.091904852174
N	11.994769913834	-0.383648214726	2.708028393054
C	10.945177109908	1.251698609789	1.569806471382
C	10.782605055609	0.289990819145	2.573481136401
H	10.143793156510	1.915353414595	1.208224287539
C	12.253872403610	-1.390828751540	3.745208121483
H	11.354093450405	-2.040968576036	3.816476935153
H	13.070685725352	-2.061320398001	3.397460927132
C	9.526862405592	-0.018305172144	3.344782825114
H	9.736325670477	-0.490969972631	4.329548901154
H	9.023241163383	0.944051735322	3.590977288766
C	8.519424519708	-0.880781995339	2.555913469621
H	8.406008211582	-0.484672324694	1.516521818513
H	8.883696328777	-1.929227006019	2.412951206214
C	7.128532500676	-0.933639481529	3.176247058875
C	6.012956697402	-1.580587965780	2.336612643227
C	5.014928757488	-0.467163442709	1.901676986261
H	5.554979792064	0.244916217934	1.235249931539
H	4.757657640237	0.115332560286	2.812594278618
C	3.778910518369	-0.955177153637	1.201217660735
C	3.612968346872	-1.264228968138	-0.154308997935
N	2.580971207913	-1.248966792173	1.870550728499
C	2.251669488736	-1.750847482101	-0.315795421203
C	1.636222676028	-1.731164122166	0.972187640144
H	1.908784836983	-2.370600876955	-1.163398141484
C	2.345100742448	-1.046786551423	3.305834729109
H	3.193369818226	-1.497864024354	3.869067263131
H	1.447445587305	-1.642727713690	3.584042852681
H	6.415034862620	-2.116429349960	1.447909063558
H	5.492423940524	-2.330777119342	2.975694278374
O	6.864945524399	-0.421219033913	4.257828626992
C	0.315353956551	-2.329388821407	1.396154999900
H	0.489560757187	-3.086952918489	2.198440582270
H	-0.344925777983	-1.557758332748	1.860026483218
C	-0.452268220563	-3.024989938847	0.239296070096
H	0.198411803874	-3.832711627774	-0.172702615133
H	-0.586556052606	-2.291542634859	-0.588869372903
C	-1.772022324591	-3.622987984811	0.639504087878
C	-2.010500577825	-4.815481905446	1.342254515030
N	-3.012829949238	-3.041763328519	0.346312141933
C	-3.428479114180	-4.938571564222	1.478902373305
C	-4.032173630730	-3.840706380834	0.863047849555

H	-3.967717680066	-5.762834369887	1.969806426183
C	-5.486864608450	-3.512460896457	0.708905106860
H	-5.813089441705	-3.484784283855	-0.358194117203
H	-6.097174531253	-4.290470270411	1.212607938289
H	-5.766443963944	-2.532443559598	1.163334714465
C	-3.214095209407	-1.764855433389	-0.326096175137
H	-2.457781722212	-1.662115016170	-1.140560643477
H	-4.202108235891	-1.784892217533	-0.838172406203
C	-3.128070484754	-0.547687100035	0.617504706326
H	-3.987900412955	-0.580820042100	1.325870442398
H	-2.212236659188	-0.649351140876	1.248546187276
C	-3.078875000193	0.799505240709	-0.121928211075
H	-4.008840397767	0.942792784547	-0.724085750704
H	-2.236481160438	0.763513006724	-0.857759398485
C	-2.864925778334	2.014441037081	0.801938999565
H	-2.672877469146	2.919484764822	0.177523744292
H	-1.928432511763	1.849143328356	1.393330530209
C	-4.037405051945	2.302470526410	1.755698645098
H	-4.977077268520	2.448470799726	1.172570663197
H	-4.234763560646	1.419775780458	2.408923684061
C	-3.817019144243	3.504146869602	2.698987119609
H	-4.722474234702	3.645099789801	3.331484549730
H	-2.988067202038	3.281028778441	3.406652144676
C	2.166128689245	0.412526951957	3.806456131380
H	3.118787510809	0.965993913685	3.635691993330
C	1.908268005912	0.390902511818	5.325937440029
H	0.957381338336	-0.137372955272	5.571536740381
H	2.730763491824	-0.117429577926	5.877713070358
C	12.572258460410	-0.799494303262	5.140825968546
H	11.907475102726	0.077785530199	5.313287335671
H	12.296221630489	-1.557722016110	5.913930208735
C	14.036561212849	-0.357496061547	5.381727407351
H	14.391827277533	0.186781772114	4.471740408315
C	14.974265172929	-1.561867631819	5.610706304101
H	14.685851130774	-2.125570060676	6.528183187282
H	14.957456024834	-2.283042862983	4.760564754474
C	12.234947871428	6.978662930528	2.927390321043
C	10.142366703552	6.088851662230	2.901994680978
C	10.003265035368	7.493317178393	2.830101106554
H	11.585241280099	9.103407854255	2.811607181665
C	11.317761404584	8.036071419010	2.842434423250
N	11.502931028111	5.789787435835	2.952317666299
C	12.100449655057	4.459902132938	3.061761847644
H	13.018224934211	4.433035793868	2.435631208651
H	11.412483692490	3.710757603267	2.609439129786
C	12.501122659821	3.996750930331	4.488504309077
H	13.021728956457	4.852888920120	4.987149764692
C	11.286504202628	3.621599646653	5.363660872223
H	11.623135736020	3.289046984559	6.370814044546
H	10.593100904177	4.480498823786	5.514856297439
C	3.572301538495	3.772290168198	2.697407365993
C	1.535530444996	4.810930479488	2.811195319324
C	1.945774265352	4.546696099878	4.127371704829
H	3.846252485754	3.575890532041	4.891300190380
C	3.220284458995	3.894686816994	4.042889224522
N	2.533010081541	4.333370625515	1.946568142650

C	2.463050267906	4.358590529449	0.490415846871
H	2.097911447344	5.356031798707	0.154832578821
H	3.498320886693	4.269925981524	0.088077102457
C	1.575614095032	3.259698843366	-0.168923269190
H	0.519692889044	3.409466811024	0.160646683263
C	1.638936786797	3.400524804966	-1.696491027838
H	2.673297968207	3.234168069919	-2.077669797281
H	0.975232328266	2.659637956976	-2.191952370020
H	10.713580258467	2.779835884021	4.907848402700
H	1.319703159547	4.414266355355	-2.024287027688
H	1.835796114451	1.427598839554	5.722247119510
H	16.031039096053	-1.232873088404	5.732211464932
N	13.488532327523	2.918172089711	4.316557126574
H	13.644841451500	2.423767277566	5.218273377120
H	13.094987567292	2.197870669560	3.684887309111
N	14.057888147544	0.639532023611	6.473751291415
H	13.629533595338	0.242541743682	7.327457779362
H	15.034644310360	0.852620502623	6.734689079218
N	1.140322953358	1.151915725323	3.036563030443
H	0.203773458436	0.741988028582	3.200362939163
H	1.090846001388	2.116335662361	3.416851449742
N	1.959553518375	1.884818420088	0.263704675255
H	2.993963046809	1.808655958762	0.254217601656
H	1.679389220015	1.704544267467	1.273520449275
C	15.920845758700	9.150156338010	0.273146825454
H	15.356451217933	8.735822874795	-0.594042370311
H	15.194886614590	9.749314558717	0.871489936176
H	16.674565838945	9.858716519340	-0.130290307917
C	8.710610244754	8.258638051842	2.747340025376
H	8.315025690464	8.313349247994	1.704517770748
H	7.908991680406	7.804638582400	3.374219113233
H	8.842854591916	9.306655204080	3.092529882282
C	1.210963253981	4.912618268473	5.391654859336
H	0.559193074276	5.803683841813	5.254729609598
H	0.556188368620	4.088704368446	5.763902168695
H	1.921321858493	5.148824053211	6.213999830792
C	-4.335426754333	7.926562289171	0.227685703325
H	-4.390616598152	7.765680987302	-0.875467750408
H	-5.370895931139	8.144389258555	0.573259161950
H	-3.733660490071	8.847658532358	0.387957956853
C	13.360871972364	-0.329950794672	-4.379251096503
H	13.567158755999	0.628877034627	-4.906952656166
H	13.645950833394	-1.150171618491	-5.080790484092
H	12.260568086968	-0.401720166057	-4.237723326383
C	12.892468825978	2.073631641411	0.036164364866
H	13.786595258987	2.621918799758	0.415770584533
H	13.216613921254	1.506524826791	-0.865294833915
H	12.159899993602	2.839211675458	-0.299858614728
C	4.673554455105	-1.218238406012	-1.222322920538
H	5.442940677958	-0.440534535298	-1.018617469523
H	5.213223929723	-2.191233091616	-1.303818323399
H	4.243356347174	-1.008355004629	-2.226060435712
C	-0.973722661418	-5.779287237187	1.855353163218
H	-0.033680511245	-5.749952239553	1.258817399397
H	-0.693782344745	-5.576497822621	2.916938870265
H	-1.347432462992	-6.826279870007	1.823409786623

Cu	1.402142492621	0.155263539191	-0.649259047018
O	0.094847612099	-0.082889327699	-2.367717599243
H	-0.088039691012	-0.948708125009	-2.791595900980
H	-0.660924823750	0.489065932052	-2.621980166118

HAMC-Cu(II)

C	-0.616511695097	4.838484096914	1.573384906998
C	-1.733028137869	4.895047892643	0.724283733998
C	-2.890732249914	4.653667045328	1.543742990266
C	-2.464092755291	4.490871121125	2.870024459073
N	-1.076547464665	4.586323833904	2.874205915786
H	-3.943174019210	4.722377038683	1.224446656250
C	-3.294651189780	4.381339470557	4.115285798523
H	-3.117342579508	3.448359448303	4.697697439201
H	-3.118769337022	5.235185323950	4.811135861661
H	-4.371139907879	4.403554811871	3.844800462816
C	-0.248165307975	4.435767076899	4.076710168158
H	0.759379706644	4.845568257934	3.846903908132
H	-0.665934302507	5.089988343564	4.876143344395
C	-0.119927674885	2.993712070784	4.615410333314
H	0.533598716652	3.043412417173	5.519613869993
H	-1.112034486388	2.653418867219	4.990747287211
C	0.448622135054	1.963493234438	3.624189119141
H	-0.219057304506	1.895755052099	2.732881271764
H	1.429648380572	2.336364202997	3.243152698516
C	0.677069430493	0.558910615092	4.228341000974
H	1.222831610665	-0.071050931432	3.482889482729
H	1.375439126062	0.657996059401	5.092921838779
C	-0.579377952839	-0.196214980757	4.710488365603
H	-0.269043473436	-1.008159995337	5.406276512356
H	-1.222922135749	0.473214417831	5.327929903262
C	-1.448445047344	-0.826831977007	3.595961536532
H	-0.847650626850	-1.541849518575	2.989456738521
H	-1.798317185197	-0.056109923828	2.873601082372
C	0.826680843112	4.981908182749	1.162318086474
H	1.488141368570	5.204226539549	2.029673734908
H	0.920177270011	5.865691704388	0.489429833450
C	1.337873222159	3.726908386087	0.388470381015
H	1.427238432390	2.871668701368	1.096291176611
H	0.525225293302	3.449525002053	-0.321363437682
C	6.263120754207	3.822931367678	-0.963814159567
H	6.534826045655	2.884184754316	-0.432512202504
H	6.640102097685	3.680093744776	-2.000134074942
C	7.090420668422	4.992183559926	-0.355743406677
H	6.848873765658	5.176778842867	0.715695453446
H	6.862656752288	5.937970270836	-0.903233262771
C	8.582819861594	4.690973022309	-0.536831392513
C	9.487452504515	4.767072458763	0.682797731216
H	9.046802811901	4.080717599995	1.449078009564
H	9.381144126543	5.784307079954	1.135537685097
C	10.969409546884	4.436499149946	0.396535023206
H	11.032885878337	3.425073124000	-0.063894673308
H	11.336749710379	5.134623148788	-0.388327278136
O	9.000686957353	4.356250177694	-1.646986190006

C	13.528316026299	3.156870878175	4.617499562688
H	13.782314235937	3.848481447872	5.452476097192
H	12.753449637230	2.465303301162	5.024039829181
C	14.800698308402	2.349024830994	4.245384732644
H	15.513585885524	3.038228225254	3.726299369522
H	14.531307556742	1.586853286069	3.475771177714
C	15.482117372163	1.690938461220	5.410405872692
C	15.134213501973	1.683025163832	6.770647741314
N	16.687305970253	0.995047353571	5.280591858750
C	16.137179246563	0.974085915358	7.485828504091
H	14.248760928726	2.165772639388	7.209253245901
C	17.098633800838	0.550703940933	6.534420215293
C	18.338537266288	-0.261402929211	6.766476418204
H	18.905563544453	0.183187244697	7.618309923121
H	19.023291382936	-0.210536652466	5.891251628014
C	18.073211507130	-1.758474326867	7.079849810237
H	17.824893905024	-1.919951790802	8.153339681951
H	17.174150367478	-2.116572291635	6.516952463589
C	19.218268387321	-2.691854486912	6.637589294452
C	19.395258716708	-3.970879682629	7.438790495978
H	20.101230757036	-4.658115835358	6.929571565821
H	19.798843288581	-3.719607810788	8.448061021607
H	18.418918284917	-4.478589073684	7.612089943621
O	19.917250051447	-2.421563445061	5.669276570547
N	-2.606161915981	-1.541645814924	4.140714010427
C	-3.736065029984	-0.897002708787	4.666375608072
C	-2.602401036077	-2.876896158636	4.563150424565
C	-4.463933170826	-1.830355973163	5.418750173131
C	-3.741860648279	-3.065837273363	5.347989855259
H	-4.041782700761	-4.021648585543	5.804643999181
C	-4.137827302388	0.490930724737	4.247412670672
H	-4.547725192810	1.051483286760	5.118869647167
H	-3.258394691934	1.075571272930	3.897619081549
C	-5.234847555367	0.520241920174	3.124642509205
H	-6.209839867251	0.214192058598	3.563279960324
H	-5.344620858951	1.563910201283	2.747816458333
C	-4.897706446867	-0.394076542260	1.949627390837
C	-5.574563917795	-1.737027753834	1.896506268769
H	-5.247513952650	-2.321569657828	1.013205027842
H	-5.341272366661	-2.293357409970	2.837534316472
H	-6.682031590260	-1.611871430407	1.883699509708
O	-4.074234677255	-0.069508038192	1.079196310464
C	-1.609585668725	-3.914816039315	4.112402071931
H	-1.611521198406	-3.966746137126	2.995535271418
H	-1.999558146063	-4.902304330631	4.443521684731
C	-0.135333265275	-3.786408581869	4.623134881534
H	0.301640921343	-2.817252940602	4.290188349640
H	-0.159263644875	-3.737401755418	5.734699102379
C	0.778272018049	-4.907755071433	4.224880926063
C	1.210816743373	-6.011737929912	4.991461246469
N	1.357467461744	-5.011693893316	2.956438913172
C	2.072524888738	-6.779215047070	4.156688934787
C	2.156894330588	-6.153734053141	2.905297263446
H	2.598876443634	-7.703354161150	4.440998359877
C	1.065173500880	-4.145177976018	1.814330514234
H	0.974489514613	-4.796286509044	0.917246276481

H	0.052813824947	-3.708929719490	1.962513428041
C	3.023307935595	-6.512838042712	1.731045225863
H	2.919053660690	-7.602150768739	1.518468729109
H	2.703926952983	-5.990987705333	0.801687563571
C	4.515512839108	-6.187902955972	1.985741838037
H	4.623526293391	-5.162488920645	2.418096128976
H	4.943497140695	-6.871489528646	2.753829524540
C	5.391822837559	-6.168256677507	0.727468080768
C	6.842639338884	-6.589222943598	0.957998696954
C	7.769266928022	-6.498971883245	-0.267407990031
H	7.500317048560	-5.573671681632	-0.826492000645
H	7.521265732963	-7.331455363368	-0.972440485756
C	9.241533363151	-6.504041407428	0.078733961601
C	9.931403037780	-7.143036530811	1.125234450796
N	10.190667589760	-5.819052230405	-0.695136015902
C	11.315321373920	-6.781281947208	0.990753349034
C	11.458650644278	-5.967198164378	-0.141416186258
H	12.134586144332	-7.159442318670	1.620899167517
C	9.893820465043	-5.010011661983	-1.881464043415
H	9.009213308070	-5.448058741126	-2.394583538603
H	10.739879918230	-5.117154066601	-2.598136601368
H	7.221123016341	-5.960000650858	1.800567122287
H	6.813232532231	-7.621247714838	1.379281885142
O	4.965788800564	-5.792830374349	-0.359437565929
C	12.701854480594	-5.326867371623	-0.701140625306
H	12.658891540012	-5.310480065110	-1.814850136502
H	12.773833955163	-4.258950372806	-0.380437739255
C	13.998106315160	-6.057284437389	-0.253129938678
H	13.889276654330	-7.140887095892	-0.500397870556
H	14.069051781659	-6.002959304081	0.854688421931
C	15.258135086845	-5.550082278541	-0.893044210648
C	15.654529084432	-5.703543102147	-2.250328653366
N	16.285375037349	-4.891050895514	-0.220386454701
C	16.940302382112	-5.119880425449	-2.371976423749
C	17.325572100356	-4.627750197710	-1.114582206002
H	17.556832658026	-5.073154126713	-3.282261973248
C	18.612848639844	-3.980410060063	-0.714677387641
H	19.126710742983	-4.528170054786	0.110521766768
H	19.306075006912	-3.961054310885	-1.580594170111
H	18.482762747771	-2.927831212863	-0.369090290524
C	16.316438156897	-4.537251939581	1.202462000106
H	15.583046492336	-5.181065513269	1.735017405013
H	17.315818597830	-4.816427240830	1.610428694681
C	16.019571978308	-3.055880816446	1.511137738532
H	16.823273283303	-2.418014449306	1.073853690403
H	15.068422678352	-2.758906469809	1.011249866776
C	15.894260181443	-2.808624025435	3.027512408554
H	16.719344614362	-3.336655694754	3.567627020715
H	14.945820004465	-3.279080453761	3.373539405107
C	15.909294666084	-1.320685474283	3.441228125592
H	15.433566824671	-1.224321061978	4.446054620698
H	15.260005988617	-0.735095142144	2.744214546968
C	17.320106901377	-0.705509506064	3.496886511004
H	17.980119683130	-1.349668381490	4.122990740062
H	17.790763365325	-0.710228076052	2.484880882904
C	17.387252275647	0.750255079297	4.010266494385

H	18.452964049466	1.055580254918	4.122602224965
H	16.952906682574	1.441316011797	3.256618914517
C	9.655226471970	-3.524541336221	-1.557580710462
H	8.794292679394	-3.440269184793	-0.854656403111
H	10.538290892734	-3.143508346275	-0.993642604377
C	9.404449140842	-2.655551555069	-2.801921541016
H	10.315064046470	-2.654723951250	-3.449914533335
H	8.596518070463	-3.115115906854	-3.420506197196
C	9.000940230131	-1.200916933449	-2.482607894597
H	8.827584766808	-0.651925305396	-3.436949195502
H	8.027116752896	-1.196081041984	-1.936793082359
C	2.071366222625	-3.006240166978	1.543649969016
H	2.190467486581	-2.397110460811	2.471054209252
H	3.080299003580	-3.429218757415	1.326728196141
C	1.618769252161	-2.092319588979	0.380554671601
H	2.349105455906	-1.252047825465	0.291170922251
H	0.643429552231	-1.610965149818	0.646824813238
C	1.474786415206	-2.817075338874	-0.974529078715
H	2.466889280048	-3.234718208643	-1.276329436217
H	0.814152501164	-3.704884040104	-0.846682367292
C	2.574013370690	3.881982692166	-0.449428683588
C	4.772945748434	3.984836639734	-1.053741554420
C	4.018867173025	4.296136977771	-2.195538314921
H	1.767309639092	4.450123061502	-2.447896099264
C	2.637105543573	4.213325324207	-1.813261265432
N	3.881081696016	3.747263063940	0.004630729949
C	4.241096701741	3.370364833843	1.378207073043
H	3.571020519236	3.929668235408	2.069927335437
H	5.265186811036	3.754434129551	1.579883496377
C	4.180346426391	1.863361816120	1.715313711502
H	4.316990018192	1.783422411226	2.821312117089
H	3.155275876553	1.470451981483	1.511991004999
C	5.234560761010	0.992546996993	1.012651078989
H	5.008635136700	0.925820182267	-0.079083032259
H	6.229067069757	1.494245360307	1.083008222768
C	5.383819711059	-0.419495989319	1.605528067914
H	5.620513663529	-0.333828854742	2.693192586601
H	4.422939773320	-0.982597597691	1.552968700903
C	11.894307951717	4.551986591971	1.575429343619
C	13.003618814673	3.990901991681	3.483078895555
C	13.392226418687	5.303988544504	3.139088087719
H	12.763628590717	6.589746675594	1.392463969959
C	12.692332358537	5.640297115805	1.945099029698
N	12.088230365771	3.546114970792	2.525114953768
C	11.423996218785	2.240370942170	2.525829162288
H	11.267070884284	1.941017389710	3.586537565466
H	10.405215333370	2.356752585867	2.092134621842
C	12.162802434324	1.120039274768	1.760538330038
H	12.168895544269	1.365949397883	0.673648040318
H	13.234251105304	1.099184783532	2.068410969119
C	11.530561093600	-0.270531385805	1.979879925100
H	10.429029070206	-0.204549055274	1.806119357927
H	11.920066110019	-0.965226240096	1.200528837188
C	11.805341489724	-0.886454643443	3.368520638010
H	11.530200748034	-0.167018077799	4.175058859907
H	12.898612654048	-1.069633752528	3.483257580952

C	6.504207146647	-1.204420778125	0.896351813721
H	6.161896591843	-1.526913226930	-0.118650683808
H	7.389300527394	-0.552823943464	0.718373814745
C	6.944244902098	-2.462931413510	1.631106733883
C	11.038973561558	-2.193070696188	3.635281527385
H	11.209188679406	-2.527806538170	4.688081678788
H	9.940290986081	-2.038445057306	3.546872530132
C	11.455621872201	-3.365252596249	2.749368002978
C	0.879583902615	-1.998230955443	-2.138912950940
H	0.662640239478	-2.703098775708	-2.972428620316
H	-0.110049367147	-1.574625733371	-1.843999806745
C	1.766035473118	-0.877020430948	-2.710518932844
H	2.836228531158	-1.182083487237	-2.778751329034
H	1.472465068900	-0.667258111683	-3.770039778246
C	1.702016037834	0.477422636967	-2.034320239760
C	10.052067734433	-0.436583887973	-1.639624371353
H	10.196496352919	-0.945794874267	-0.662301761794
H	11.026671018465	-0.425856753824	-2.178866948724
C	9.625656055669	0.994437427423	-1.339212173110
O	8.286545672980	-2.643489417660	1.519424728584
H	8.577368832088	-3.474354038218	1.980249438847
O	10.465475957993	-4.288623997111	2.624727992033
H	10.806542063548	-5.073982581765	2.087361573281
O	10.087085885758	1.864041834811	-2.254957935588
H	9.756221328215	2.789278749285	-2.048764594713
O	2.723549706256	1.260545928714	-2.327409234153
H	2.607037414119	2.216972965861	-1.983921712444
O	6.208176323876	-3.228215815910	2.235419800022
O	12.556290559938	-3.493808877014	2.231621902527
O	8.922389444714	1.314705401547	-0.378501666193
O	0.756894856093	0.851543120789	-1.309929455005
C	14.864067240597	-6.376578721576	-3.337760263538
H	14.256943561586	-7.226713712633	-2.953183765781
H	14.158108255540	-5.674206119803	-3.842529649108
H	15.534099889910	-6.776186087268	-4.129310498536
C	9.404840908755	-8.075505617092	2.189457459161
H	8.816719888153	-7.555074668436	2.980147775226
H	8.753827308315	-8.874575249065	1.768911722896
H	10.247896376025	-8.587089322709	2.701899802004
C	0.821010498129	-6.328808254962	6.409588886776
H	0.683343074921	-5.413182065123	7.027630611663
H	-0.135484291969	-6.900699832420	6.463138681516
H	1.594989417704	-6.951433436335	6.908123207008
C	-5.774871213442	-1.598360969221	6.122573542490
H	-6.649323598551	-1.948129188714	5.523451310239
H	-5.814339338255	-2.151540246570	7.086695570784
H	-5.945889670103	-0.523529017299	6.353524279692
C	16.181356191071	0.714074656755	8.965765087045
H	15.810744748014	-0.306270497213	9.225206858470
H	17.213935783975	0.793700332930	9.374981541750
H	15.547147126521	1.438597317650	9.520407150792
C	14.377913930879	6.168506997008	3.877933153280
H	14.280090163910	6.072551411876	4.983086630301
H	15.435420443929	5.916648678155	3.626043005886
H	14.235339415865	7.241210640658	3.625597692679
C	4.536247973849	4.653363554318	-3.562559511087

H	4.614128249291	5.757279311868	-3.699402177817
H	5.546891072526	4.232548532054	-3.757608213993
H	3.859727308652	4.278738373168	-4.361713092664
C	-1.733485386564	5.175370401374	-0.756509248136
H	-0.815762334157	5.712665121083	-1.081880615044
H	-1.802314013887	4.245672232345	-1.372491052033
H	-2.602649137488	5.806562587708	-1.045401130128
Cu	-0.754128982364	1.291088005041	-0.288053669473
O	-2.230954975043	1.745904894532	0.812047400427
H	-2.463055844365	2.696959154934	1.038480213474
H	-3.044405904336	1.137181415782	0.944175990953

PAMBA-Cu(II)

C	6.547156385160	1.958458195649	-4.780490867052
C	7.400658918675	1.203347423160	-5.614644831958
C	8.729530144400	1.421259525218	-5.149381646546
C	8.679953966244	2.295649797097	-4.055993122608
N	7.342320968080	2.627390129702	-3.846033908569
H	9.650148518455	0.990943522210	-5.571824746320
C	9.798267405409	2.832290819970	-3.217352003629
H	9.862784272823	3.945890378713	-3.251763517364
H	9.715320085278	2.543440196215	-2.142644700271
H	10.766173246893	2.437715288705	-3.589701286457
C	6.841790466846	3.390228626244	-2.703171903409
H	7.602480325819	4.156047098344	-2.431578268156
H	5.940966907075	3.963442032761	-3.023685235718
C	6.499057224761	2.514194649431	-1.478622380295
H	5.850352739384	1.669644193060	-1.810793437973
H	7.437032640338	2.045145752986	-1.098875380218
C	5.789171651137	3.299943766129	-0.361857449581
H	6.430864640169	4.154844636561	-0.034084433020
H	4.863301281833	3.762815905568	-0.781870928201
C	5.395078743101	2.461416339943	0.872064745048
H	4.751881433698	3.090371654118	1.534410561882
H	4.748581767031	1.609880642986	0.547585523157
C	6.576374413716	1.920020937875	1.699107129167
H	7.171816306624	1.183042501348	1.110909326551
H	7.278122180834	2.755876378587	1.933885603011
C	6.103163915863	1.269605708343	3.016736069247
H	5.438837680052	0.402812794718	2.798764078984
H	5.480658359297	1.984773577220	3.596076755766
C	5.059304800675	2.117364436106	-4.871196217065
H	4.628364898886	2.435513384722	-3.895655650269
H	4.604385926363	1.122764152820	-5.091262781940
C	4.554463271976	3.102110703313	-5.983057734578
H	5.088742604109	4.075302662948	-5.892166989650
H	4.850396395627	2.698388146129	-6.976665309474
C	-0.020186328901	5.031604038781	-4.815517760960
H	0.325954737080	6.089926880084	-4.845012811739
H	-0.892551890357	4.997783103577	-5.504504466511
C	-0.577919559285	4.687525684113	-3.378773915965
H	-0.627582998650	3.582923357886	-3.258935642657
H	-1.613116178833	5.094050579414	-3.306024445784
C	0.238920921687	5.285898139639	-2.254807428619

C	0.143490641677	6.785705297618	-2.023905794834
H	-0.800608311738	7.188616901593	-2.453865170287
H	0.951749533875	7.218785247944	-2.667441082197
C	0.345124082898	7.273574636549	-0.550804081770
H	0.864081943515	6.468824068615	0.021227425146
H	1.041109259271	8.138841805539	-0.550505361660
O	1.022310696857	4.600548147357	-1.570515084930
C	-4.350956950226	7.039841255521	1.362594108071
H	-4.263124939175	6.033707524208	1.836124939357
H	-4.865985377807	7.672454834058	2.119023821560
C	-5.262200983029	6.945876864692	0.081809250212
H	-4.668876253591	6.473025238911	-0.733688119963
H	-5.460494426009	7.985093467391	-0.262948151047
C	-6.577915131426	6.250793369802	0.262692318063
C	-7.814348552110	6.788342060973	0.643067356439
N	-6.752545832502	4.873206243524	0.104760716018
C	-8.764224198918	5.732628666784	0.712005621178
H	-8.005939133187	7.851653747049	0.853848488870
C	-8.085035342050	4.541590906367	0.353438168253
C	-8.638929941544	3.138955433319	0.307262871093
H	-8.973495564972	2.858923252876	1.335883242606
H	-7.849629702108	2.405554699840	0.033299194859
C	-9.841221704745	2.929139083387	-0.641530274411
H	-10.367082734703	1.979380798149	-0.366912241553
H	-10.617344522204	3.716449931776	-0.508272022967
C	-9.520497149444	2.791254223684	-2.132681669234
C	-10.699533497475	2.953244095529	-3.078451769957
H	-10.466836031441	2.536799305414	-4.080087162024
H	-11.626013907685	2.487338242260	-2.675116958721
H	-10.918651371397	4.041794516400	-3.191354157117
O	-8.388263241079	2.545878719746	-2.541364072137
N	7.184185406198	0.802361099299	3.886710013393
C	7.971586266288	1.622343357050	4.694096205582
C	7.639273543623	-0.514657501966	3.987870834565
C	8.963769429418	0.816845682118	5.303257124507
C	8.743571114299	-0.512460667294	4.853898485767
H	9.318332848628	-1.405004931610	5.144650619615
C	7.764943919715	3.103682322162	4.877495150245
H	8.747768087103	3.611332353188	4.729688161585
H	7.093694072821	3.519094783698	4.094588342713
C	7.238944687643	3.542778925219	6.266182654365
H	7.853978333389	3.111543230183	7.094549035521
H	7.361833884385	4.647421375923	6.392715031666
C	5.782564463792	3.226218109589	6.563971200722
C	5.182803909481	3.757207724854	7.848683766919
H	4.291289987898	4.373964820463	7.586982178644
H	4.808200360357	2.904054011480	8.459006754694
H	5.892321648292	4.354219337771	8.457529353775
O	5.102061141437	2.570896527086	5.765930998312
C	6.981184184588	-1.703253143245	3.351878601006
H	5.898238613816	-1.736704613871	3.630246967142
H	7.427069602826	-2.608158228445	3.819607044971
C	7.119492709877	-1.853227010014	1.797026669093
H	6.578413271996	-1.022848524745	1.284916876080
H	8.191127534696	-1.721143498746	1.530068052821
C	6.667947933812	-3.187080773002	1.276237412483

C	7.433405487801	-4.306283363903	0.907426186046
N	5.314547169197	-3.534846472726	1.153993584543
C	6.509869620169	-5.333934896767	0.533602780342
C	5.209313550016	-4.848388644788	0.683370208047
H	6.770167280146	-6.353721642255	0.210433611956
C	4.219421991292	-2.587805178565	1.252017801871
H	3.279502896576	-3.148586998170	1.454637075109
H	4.372023879181	-1.930283966775	2.139193187182
C	3.914420490120	-5.581397064232	0.436317995400
H	4.161825642887	-6.663263138995	0.489447376938
H	3.187152611374	-5.404210664484	1.263095993010
C	3.201333629580	-5.316469648579	-0.918985463628
H	3.891691128616	-4.852689812385	-1.666384300345
H	2.904977470506	-6.278134558207	-1.409596664545
C	1.938645060039	-4.474759375548	-0.882551588614
C	1.234460518563	-4.189527502962	-2.208162743335
C	0.904232450601	-2.676445033930	-2.374290868182
H	1.867244621828	-2.126236771006	-2.442013913851
H	0.430124460157	-2.318930209909	-1.432038664716
C	0.068614498774	-2.285257975092	-3.558430253541
C	0.458815878114	-1.761860041021	-4.797253402293
N	-1.334665040340	-2.354383872910	-3.550563549063
C	-0.742721891970	-1.497843148448	-5.538452244815
C	-1.838222179410	-1.861674525904	-4.755950597265
H	-0.788290863128	-1.096952563219	-6.561425022953
C	-2.145092410309	-2.815370819865	-2.439086245467
H	-1.646347542419	-3.690713032415	-1.959375141418
H	-3.104092561734	-3.221078880542	-2.835780313824
H	1.832663523683	-4.558921450674	-3.070058243214
H	0.295477479517	-4.796225790234	-2.202699488767
O	1.481956744755	-4.041389870477	0.184063318158
C	-3.316530998977	-1.769750470520	-5.029747129656
H	-3.792322235671	-2.781899264333	-5.008677546046
H	-3.819919471064	-1.195638307728	-4.213637358548
C	-3.653494339619	-1.102436631365	-6.391117471356
H	-3.323135037612	-1.778922891342	-7.212834016281
H	-3.040563757002	-0.178777603639	-6.500490936378
C	-5.111198457241	-0.793360872450	-6.571884800965
C	-6.145425621748	-1.649982204344	-7.016718474083
N	-5.679894489899	0.446973161707	-6.274022973524
C	-7.350281088345	-0.894990878955	-6.975071506058
C	-7.052961587066	0.395862454017	-6.514463445794
H	-8.352046536165	-1.244560577033	-7.267114026318
C	-7.964209564308	1.565298829355	-6.308376728095
H	-7.665313082774	2.451561269588	-6.917367310838
H	-8.993947480103	1.290395772862	-6.618327898504
H	-8.011097346558	1.898387331978	-5.243682158970
C	-4.968433528615	1.611352652974	-5.749947337183
H	-3.917365616806	1.572936361698	-6.116195981522
H	-5.410986182508	2.529129509481	-6.200358532073
C	-4.987375241505	1.717825342536	-4.212028631200
H	-6.028567308776	1.911669817123	-3.866402697972
H	-4.702980329221	0.731970556746	-3.773106094021
C	-4.029002879649	2.797980402517	-3.678038282241
H	-4.182575598998	3.755755020913	-4.235050874932
H	-2.978572514211	2.487917814415	-3.899902132029

C	-4.187172994740	3.058167304556	-2.166028761255
H	-3.271879329959	3.568865466327	-1.775968481743
H	-4.230368561843	2.076048386108	-1.635182839226
C	-5.428209308481	3.894654362315	-1.808410876615
H	-5.308830792647	4.927881962223	-2.212583433977
H	-6.337946400957	3.473541349034	-2.288762028220
C	-5.678859588597	3.960650734480	-0.290470292714
H	-5.914903613266	2.945326113935	0.098894389943
H	-4.749539048547	4.267417287751	0.242302305933
C	3.061659804342	3.274546451421	-5.960085041847
C	1.014534678845	4.095302062171	-5.367364974587
C	0.796859426164	2.963598225166	-6.173361554850
H	2.284824930221	1.596583973548	-7.192086784292
C	2.083794570725	2.460485116707	-6.539833921482
N	2.398726681096	4.261085112743	-5.228431860414
C	3.046604762832	5.349735035448	-4.504874235500
H	4.124710321344	5.091072294985	-4.398407233720
H	2.662886126099	5.388555738321	-3.460155319981
C	-0.921239098456	7.736242876416	0.113909095427
C	-3.011844218382	7.633092224795	1.037417287068
C	-2.662626906885	8.991026340194	0.931020282192
H	-0.779871297710	9.951565814959	0.123902161734
C	-1.353248849936	9.040915795173	0.356529037249
N	-1.945785326769	6.876999253106	0.523733192393
C	-1.774157317494	5.440582622615	0.696545769521
H	-1.473739705377	4.967087554978	-0.265841902264
C	2.933557237434	6.725618307109	-5.153139620503
C	3.120651248317	7.879097996622	-4.355801474417
C	2.680199998198	6.881588099408	-6.531986306271
C	3.048412194856	9.159418507335	-4.921017807362
H	3.339843086172	7.772109470841	-3.275969315106
C	2.606268481580	8.163979477116	-7.102186420551
H	2.533182041982	5.991420216296	-7.169700646683
C	2.785982213739	9.312813501868	-6.300617699399
H	3.192371456447	10.065334246889	-4.306564491779
H	2.407328492369	8.276434036020	-8.181181637344
C	4.025411196330	-1.720567940795	0.008067208843
C	3.052336953061	-0.699767681670	0.014734142154
C	4.804942855962	-1.914835354613	-1.154649585702
C	2.869904360209	0.127892929957	-1.115372534855
H	2.451610510148	-0.519309512845	0.926038002396
C	4.617609489351	-1.099371215193	-2.286993637874
H	5.579882090067	-2.701932281844	-1.166852479388
C	3.660537123856	-0.074784296990	-2.269731619265
H	5.237612028051	-1.256297781019	-3.187621104572
H	3.506972821202	0.564617469281	-3.152368904726
C	-2.453940559448	-1.786491842979	-1.353893798724
C	-3.256924418548	-2.183103068725	-0.253775210278
C	-1.955655446036	-0.465562456050	-1.395826291839
C	-3.534400066403	-1.294604294770	0.794786195370
H	-3.681964593714	-3.204211477354	-0.226537032428
C	-2.235974703214	0.453936907836	-0.341421490084
H	-1.387039709137	-0.127770837043	-2.281922372341
C	-3.029598534645	0.028603016036	0.769404653168
H	-4.159572751547	-1.601942857138	1.651536169517
H	-2.000231097964	1.525687634290	-0.471681065899

H	-2.767703928188	4.998546807763	0.933841486869
C	-0.772956787582	5.057041042745	1.782932101937
C	-0.651239726156	5.809191272301	2.973077451720
C	0.060715972768	3.933187844688	1.597783829146
C	0.280113420650	5.440642423636	3.957761717840
H	-1.280499289858	6.705923426496	3.121900207821
C	0.997458060375	3.565627679625	2.578296837605
H	0.001858988070	3.352797376952	0.661862295106
C	1.109994459774	4.312538721448	3.774615010818
H	0.387704075879	6.032491032755	4.883543956639
H	1.685702062203	2.720686045348	2.397824855849
C	2.709364177337	10.711761889755	-6.848935742621
C	1.875116543684	1.241390125837	-1.073953298216
C	-3.391190243495	0.929775362984	1.916102581186
C	2.133810105204	3.972677536955	4.827422012256
O	2.476494277079	10.733409155744	-8.188963239394
H	2.450784039769	11.683087405807	-8.453917032761
O	2.179523920963	2.257890879536	-1.860509890896
H	1.555365643326	3.069892797567	-1.755984940946
O	2.680795987220	2.751500677922	4.635749195151
H	3.454540962464	2.635628329512	5.275788747457
O	-2.969576883191	2.219200151669	1.735187568512
H	-3.223933793423	2.706846427436	2.554627420993
O	2.839611624223	11.719094033683	-6.172545074050
O	2.431189479424	4.727031958802	5.744945557961
O	-4.002983756366	0.567869447322	2.905215251594
O	0.856083185680	1.233607379033	-0.347265692425
C	-5.993137013245	-3.084931821804	-7.440376473504
H	-5.000885532190	-3.286037573867	-7.902862877755
H	-6.099948878284	-3.790267863286	-6.581767704182
H	-6.769480873541	-3.368745837903	-8.183507304193
C	1.860655283020	-1.510284549098	-5.285366728682
H	2.039436923575	-0.425857709847	-5.478234677413
H	2.629218131066	-1.851852916158	-4.559064941466
H	2.058272713113	-2.042308738482	-6.244003645653
C	8.932274219686	-4.433594596457	0.941025098086
H	9.281783137123	-4.969361810907	1.854575151059
H	9.307643166121	-5.013186326394	0.068908190634
H	9.443133686599	-3.445989025474	0.928681642709
C	10.032527828513	1.281671571373	6.254217010909
H	9.672465418011	1.327783371199	7.310022098772
H	10.901613059099	0.589176554368	6.247400065665
H	10.414666201375	2.295717030233	5.997853091849
C	-10.208710023849	5.866747761794	1.113982056007
H	-10.889577951142	5.974250281702	0.235994235828
H	-10.568520698081	4.992534704204	1.701860692965
H	-10.361109514988	6.770702773576	1.742239926507
C	-3.521775303589	10.172147305281	1.293287886666
H	-4.061493545532	10.584658484933	0.408122968993
H	-4.290751783701	9.916212956437	2.054757310156
H	-2.908807765038	11.002745456105	1.707088324602
C	-0.532234043073	2.379999355678	-6.572652856344
H	-0.835431140405	1.539698368223	-5.901919402237
H	-1.353028742087	3.131923034763	-6.548009605771
H	-0.496876938191	1.967543085827	-7.605412483623
C	6.982936225497	0.361547472095	-6.789296745046

H	6.965407668745	0.945443096860	-7.740250105702
H	7.687241735773	-0.483747225710	-6.949168045253
H	5.964657584614	-0.070772166699	-6.658721901090
Cu	-0.385590713631	-0.150860514413	0.594629325284
O	-0.255634639467	-2.177761189483	1.193165493198
H	-1.143501996203	-2.598326773828	1.142177071742
H	0.360737086216	-2.849875848521	0.768755334891
O	-0.178953504909	0.582081115590	2.652878845749
H	0.164033910846	0.064288505613	3.410469339706
H	0.013525186007	1.525760455179	2.865930891408
